# Photolytic, radical-mediated hydrophosphination: a convenient post-polymerisation modification route to P-di(organosubstituted) polyphosphinoboranes [RR′PBH_2_]_*n*_†
†Electronic supplementary information (ESI) available. See DOI: 10.1039/c9sc01428d


**DOI:** 10.1039/c9sc01428d

**Published:** 2019-06-06

**Authors:** Alastair W. Knights, Saurabh S. Chitnis, Ian Manners

**Affiliations:** a School of Chemistry , University of Bristol , Cantock's Close , BS8 1TS , UK; b Department of Chemistry , Dalhousie University , Halifax , NS B3H 4R2 , Canada; c Department of Chemistry , University of Victoria , Victoria , BC V8W 2Y2 , Canada . Email: imanners@uvic.ca

## Abstract

Polymers with a phosphorus-boron main chain have attracted interest as novel inorganic materials with potentially useful properties since the 1950s. Although examples have recently been shown to be accessible *via* several routes, the materials reported so far have been limited to P-mono(organosubstituted) materials, [RHPBH_2_]_*n*_, containing P–H groups. Here we report a general route for the post-polymerisation modification of such polyphosphinoboranes giving access to a large range of previously unknown examples featuring P-disubstituted units. Insertion of alkenes, R′CH=CH_2_ into the P–H bonds of poly(phenylphosphinoborane), [PhHPBH_2_]_*n*_ was facilitated by irradiation under UV light in the presence of the photoinitiator 2,2-dimethoxy-2-phenylacetophenone (DMPAP) and (2,2,6,6-tetramethylpiperidin-1-yl)oxyl (TEMPO) under benchtop conditions giving high molar mass, air-stable polymers [PhR′PBH_2_]_*n*_ with controlled functionalisation and tunable material properties. The mechanistic explanation for the favourable effect of the addition of TEMPO was also investigated and was proposed to be a consequence of reversible binding to radical species formed from the photolysis of DMPAP. This new methodology was also extended to the formation of crosslinked gels and to water-soluble bottlebrush copolymers showcasing applicability to form a wide range of polyphosphinoborane-based soft materials with tunable properties.

## Abstract 2

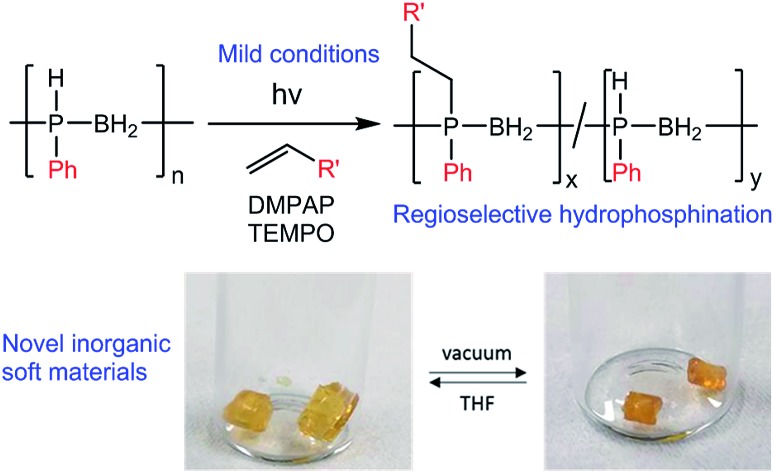
New, air-stable inorganic soft materials are accessible under mild conditions *via* TEMPO-mediated radical hydrophosphination of alkenes using polyphosphinoboranes.

## Introduction

Polymers featuring elements other than carbon in the main chain are attracting widespread interest as functional soft materials with an expanding range of applications. These macromolecules possess attributes that complement those of easily processed state of the art organic polymers by introducing additional features such as enhanced thermooxidative stability, low temperature elasticity, flame retardancy, tunable optoelectronic properties, and the ability to form ceramic films and fibers on pyrolysis.^1^

**Scheme 1:**
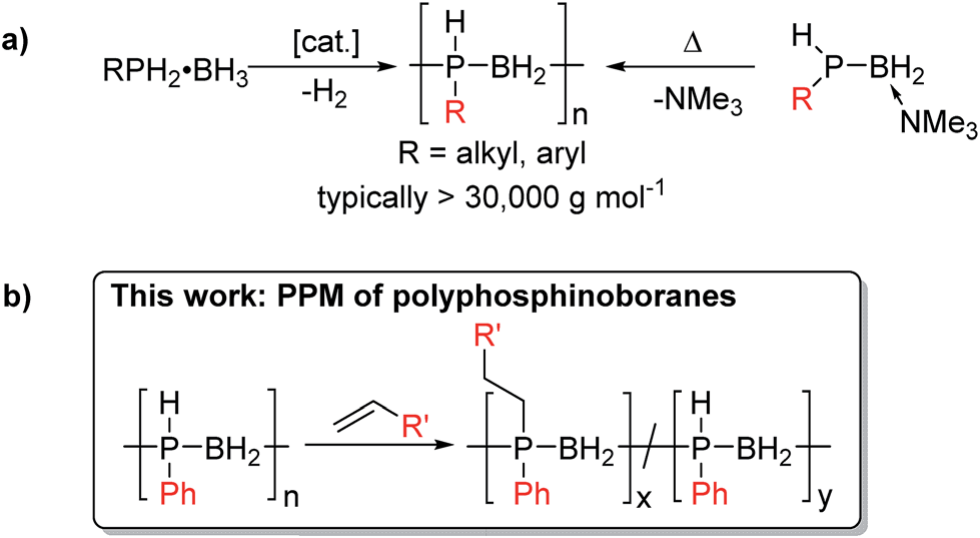
(a) Synthesis of high molar mass derivatives of [RHPBH_2_]_*n*_. (b) Post-polymerisation modification as a strategy to access high molar mass P-disubstituted derivatives of [RR′PBH_2_]_*n*_.

Polyphosphinoboranes, [RR′PBH_2_]_*n*_ are formally isoelectronic with polyolefins, and have recently emerged as a new class of inorganic polymers,^2^ with potential uses as precursors to PB semiconductor-based ceramics, etch-resists, flame-retardant materials, and as piezoelectrics.^3^ The development of new and improved routes to high molar mass polyphosphinoboranes is therefore an expanding area of research.^4^ It is now possible to access several derivatives of P-monosubstituted polyphosphinoboranes [RHPBH_2_]_*n*_ where R is an alkyl or aryl substituent *via* catalytic dehydrogenation using Rh, Ir or Fe precatalysts or thermally-induced Lewis base elimination routes (Scheme 1a). In contrast, examples of P-disubstituted polyphosphinoboranes (*i.e.* [RR′PBH_2_]_*n*_, R and R′ ≠ H) are extremely scarce. Early work in the 1950s and 1960s claimed the formation of polymeric materials *via* thermally-induced dehydrocoupling of phosphine-borane adducts R_2_PH·BH_3_ at *ca.* 200 °C, often in the presence of additives such as amines, which were suggested to prevent cyclisation.^5^ However, the products were not unambiguously characterised and, where reported, yields and molar masses were very low. Attempts to apply current catalytic routes towards P-disubstituted polyphosphinoborane targets by dehydrocoupling of secondary phosphine boranes, RR′PH-BH_3_, have been unsuccessful to date, yielding instead small rings or oligomeric materials.2b,4a,c,e,^6^ High molar mass P-disubstituted polyphosphinoboranes would be devoid of P–H bonds and are likely to be the most thermally and environmentally robust and therefore the most realistically useful in applications. Strategies to access these materials are therefore of substantial interest.

Post-polymerisation modification (PPM), for example, by activation of main-chain E–X (X = halogen, H) bonds of inorganic polymers such as polysiloxanes,^7^ polyphosphazenes,^8^ polysilanes,^9^ and polyferrocenylsilanes,^10^ is a well-known strategy for functionalising these polymers allowing the tuning of diverse physical and chemical properties. Indeed, the broad scope of PPM for polydihalophosphazenes is vital to the applications of polyphosphazene-based materials.^11^ This methodology has also been used to synthesise bottlebrush polymers^12^ and polyphosphazene gels which have interesting elastomeric properties.11a,c

We envisioned that a PPM approach involving conversion of preformed high-molar mass polyphosphinoborane [RHPBH_2_]_*n*_ to the target disubstituted [RR′PBH_2_]_*n*_ polymers would overcome the limitations associated with existing synthetic routes and give access to a more robust and tunable class of P-disubstituted polyphosphinoboranes. Herein, we report conditions under which a broad range of alkenes undergo insertion into the P–H bonds of poly(phenylphosphinoborane) to yield high-molar mass derivatives of [PhR′PBH_2_]_*n*_ (R′ ≠ H, Scheme 1b). In addition, we disclose the extension of this hydrophosphination approach to prepare crosslinked elastomers and water-soluble materials based on polyphosphinoborane backbones.

## Results and discussion

### Hydrophosphination of 1-octene using [PhHPBH_2_]_*n*_

The hydrophosphination of alkenes with primary and secondary phosphines is a well-studied reaction for which numerous catalytic and radical based protocols have been reported.^13^ This addition is analogous to the ubiquitous thiol–ene addition reaction and has recently been exploited for the synthesis of phosphorus-containing network polymers.^14^ Interestingly, the insertion of alkenes into P–H bonds of phosphine-borane adducts (RR′HP-BH_3_; R = Ph, R′ = Ph or Me) has also been reported by Gaumont and coworkers,^15^ providing a model for the putative addition of alkenes to P-monosubstituted poly(phenylphosphinoborane) [PhHPBH_2_]_*n*_ (**1**).

For all of our investigations, **1** was synthesised *via* previously reported iron-catalysed dehydrocoupling of phenylphosphine-borane (PhH_2_P-BH_3_).4*a* PhH_2_P-BH_3_ was heated to 100 °C in toluene for 20 h in the presence of 1 mol% [FeCp(CO)_2_OTf], yielding polymer as a pale yellow solid with a molar mass of around 68 000 Da and a PDI of 1.5. The discolouration of this polymer is reported to come from residual iron species remaining despite repeated precipitation from DCM into cold pentane (–78 °C).4*h*

**Fig. 1:**
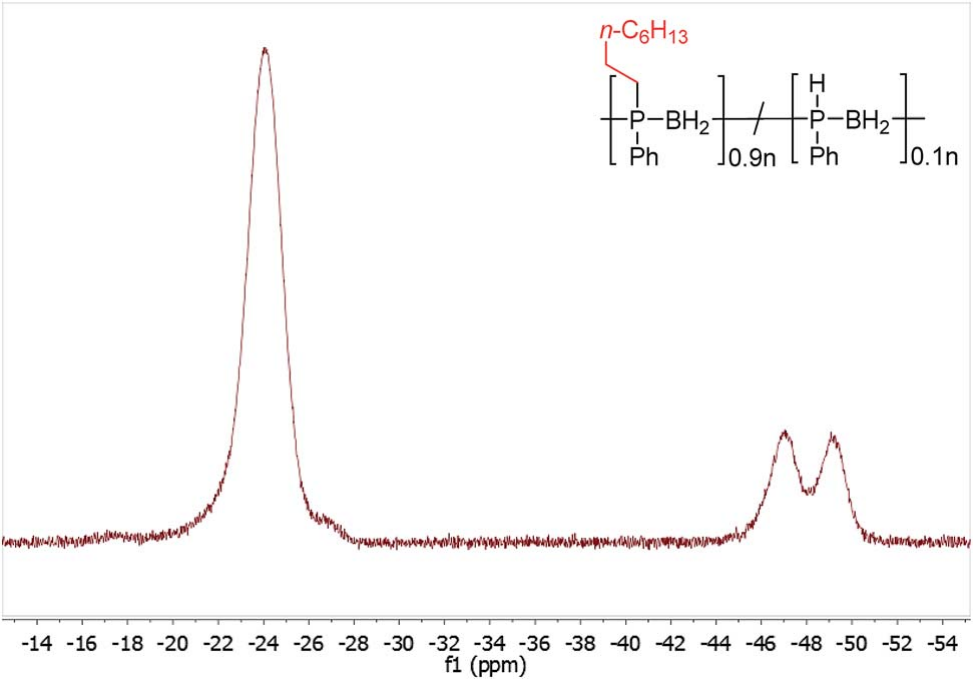
^31^P NMR spectrum (122 MHz, CDCl_3_) after PPM of **1** with 1-octene by UV irradiation for 20 h at 20 °C.

Initial studies showed that, unlike for the aforementioned phosphine-borane adducts studied by Gaumont, heating **1** (0.2 mmol) with 1-octene (0.2 mmol) in THF (0.5 ml) at 60 °C for 24 h did not result in detectable insertion of the alkene into the P–H bonds of the polymer based on ^31^P NMR analysis. However, when the reaction mixture was irradiated under UV light for 20 h at 20 °C (Table 1, entry 1), a single peak emerged in its ^31^P NMR spectrum at *δ* = –23.5 ppm with no apparent ^1^*J*_PH_ coupling (*cf. δ* = –48.9 ppm, ^1^*J*_PH_ = 349 Hz for **1**). The ^1^H NMR spectrum of the reaction mixture showed a significant reduction in the intensity of the P–H resonances and emergence of a number of broad peaks in the 0.8–1.3 ppm region corresponding to new aliphatic protons. These spectroscopic data are consistent with insertion of 1-octene into the P–H bond of **1**. Analogous to Gaumont's work with phosphine-borane adducts,^15^ the emergence of a single peak in the ^31^P NMR spectrum suggests that exclusive anti-Markovnikov addition had taken place within the NMR detection limit. Integration of the resonances in the ^31^P NMR spectra indicated around 90% conversion to the P-disubstituted species (Fig. 1) giving a random copolymer consisting of [Ph(octyl)PBH_2_] and [PhHPBH_2_] units.

**Table 1:** Effect of reaction conditions on the hydrophosphination reaction of 1-octene with **1**

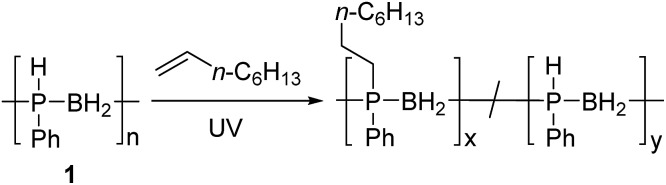				
Entry^*a*^	Additives	Solvent	Time	Conversion^*b*^ (%)
**1**	None	THF	20 h	90
**2**	DMPAP (10 mol%)	THF	1 h	90
**3** ^*c*^	DMPAP (10 mol%)	THF	1 h	91
**4** ^*c*^	None	THF	24 h	35
**5**	DMPAP (1 mol%)	THF	1 h	62
**6**	DMPAP (30 mol%)	THF	1 h	90
**7** ^*d*^	DMPAP (10 mol%)	THF	1 h	65
**8**	DMPAP (10 mol%)	THF	0.25 h	75
**9**	DMPAP (10 mol%)	Chlorobenzene	0.25 h	69
**10**	DMPAP (10 mol%)	Toluene	0.25 h	87
**11**	DMPAP (10 mol%)	1,2-Dichlorobenzene	0.25 h	86
**12**	DMPAP (10 mol%)	THF	1 h	0
	TEMPO (100 mol%)			
**13**	DMPAP (10 mol%)	THF	1 h	90
	TEMPO (10 mol%)			
**14**	DMPAP (10 mol%)	THF	1 h	88
	Di-*tert*-butyl nitroxide (10 mol%)			

^*a*^All reactions were carried out with 0.2 mmol of [PhPHBH_2_]_*n*_ and one equivalent of 1-octene in a borosilicate NMR tube in 0.5 mL solvent and irradiated under UV light at 20 °C unless stated otherwise. UV irradiation was carried out using a 125 W medium-pressure mercury lamp.

^*b*^Determined by ^31^P NMR integrations, conversion = *x*/(*x* + *y*) × 100.

^*c*^2 mmol of [PhPHBH_2_]_*n*_, one equivalent of 1-octene and 5 mL THF.

^*d*^Reaction carried out at 0 °C.

The molar mass of the product was determined by gel permeation chromatography (GPC) using polystyrene standards. A bimodal distribution was observed (*M*_n_ = 83 000 Da, PDI = 1.21 and *M*_n_ < 3000 Da) with a high molar mass polymer/low molar mass polymer peak ratio of 3 : 7.4*a* We interpret the presence of these two fractions as evidence that growth in molar mass by alkene addition is accompanied by main-chain cleavage (*vide infra*). We postulate that this chain cleavage is caused by undesired radical-induced side reactions such as backbiting and β-scission reactions – processes commonly invoked in the photodegradation of organic polymers.^16^ Analysis of the above reaction mixture by NanoSpray electrospray ionisation mass spectrometry (ESI-MS, positive mode, DCM solvent), showed a repeat unit of 234.2 *m*/*z*, which corresponds to a successive loss of [Ph(octyl)PBH_2_]. As expected for conversion of 80–90%, repeat units of 122.0 *m*/*z* corresponding to loss of [PhHPBH_2_] were also observed. The maximum observed *m*/*z* was around 3000, much lower than that observed by GPC; however, this is analogous with previous characterisation of polyphosphinoboranes4a,h and polyaminoboranes,^17^ and is a noted limitation of ESI-MS for molar mass determination of these polymers.^18^ Matrix-assisted laser desorption/ionization time of flight mass spectrometry (MALDI-TOF MS) was also undertaken in an attempt to overcome the low *m*/*z* detection limit of ESI-MS; however, no high molar mass fraction was detected suggesting problems with the ionisation of these materials under MALDI conditions.

A limitation of this methodology is that the hydrophosphination was slow, requiring 20 h to achieve 90% conversion. A variety of different conditions was therefore investigated to optimise this reaction (Table 1). Given the success of UV-promoted hydrophosphination (Table 1, entry 1), the introduction of a photoinitiator was investigated: addition of 10 mol% 2,2-dimethoxy-2-phenylacetophenone (DMPAP) to the reaction mixture and irradiation under UV light in THF showed a marked increase in reaction rate (90% conversion in 1 h, entry 2). Significantly, the reaction could be scaled up to 2 mmol without loss of activity (entry 3); whereas, for UV irradiation without an initiator, a drastic reduction in reaction rate was observed upon scale up (entry 4). Decreasing the amount of DMPAP to 1 mol% led to a slower reaction rate (entry 5) and increasing the amount of DMPAP to 30 mol% did not accelerate the reaction further (entry 6). Lowering the reaction temperature to 0 °C also resulted in a lower conversion after 1 h (entry 7). The reaction proceeded equally well in THF or chlorobenzene, and a slight increase in conversion after 0.25 h was observed when using toluene or 1,2-dichlorobenzene (entries 8–11). Changing the solvent did not have a significant effect on the molar mass profile of the resulting polymer according to GPC analysis and because of the higher volatility and therefore easier removal of THF from the polymer products, THF was used for all subsequent reactions. Yields and molar masses obtained when reactions are carried out in air were comparable to those obtained using dry and degassed solvents under a nitrogen atmosphere.

**Fig. 2:**
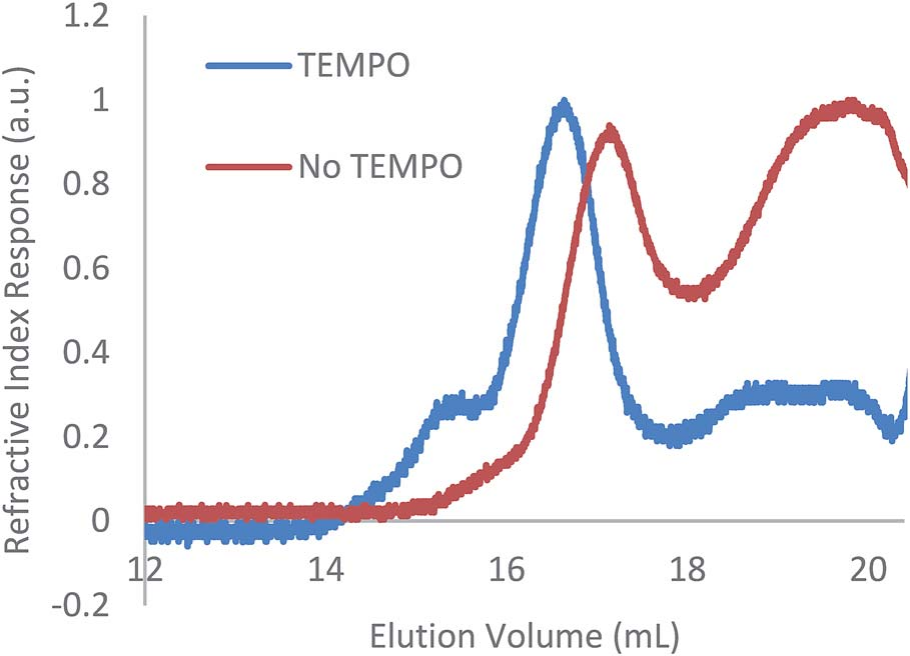
GPC chromatograms of the reaction of **1** with 1-octene in the presence of DMPAP (10 mol%) with and without the addition of TEMPO (10 mol%).

As with the case in which no photoinitiator was used, a bimodal molar mass distribution was observed upon analysis of the polymer product of entry 2 by GPC (Fig. 2). In an effort to minimise any molar mass decline accompanying this reaction, an analogous reaction was attempted using blue light instead of UV light; however, no reaction was observed (ESI Table S1, entry 1†). While the targeted hydrophosphination did not occur under these conditions, use of blue light irradiation together with photocatalyst 9-mesityl-10-methylacridinium perchlorate and diphenyliodinium triflate did result in the desired reaction taking place (25% conversion after 16 h) (ESI Table S1, entry 2†); however, given the sluggish nature of this reaction, this methodology was not pursued further.

TEMPO is well known to reversibly bind to organic radical species leading to its pioneering use in the field of nitroxide-mediated polymerisation (NMP).^19^ This reversible binding establishes an activation–deactivation equilibrium which reduces the concentration of active radical species giving a more controlled polymer growth. Given the success of NMP protocols to control radical reactions, we investigated the effect of addition of TEMPO to the hydrophosphination of 1-octene with **1**. When 100 mol% of TEMPO was added to an NMR tube containing **1** (0.2 mmol), 1-octene (0.2 mmol), DMPAP (0.02 mmol) and THF (0.5 mL), no reaction was observed after irradiation for 1 h (Table 1, entry 12). However, when instead, 10 mol% of TEMPO was added under otherwise analogous reaction conditions, the conversion after 1 h was comparable to the case where no TEMPO was added (compare entries 13 and 2). Furthermore, upon characterisation of the molar mass of the polymer using GPC, it was now found that significantly more high molar mass material remained (peak ratio 7 : 3 high molar mass polymer/low molar mass polymer), suggesting that polymer degradation during the course of the reaction was significantly reduced (Fig. 2). We postulate that the TEMPO acts to reduce the concentration of reactive radicals *via* reversible binding to the radical species produced from the photoinitiator resulting in a more controlled hydrophosphination without detrimental side reactions that cause chain cleavage. A similar degree of conversion was found when an alternative nitroxide, di-*tert*-butyl nitroxide, was used in place of TEMPO (entry 14).

We also found that it was possible to carry out the hydrophosphination of 1-octene using **1** thermally at 60 °C in THF using 10 mol% AIBN as an initiator. This thermally-induced hydrophosphination is significantly slower than the UV mediated version (taking 27 h to reach 90% conversion, Fig. S1†); however, this allowed for convenient monitoring of the reaction by ^31^P NMR (*vide infra*).

### Mechanistic studies

**Scheme 2:**
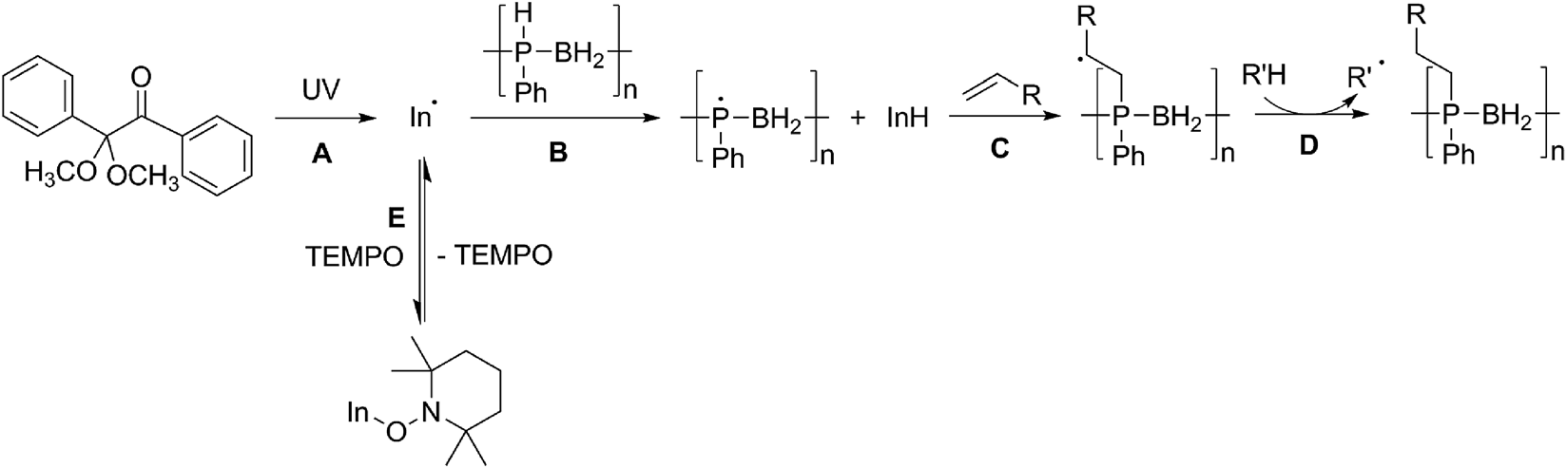
Proposed reaction mechanism for the UV-induced hydrophosphination of alkenes using **1** in the presence of DMPAP (and the effect of addition of TEMPO to the reaction mixture).

We propose that the reaction of poly(phenylphosphinoborane) and 1-octene in the presence of 10 mol% DMPAP and irradiation under UV light takes place *via* a radical chain reaction in which a radical initiator (In˙) forms from the photolysis of DMPAP (Scheme 2A), and subsequently abstracts a hydrogen atom from phosphorus on the polymer chain (Scheme 2B). This then adds to the alkene in an anti-Markovnikov fashion to give the most stable secondary radical based on the alkyl chain (Scheme 2C). To continue the radical chain reaction, a hydrogen is then abstracted from another position on the polymer chain (Scheme 2D). This is analogous to the mechanism reported by Gaumont and co-workers for the microwave irradiation-induced hydrophosphination of alkenes using secondary phosphine-boranes.^15^

**Fig. 3:**
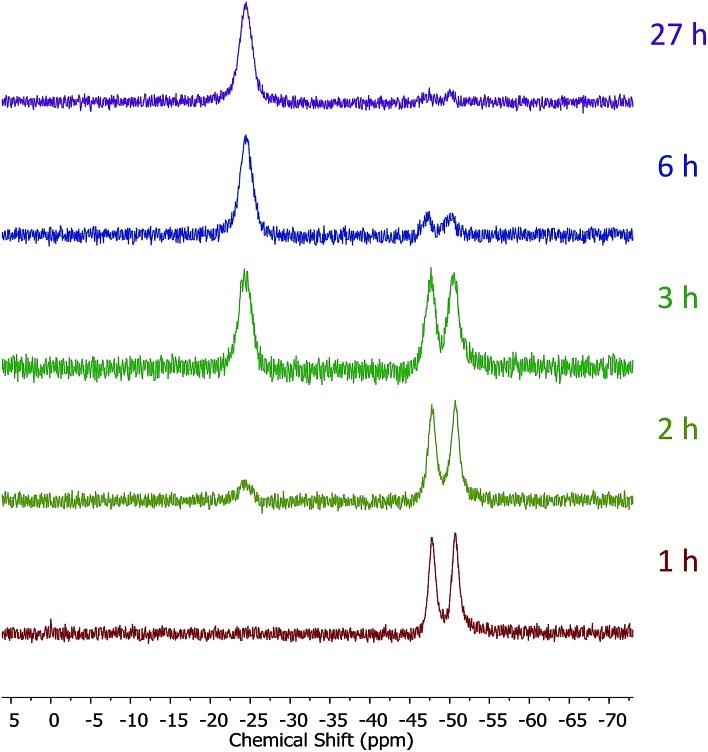
^31^P NMR (122 MHz, *in situ* in THF) spectra showing the progress of the hydrophosphination reaction between 1-octene and **1** in the presence of AIBN and TEMPO at 60 °C.

**Fig. 4:**
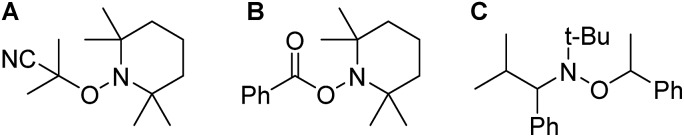
Chemical structures of adducts (A) formed by the reaction of AIBN and TEMPO (B) formed by the reaction of DMPAP and TEMPO (C) a commercially available and commonly used NMP initiator.

Introduction of TEMPO into this system has an interesting effect: UV irradiation of **1** (0.2 mmol), 1-octene (0.2 mmol), DMPAP (0.02 mmol) and THF (0.5 mL) alone at 20 °C, shows 75% conversion from **1** to the P-disubstituted polymer after just 10 minutes (determined by ^31^P NMR spectroscopy of the crude reaction mixture). However, in contrast, when 10 mol% TEMPO was present in an analogous reaction mixture, there was minimal conversion to the P-disubstituted polymer after 10 minutes of UV irradiation (Fig. S2†). Nevertheless, analysis by ^31^P NMR spectroscopy of both reactions after 1 h of irradiation shows comparable degrees of conversion of around 90% (Fig. S3†). This suggests that the addition of TEMPO causes an induction period for the hydrophosphination reaction. We also explored an analogous thermal reaction using AIBN and TEMPO wherein an NMR tube were charged with **1** (0.1 mmol), 1-octene (0.1 mmol), AIBN (0.01 mmol), TEMPO (0.01 mmol) and THF (0.5 mL) and was placed in an oil bath at 60 °C. The reaction was monitored by ^31^P NMR spectroscopy. A clear induction period was observed, with no detectable conversion by ^31^P NMR spectroscopy after 1 h but around 10% conversion after 2 h, with continually increasing conversion thereafter (Fig. 3). We postulate that the induction periods that we observe are caused by reversible reaction of TEMPO with the radical species produced from the photodegradation of DMPAP under UV light (Scheme 2E) or by thermal degradation of AIBN. The adducts formed could then break down initiating the hydrophosphination reaction. The formation of the 2-cyanopropyl-TEMPO adduct (Fig. 4A) has been reported previously from the heating a solution of AIBN and TEMPO in toluene,^20^ and so it is plausible that we are also forming this species prior to any reaction with the polymer. We also attempted to isolate an adduct between DMPAP and TEMPO. The photodegradation of DMPAP has been reported to yield several products,^21^ a number of which could conceivably react with TEMPO complicating any investigation. Nevertheless, analysis of the crude reaction mixture after the irradiation of equimolar amounts of DMPAP with TEMPO in THF by ESI mass spectrometry showed signals that correspond to 2,2,6,6-tetramethylpiperidin-1-yl benzoate fragments (Fig. 4B), as well as hydrogenated TEMPO supporting our hypothesis that an adduct forms between TEMPO and radicals derived from DMPAP (Fig. S4†).

Since these proposed adducts closely resemble alkoxyamine compounds that are commonly used as initiators in NMP,^22^ we sought to determine if alkoxyamines could facilitate the reaction of 1-octene with **1**. Heating an NMR tube charged with **1** (0.1 mmol), 1-octene (0.1 mmol), toluene (0.5 mL) and 0.01 mmol of the commercially available *N-tert*-butyl-*N*-(2-methyl-1-phenylpropyl)-*O*-(1-phenylethyl)hydroxylamine (Fig. 4C) to 100 °C, resulted in the desired hydrophosphination reaction taking place, albeit much more slowly than using our photoinitiated system (Fig. S5†). Significantly when this alkoxyamine was used, no induction period was observed supporting our assertion that adduction formation is involved in the first step of the photoinitiated hydrophosphination in the presence of DMPAP and TEMPO.

In nitroxide mediated polymerisations it is generally accepted that the nitroxide is able to reversibly bind to the growing polymer chain and this mediates the reaction resulting in a controlled polymer growth. In order to test whether TEMPO is binding to phosphorus-based radicals on the polyphosphinoborane main chain, **1** (0.2 mmol) was irradiated with DMPAP (0.2 mmol) and TEMPO (0.2 mmol) in THF (0.5 mL) at 20 °C. Analysis of the crude reaction mixture by ^31^P NMR spectroscopy after 4 h, showed the emergence of a very minor signal at 120 ppm which we tentatively assign to the polymer bound to TEMPO (Fig. S6†) due to the similarity in chemical shift to the recently reported Ph_2_POTEMP (^31^P *δ* = 110.8 ppm);^23^ however, no evidence of binding of TEMPO to the polymer chain could be observed by mass spectrometry. Addition of 1-octene and continued irradiation resulted in the disappearance of this signal at 120 ppm and the emergence of the signal at –23.5 ppm which corresponds to the hydrophosphination of 1-octene by **1** (Fig. S7†).

### Large scale syntheses and properties of P-disubstituted polyphosphinoboranes

**Scheme 3:**
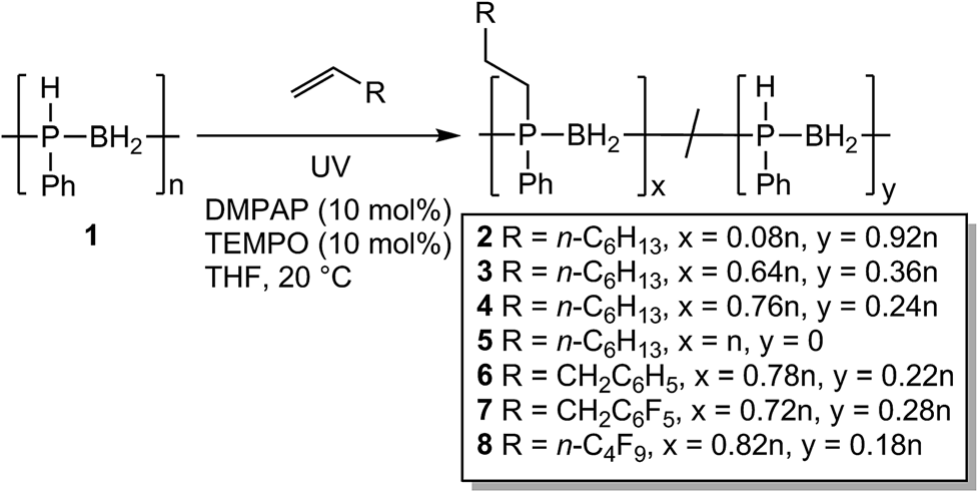
Reaction conditions for the hydrophosphination of alkenes with **1**.

Following the success of this new hydrophosphination methodology, we targeted the isolation of a series of polymers to investigate the difference in their physical properties. To this end we targeted various degrees of substitution of poly(phenylphosphinoborane) using 1-octene by varying the reaction stoichiometry (0.1 eq. 1-octene – polymer **2**, 0.6 eq. – polymer **3**, 1 eq. – polymer **4**, and 2 eq. – polymer **5**) (Scheme 3). We also targeted other alkenes: allylbenzene (1 eq. – polymer **6**), allyl pentafluorobenzene (1 eq. – polymer **7**), and 1*H*,1*H*,2*H*-perfluorohexene (1 eq. – polymer **8**). The synthesis of these polymers followed the same procedure, **1** (2 mmol), DMPAP (0.2 mmol), TEMPO (0.2 mmol), and alkene were added to a vial and dissolved in THF (5 mL). The reaction mixture was irradiated under UV light for 2 h at 20 °C for **2–4** and **6–8**. For polymer **5**, the reaction mixture was irradiated for 24 h at 20 °C. The polymers were isolated by precipitation from THF into H_2_O/isopropanol (1 : 1 v/v) at –20 °C (polymers **3**, **4**, **5**, and **8**) or from DCM into pentane at –78 °C (polymer **2**, **6**, and **7**) and then dried under vacuum at 40 °C for at least 48 h. The polymers were isolated as light-yellow solids except for **4** and **5** which were pale yellow-brown gums. The discolouration for these polymers likely originates from small amounts of residual iron species from the polymerisation of phenylphosphine-borane using [FeCp(CO)_2_OTf]. The ^11^B NMR spectra of the resultant polymers showed little change from that of the parent poly(phenylphosphinoborane) (a broad singlet at around –34 ppm). ^31^P NMR chemical shifts of these isolated polymers were found at around –24 ppm. As expected, a singlet was observed in the ^1^H-coupled ^31^P NMR spectra alongside a doublet at *δ* = –48.9 ppm corresponding to [PhHPBH_2_] units in all polymers except **5**. From the ^31^P NMR spectra, the degree of conversion to the P-disubstituted polymer could be calculated. When 1 eq. alkene was used, conversions of between 72 and 82% were observed (Table 2, polymers **4**, **6**, **7**, and **8**). Different degrees of substitution could be obtained by varying the reaction stoichiometry (compare polymers **2–5**). To obtain the fully P-disubstituted polymer **5**, a greatly extended reaction time and two equivalents of 1-octene were required. We postulate that this is due to reactive sites becoming less accessible as conversion approaches 100%. The successful incorporation of the alkene was confirmed by ESI-MS and for each polymer, fragments corresponding to [PhRPBH_2_] repeat units could be detected. The molar masses of these polymers were determined by GPC relative to polystyrene standards and were found to range from *M*_n_ = 81 000 to 130 000 Da (PDIs = 1.1–1.9). No change in the ^31^P NMR spectra or GPC chromatograms was detected after the solid polymers were exposed to air for 6 months, indicating that these polymers are air-stable. These polymers also appear to be water-stable as addition of a few drops of water to a THF solution of these polymers (5 mg in 1 mL THF) and leaving open to air for 24 h at 20 °C also resulted in no change in the NMR spectra or GPC chromatograms.

**Table 2:** Properties of functionalised polymers

Polymer	Percentage insertion^*a*^ (%)	*M*_n_ (Da)	*T*_5%_ ^*b*^ (°C)	Ceramic yield^*c*^ (%)	*T*_g_ (°C)
**1** ^*d*^	0	68 000	180	46	38
**2**	8	130 000^*e*^	160	51	30
**3**	64	130 000	210	27	15
**4**	76	81 000	200	20	9
**5**	100	112 000	215	6	4
**6**	78	104 000	195	19	50
**7**	72	130 000	210	34	67
**8**	82	92 000	175	8	43

^*a*^Determined by integration of ^31^P NMR spectra, conversion = *x*/(*x* + *y*) × 100.

^*b*^Temperature at 5% mass loss.

^*c*^Ceramic yields were measured at 600 °C after the sample mass was stable.

^*d*^ Ref. 4*a*.

^*e*^A significant higher molecular weight shoulder was observed in the GPC chromatogram of **2**.

The thermal properties of functionalised polyphosphinoborane polymers **2–8** were investigated by thermogravimetric analysis (TGA, N_2_ atmosphere, heating rate 10 °C min^–1^) and differential scanning calorimetry (DSC, heating rate 10 °C min^–1^) (Table 2). Thermal stability was quantified by comparing *T*_5%_ – the temperature at which the polymer loses 5% of its original mass. P-Disubstituted polymers were found to have slightly higher *T*_5%_ values than **1**, except for **2** and **8** which were marginally lower. This increased thermal robustness relative to the starting [PhPHBH_2_]_*n*_ polymer is promising for further utility of these modified polymers. The thermal stability of the octyl substituted polymers increased up to around 60% insertion (compare data for polymers **1** and **3**), but little further increase was observed with additional alkene insertion (polymers **4** and **5**). The onset of mass loss has been attributed to thermally induced H_2_-loss leading to further polymer degradation pathways.3*b* These results suggest that the presence of an octyl group at every other repeat unit is sufficient to suppress the inter-chain P–H/B–H interaction required for H_2_ elimination. However, higher degrees of insertion presumably enhances P–B backbone fission due to steric pressure and the concomitant molar mass decline is likely to reduce the thermal stability of the polymer. It has also been postulated that thermally induced crosslinking is important for thermal stability of polyphosphinoboranes. As the number of P-disubstituted units increases, this would become increasingly difficult as there are both fewer sites available for crosslinking and a higher steric bulk reducing favourable interactions between polymer chains.

Reflecting the random addition of 1-octene along the polymer backbone, only one glass transition temperature (*T*_g_) was observed for **2–8** (Table 2). The *T*_g_ values for **2–5** are lower than that for **1**, which is ascribed to the presence of long alkyl side chains that increase the polymer free volume and therefore reduce *T*_g_. Consistently, the *T*_g_ values for **2–5** also show an inverse relationship with the extent of alkene insertion as expected for greater incorporation of a long alkyl chain. Polymers **4** and **5** have glass transition temperatures significantly below room temperature and are gums whereas the other polymers are glassy solids. Polymers **6–8** have *T*_g_ values that are higher than for **1**, which we tentatively ascribe to greater steric interactions between the fluorinated and/or aryl groups in the polymer side chains increasing the rigidity of the polymer.

### Crosslinked poly(phenylphosphinoborane)

**Fig. 5:**
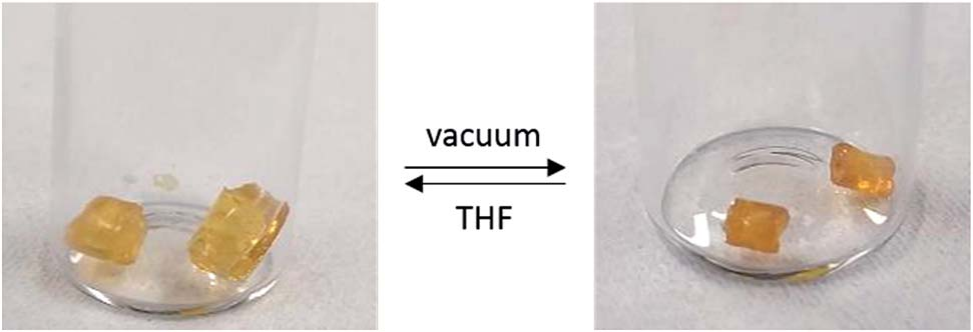
Left: Soft yellow gel obtained after soaking 1,5-hexadiene-crosslinked **1** in THF for 48 h. Right: Brittle solid obtained upon exposure of the crosslinked material to dynamic vacuum at ambient temperature for 24 h.

Following the success of the insertion of alkenes into P–H bonds of **1**, we sought to extend this methodology to other polyphosphinoborane-based soft materials. We found that when **1** is irradiated with 0.1 eq. of 1-octene, a significant shoulder is detected to the high molar mass polymer fraction (Fig. S12†). We assign this to competitive polymer cross-linking (by P–P or P–B bond formation) at low degrees of substitution. This hypothesis is supported by irradiation of **1** with 10 mol% DMPAP in the absence of alkene, which yielded material with very high molar mass (>400 000 Da, Fig. S63†). Further irradiation under these conditions results in the formation of insoluble material suggesting a higher degree of cross-linking. To investigate the potential of hydrophosphination of dienes to achieve controlled cross-linking, a solution containing **1**, 1,5-hexadiene (15 mol%), DMPAP (10 mol%), and TEMPO (10 mol%) was irradiated in THF at 20 °C for 24 h. A soft, pale yellow solid was obtained, which showed reversible organogel behaviour upon exposure to excess THF or vacuum (Fig. 5). This material was purified by repeated extraction with THF until the washings were colourless. Drying of this material under vacuum yields a pale-yellow brittle solid. This material undergoes reversible organogel swelling behaviour: if left in THF for 48 h, the material swells to 210% of its original mass; subsequent application of vacuum reverts the gel back to its brittle phase. No glass transition temperature was detected when the material was analysed by DSC. The ceramic yield of this crosslinked poly(phenylphosphinoborane) was found to be 54%, slightly higher than for non-crosslinked polyphosphinoboranes. These properties are promising for further utility of crosslinked polyphosphinoboranes and the use of different polymer precursor and crosslinking agents should yield gels with markedly different properties.

### Synthesis and characterisation of a water-soluble bottlebrush polyphosphinoborane

**Scheme 4:**
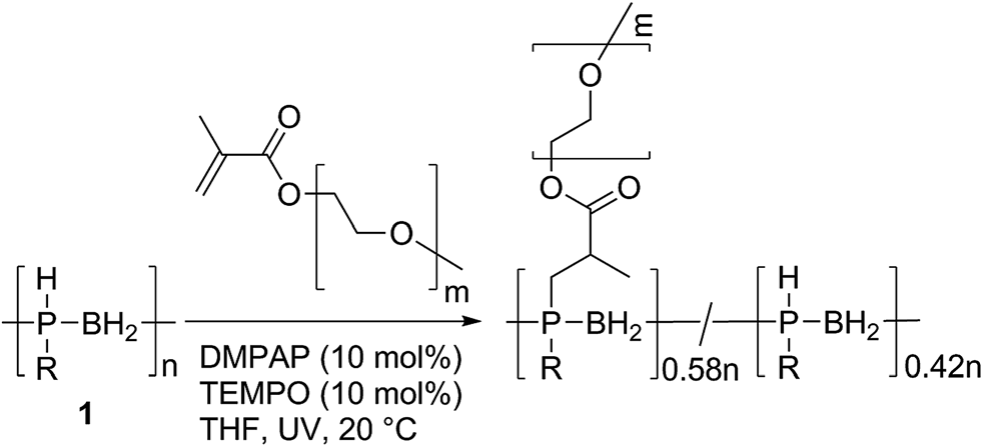
The synthesis of a bottlebrush polymer by reaction of **1** with poly(ethylene glycol) methyl ether methacrylate (*M*_n_ = 950).

We also explored the formation of a polyphosphinoborane bottlebrush polymer *via* the grafting-to reaction of **1** with two equivalents of poly(ethylene glycol) methyl ether methacrylate in the presence of DMPAP (10 mol%) and TEMPO (10 mol%) in THF (Scheme 4). After UV irradiation for 2 h and subsequent removal of THF from the resultant solution and redissolution in CDCl_3_, a grafting density of 58% was determined by integration of the ^31^P NMR spectrum. This is in the range typically found for grafting-to approaches to bottlebrush polymer formation (typically grafting densities are <60% for grafting-to approaches).^24^ This polymer was found to be water-soluble (the first water soluble polymer with a polyphosphinoborane backbone). No significant change in the chemical shifts of the ^31^P NMR peaks was observed whether CDCl_3_ or D_2_O was used as the solvent indicating that the polymer is water stable, although significant broadening of the signals is observed when D_2_O is the solvent (compare Fig. S72 and S73†). In order to remove any excess poly(ethylene glycol) methyl ether methacrylate, dialysis was performed using MW 12–14 kDa cut-off dialysis tubing in water. The successful removal of the poly(ethylene glycol) methyl ether methacrylate was confirmed by comparison of the dynamic light scattering trace for **9** and for poly(ethylene glycol) methyl ether methacrylate (Fig. S75 and S76†). The resulting polymer had a *M*_n_ of 156 000 Da and a PDI of 1.34 determined by GPC in THF. The thermal properties of this polymer were investigated by DSC and TGA. No *T*_g_ was detected by DSC analysis; however, a *T*_m_ at 40 °C was observed for the PEG side chains. The *T*_5%_ was found to be 300 °C, significantly higher than for other linear polyphosphinoboranes. This suggests that the presence of the long PEG chains imparts significant additional thermal stability, and this bodes well for future research into applications of this interesting class of polyphosphinoborane polymers.

## Conclusions

We have achieved the synthesis of P-di(organosubstituted) polyphosphinoboranes using a mild, scalable, photoinitiated process for inserting olefins into the P–H bonds of preformed P-monosubstituted derivatives under benchtop conditions. The use of DMPAP and TEMPO and UV irradiation serves to minimise molar mass decline during the course of this hydrophosphination reaction and facilitated the formation of random copolymers with controlled functionalisation as well as fully P-disubstituted derivatives. Investigations into the mechanistic reason behind the favourable effect of TEMPO addition suggested that reversible binding of TEMPO to radical species formed during the reaction could be preventing deleterious side reactions from occurring which lead to polymer degradation. The material properties of the new high molar mass polymers are tunable by the choice of alkene employed. We also describe the synthesis of the first controllably crosslinked polyphosphinoborane, a material that exhibits organogel behaviour, and the synthesis of a water-soluble bottlebrush polymer featuring a polyphosphinoborane backbone. The results described offer promise for unlocking new applications for polyphosphinoboranes and relevant work in the area is currently underway in our group.

## Conflicts of interest

There are no conflicts of interest to declare.

## Supplementary Material

Supplementary information
