# Supplementary material for: Photolytic, radical-mediated hydrophosphination: a convenient post-polymerisation modification route to P-di(organosubstituted) polyphosphinoboranes [RR′PBH2]n
Source: Chem Sci. 2019 Jun 6;10(30):7281–9. doi: 10.1039/c9sc01428d (PMC6686642; doi:10.1039/c9sc01428d)
Supplement: Supplementary file 1 [file SC-010-C9SC01428D-s001.pdf]

Electronic Supplementary Material (ESI) for Chemical Science.
This journal is © The Royal Society of Chemistry 2019

Supporting Information

Photolytic, Radical-Mediated Hydrophosphination: A Convenient Post-Polymerisation Modification Route to P-Di(organosubstituted) Polyphosphinoboranes $[RR'PBH_2]_n$

Alastair W. Knights,[a] Saurabh S. Chitnis,[a, b] Ian Manners[a,c]

[a]School of Chemistry, University of Bristol, Cantock's Close, BS8 1TS, United Kingdom

[b]Department of Chemistry, Dalhousie University, Halifax, NS B3H 4R2, Canada

[c]Department of Chemistry, University of Victoria, Victoria, BC V8W 2Y2, Canada. Email: imanners@uvic.ca

Contents

1. General procedures, reagents, and equipment....4

2. Procedures for the hydrophosphination of 1-octene using poly(phenylphosphinoborane) 6

2.1. General NMR scale reaction conditions for Table 1 6

2.2. Attempted hydrophosphination by means of irradiation with blue light 6

2.3. Hydrophosphination of 1-octene by poly(phenylphosphinoborane) in the presence of AIBN....6

3. Investigations into the mechanism of the hydrophosphination of 1-octene using poly(phenylphosphinoborane) 8

3.1. Hydrophosphination of 1-octene using poly(phenylphosphinoborane) in the presence of DMPAP vs DMPAP and TEMPO....8

3.2. Mass spectrum after the irradiation of DMPAP with TEMPO....9

3.3. Hydrophosphination reaction using N-tert-butyl-N-(2-methyl-1-phenylpropyl)-O-(1-phenylethyl)hydroxylamine 10

3.4. Irradiation of poly(phenylphosphinoborane) with DMPAP and TEMPO 10

4. Synthesis and characterisation of P-disubstituted polyphosphinoboranes 12

4.1. General procedure for isolation of modified polyphosphinoborane derivatives:....12

4.2. Synthesis and characterisation of polymer **2**....12

4.3. Synthesis and characterisation of polymer **3**....16

4.4. Synthesis and characterisation of polymer **4**....20

4.5. Synthesis and characterisation of polymer **5**....24

4.6. Synthesis and characterisation of polymer **6**....28

4.7. Synthesis and characterisation of polymer **7**....33

4.8. Synthesis and characterisation of polymer **8**....37

5. Synthesis and characterisation of crosslinked polyphosphinoborane 42

5.1. Irradiation of $[PhHPBH_2]_n$ with DMPAP (10 mol%) in the absence of alkene. 42

5.2. Synthesis and characterisation of crosslinked poly(phenylphosphinoborane) 42

5.3. Swellability of crosslinked poly(phenylphosphinoborane) 45

6. Synthesis of a water-soluble bottlebrush polymer...46

6.1. Synthesis and characterisation of bottlebrush polymer **9** 46

7. References 53

1. General procedures, reagents, and equipment

All air and water-sensitive manipulations were carried out in glassware under a nitrogen atmosphere using standard Schlenk line and glovebox techniques. Glassware was dried in an oven at 200 °C overnight prior to use. Anhydrous solvents were dried via a Grubbs design purification system. Anhydrous, deuterated solvents were purchased from Sigma Aldrich, and stored over activated molecular sieves (4Å). PhH_2-PBH_3,[1] $CpFe(CO)_2OTf$,[2] and $[PhHPBH_2]_n$[3] were synthesised via literature procedures. All other commercially available compounds were used without further purification.

Photoirradiation experiments under ultraviolet (UV) light were carried out with Pyrex-glass filtered emission from a 125 W medium pressure mercury lamp. The emission lines of the mercury lamp are: 577-579, 546, 436, 408-405, 366-365, 334, 313, 302, 297, 289, 270, 265, 254 nm. Experiments under blue light were performed using a 3 W lamp, emissions 450-495 nm.

NMR spectra were recorded using Oxford Jeol ECS 400 MHz, Bruker Nano 400 MHz, Bruker Avance III HD 500 MHz Cryo, or Varian VNMR 500 MHz spectrometers. ^{1}H and ^{13}C NMR spectra were reference to residual signals of the solvent ($CDCl_3$: ^{1}H: δ = 7.24, ^{13}C: δ = 77.0; C_6D_5H: ^{1}H: δ = 7.20, ^{13}C: δ = 128.0). ^{11}B, ^{19}F, and ^{31}P NMR spectra were referenced to external standards (^{11}B: $BF_3 \cdot OEt_2$ (δ = 0.0); ^{19}F: $CFCl_3$ (δ = 0.0); ^{31}P: 85% H_3PO_4(aq) (δ = 0.0)). Chemical shifts (δ) are given in parts per million (ppm), and coupling constants (*J*) are given in Hertz (Hz), rounded to the nearest 0.5 Hz.

Electrospary ionisation (ESI) mass spectra were recorded using a Waters Synapt G2S spectrometer by NanoSpray Ionisation. Solutions (40 µL) of approximately 1 mg mL^{-1} were loaded into the sample tray, and aliquots of 3 µL were introduced into the spectrometer using a spray voltage of 1.5 kV. Positive and negative ion spectra were recorded at a rate of one scan per second and summed to produce the final spectra. MALDI-TOF MS was performed using a Bruker Ultraflextreme running in reflector mode. Samples were prepared using a trans-2-(3-(4-tert-butylphenyl)-2-methyl-2-propenylidene) malononitrile matrix (20 mg/mL in THF) which was mixed with the polymer sample (2 mg/mL in THF), in a 10:1 (v:v) ratio. Approximately 1 µL of the mixed solution was deposited onto a MALDI-TOF MS sample plate and allowed to dry in air prior to analysis.

Gel permeation chromatography (GPC) was performed on a Malvern RI max Gel Permeation Chromatograph, equipped with an automatic sampler, pump, injector, and inline degasser. The columns (T5000) were contained within an oven (35 °C) and consisted of styrene / divinyl benzene gels. Sample elution was detected by means of a differential refractometer. THF (Fisher) containing 0.1 %w/w [n-Bu_4N][Br] was used as the eluent at a flow rate of 1 mL/min. Samples were dissolved in THF (2 mg/mL) and filtered with a Ministart SRP15 filter (poly(tetrafluoroethylene) membrane of 0.45 µm pore size) before analysis. The calibration was conducted using monodisperse polystyrene standards obtained from Aldrich. The lowest and highest molecular weight standards used were 2,300 Da and 994,000 Da respectively.

Differential scanning calorimetry (DSC) thermograms were measured using a Thermal Advantage DSCQ100 coupled to a RCS90 refrigerated cooling system with a heating/cooling rate of 10 °C/min. DSC samples were placed in hermetic aluminium pans for analysis.

Thermal gravimetric analysis (TGA) was carried out using a Thermal Advantage TGAQ500 with a heating rate of 10 °C/min under a nitrogen atmosphere. Samples were placed in platinum pans for analysis. DSC and TGA results were analysed using WinUA V4.5A by Thermal Advantage.

Dynamic light scattering (DLS) experiments were carried out using a Malvern Zetasizer Nano S instrument using a 5 mW He-Ne laser (λ = 632 nm) at 20 °C. Samples dissolved in THF (1 mg/mL) were filtered through a 0.45 µm into an optical glass cuvette prior to analysis.

2. Procedures for the hydrophosphination of 1-octene using poly(phenylphosphinoborane)

2.1. General NMR scale reaction conditions for Table 1

An NMR tube was charged with polyphosphinoborane (0.2 mmol), 1-octene (0.2 mmol), and solvent (0.5 mL) and any additive specified. The solution was irradiated ca. 3 cm away from a mercury lamp. After the specified amount of time, a ^{31}P NMR spectrum was recorded of the crude reaction mixture and the conversion calculated from the integration of the signals.

2.2. Attempted hydrophosphination by means of irradiation with blue light

Poly(phenylphosphinoborane) (49 mg, 0.4 mmol), 1-octene (62.8 µL, 0.4 mmol), diphenyliodonium triflate (86 mg, 0.2 mmol), 9-mesityl-10-methylacridinium perchlorate (0.8 mg, 2×10^{-3} mmol), and toluene (2 mL) were added to a Schlenk flask equipped with a magnetic stirrer. The resultant solution was irradiated ca. 3 cm away from a 3 W blue light emitting lamp for 16 h, after which an in situ ^{31}P NMR spectrum was obtained to determine reaction conversion (Table S1, entry 2).

The analogous reaction with no diphenyliodonium triflate or 9-mesityl-10-methylacridinium perchlorate present was also attempted (Table S1, entry 1). In this case no reaction was observed.

Table S1. Hydrophosphination attempts using blue light irradiation.

Entry	Additives	Temperature (°C)	Time	Conversion[a]
**1**	None	20	16 h	0
**2**	$[Ph_2I][OTf]$, photocatalyst	20	16 h	25

[a]Determined by ^{31}P NMR integrations, conversion = x / (x + y) × 100

2.3. Hydrophosphination of 1-octene by poly(phenylphosphinoborane) in the presence of AIBN

An NMR tube was charged with polyphosphinoborane (0.1 mmol), 1-octene (0.1 mmol), AIBN (0.01 mmol) and THF (0.5 mL). The solution was heated to 60 °C for 27 h and then analysed by ^{31}P NMR spectroscopy.

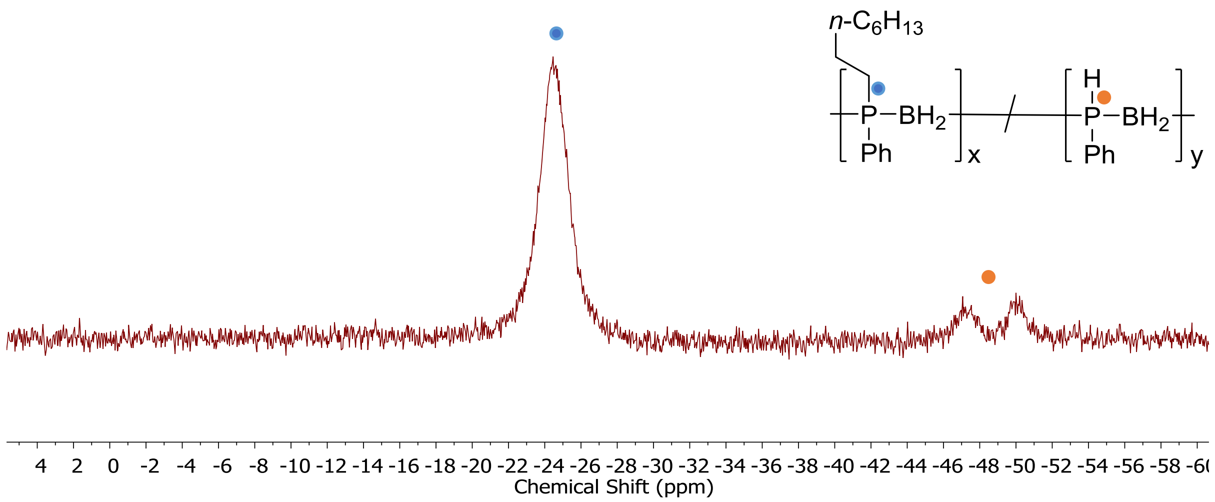

Figure S1. In situ ^{31}P NMR spectrum (122MHz) of the hydrophosphination reaction of 1-octene using poly(phenylphosphinoborane) in the presence of AIBN after 27 h.

3. Investigations into the mechanism of the hydrophosphination of 1-octene using poly(phenylphosphinoborane)

<u>3.1. Hydrophosphination of 1-octene using poly(phenylphosphinoborane) in the presence of DMPAP vs DMPAP and TEMPO</u>

Two NMR tubes were charged with polyphosphinoborane (0.2 mmol), 1-octene (0.2 mmol), DMPAP (0.02 mmol), THF (0.5 mL). To one was added TEMPO (0.02 mmol). The solutions were irradiated ca. 3 cm away from a mercury lamp for 10 min and then analysed by ^{31}P NMR spectroscopy. The samples were then irradiated for a further 50 minutes and analysed again by ^{31}P NMR.

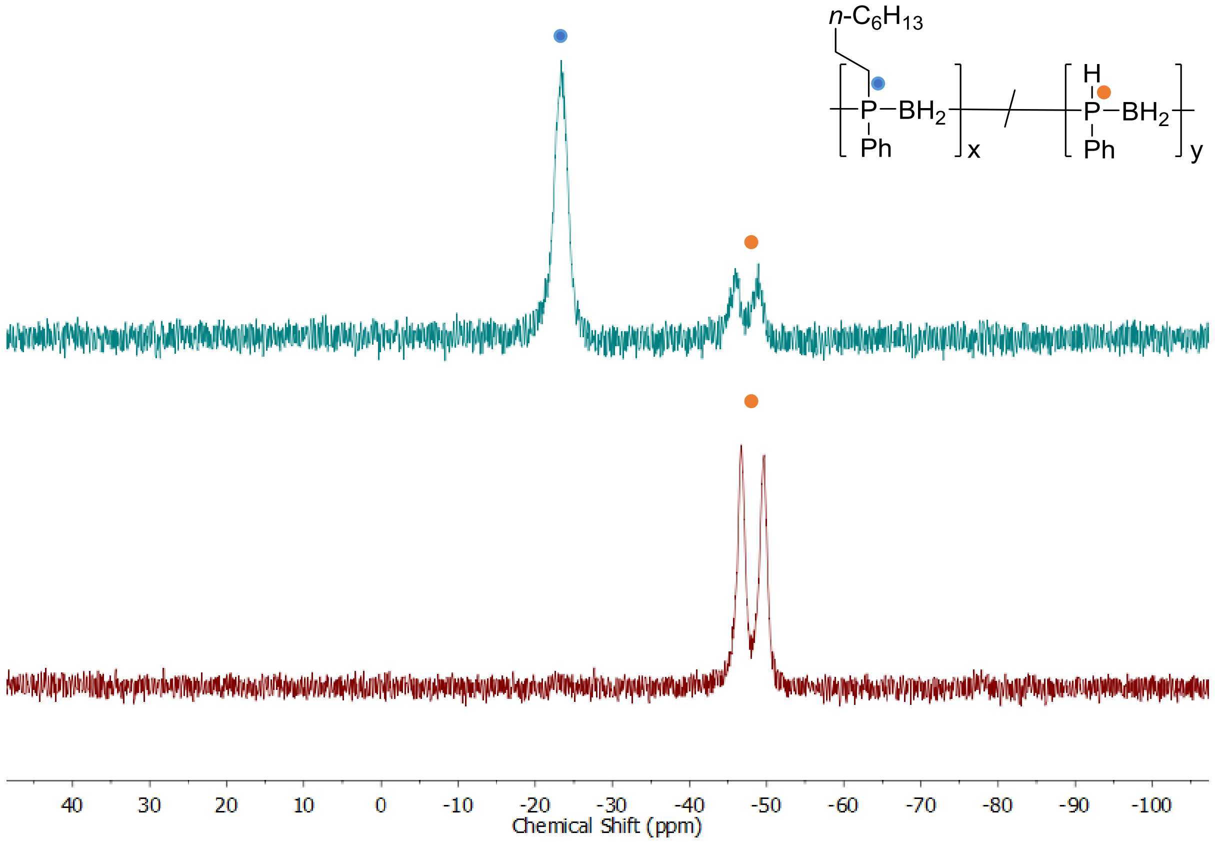

Figure S2. In situ ^{31}P NMR spectra (122MHz) taken after 10 min of the hydrophosphination of 1-octene using poly(phenylphosphinoborane) in the presence of DMPAP (top) or DMPAP and TEMPO (bottom).

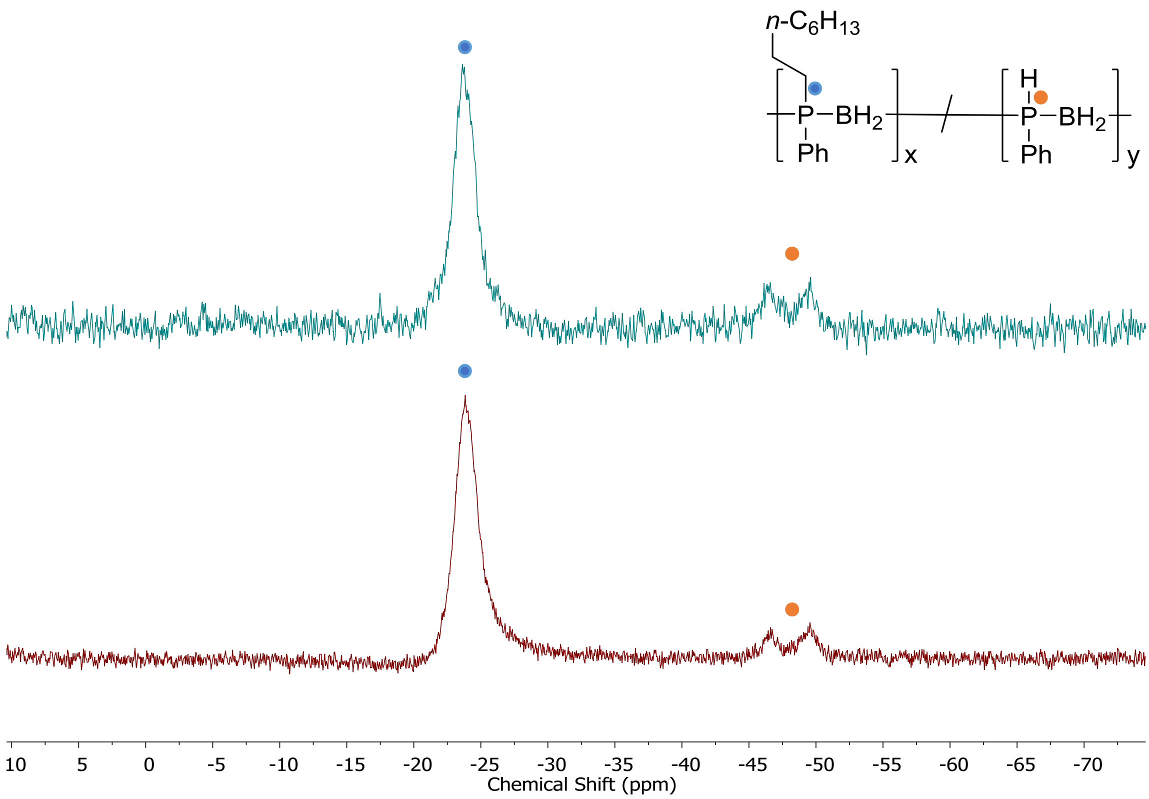

Figure S3. In situ ^{31}P NMR spectra (122MHz) taken after 1 h of the hydrophosphination of 1-octene using poly(phenylphosphinoborane) in the presence of DMPAP (top) or DMPAP and TEMPO (bottom).

3.2. Mass spectrum after the irradiation of DMPAP with TEMPO

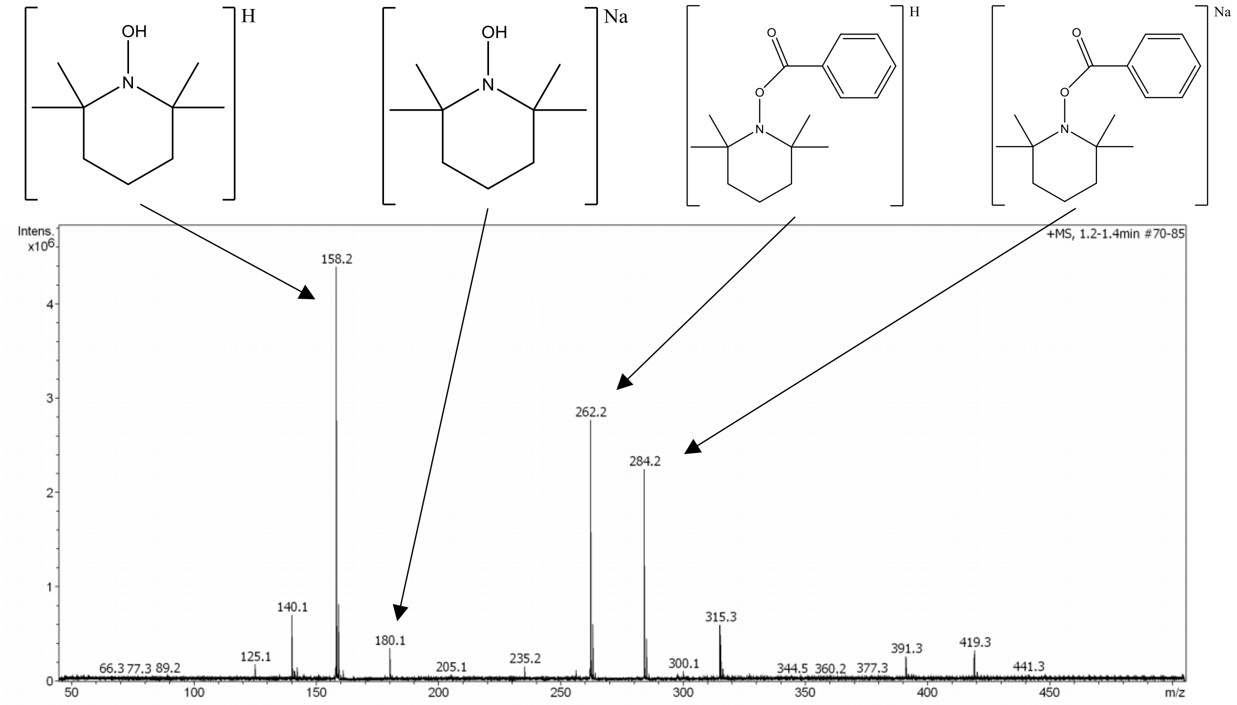

Figure S4. ESI-MS (+) of the reaction of DMPAP and TEMPO in THF.

3.3. Hydrophosphination reaction using N-tert-butyl-N-(2-methyl-1-phenylpropyl)-O-(1-phenylethyl)hydroxylamine

An NMR tube was charged with poly(phenylphosphinoborane) (0.1 mmol), 1-octene (0.1 mmol), N-tert-butyl-N-(2-methyl-1-phenylpropyl)-O-(1-phenylethyl)hydroxylamine (0.01 mmol) and toluene (0.5 mL). The solution was heated to 100 °C and monitored by ^{31}P NMR spectroscopy.

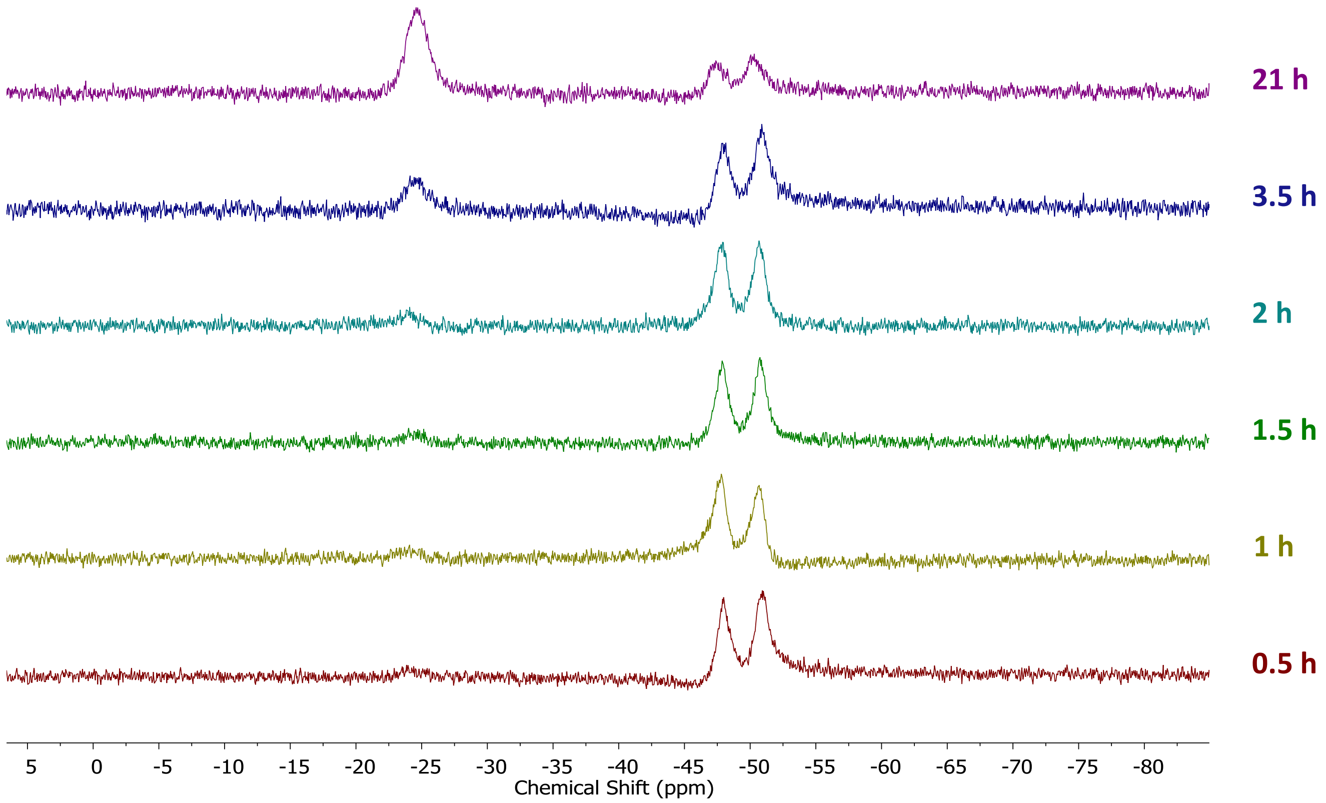

Figure S5. In situ ^{31}P NMR spectra of the hydrophosphination of 1-octene using poly(phenylphosphinoborane) in the presence of N-tert-butyl-N-(2-methyl-1-phenylpropyl)-O-(1-phenylethyl)hydroxylamine.

3.4. Irradiation of poly(phenylphosphinoborane) with DMPAP and TEMPO

To an NMR tube was added poly(phenylphosphinoborane) (0.2 mmol), DMPAP (0.2 mmol), TEMPO (0.2 mmol) and THF (0.5 mL). This was irradiated at 20 °C for 4 h and then the crude reaction mixture was analysed by ^{31}P NMR spectroscopy (Figure S6). Subsequently, 1-octene (0.2 mmol) was added and the mixture irradiated for a further 2 h and then again analysed by ^{31}P NMR (Figure S7).

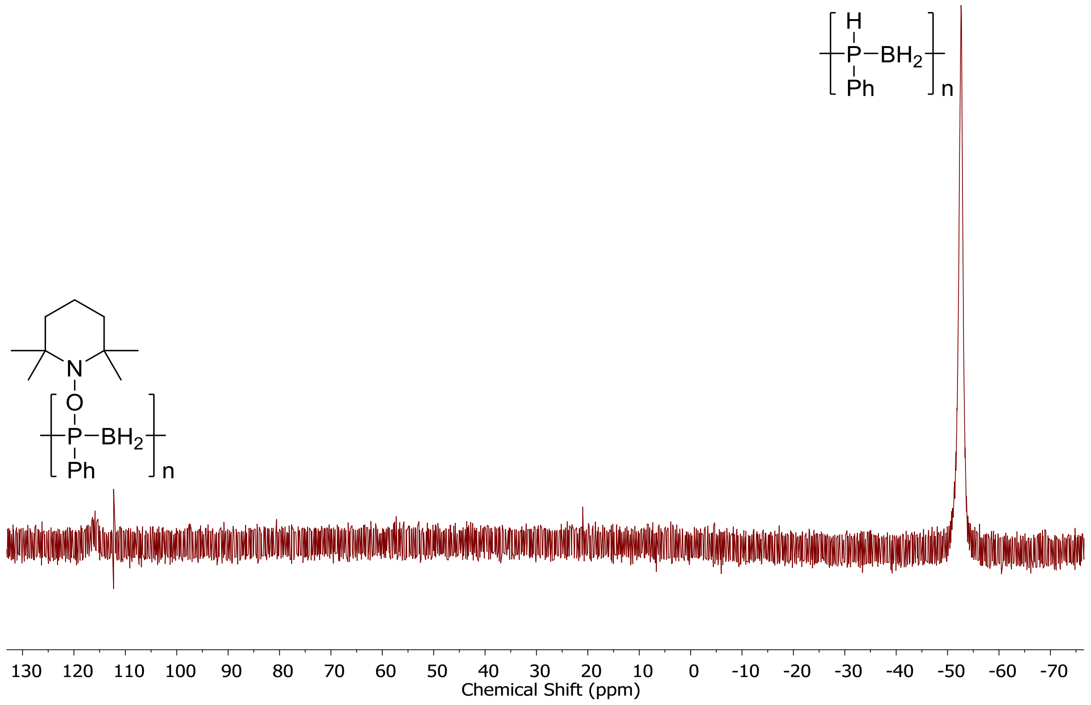

Figure S6. In situ ^{31}P{H} NMR spectra of the reaction between poly(phenylphosphinoborane), DMPAP and TEMPO after 4 h UV irradiation at 20 °C.

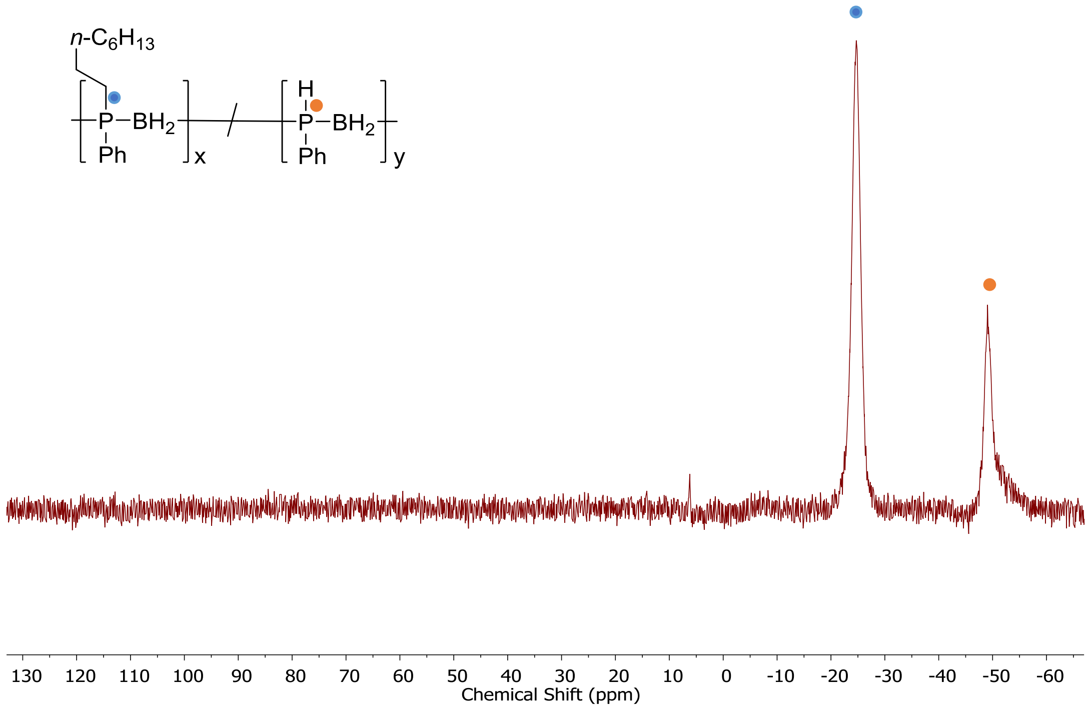

Figure S7. In situ ^{31}P{H} NMR spectra after 1 eq. 1-octene was added to the reaction mixture and it was irradiated for a further 2 h at 20 °C.

4. Synthesis and characterisation of P-disubstituted polyphosphinoboranes

4.1. General procedure for isolation of modified polyphosphinoborane derivatives:

Polyphenylphosphinoborane (2 mmol), alkene (various equivalents), (DMPAP) (0.2 mmol), TEMPO (0.2 mmol), and THF (5 mL) were added to a 14 mL vial equipped with a magnetic stirrer. The resultant solution was irradiated ca. 3 cm away from a mercury lamp. Volatiles were then removed under vacuum and the resultant solid was purified by precipitation (see specific syntheses for details). The polymer was then dried under vacuum at 40 °C for a minimum of 16 h.

4.2. Synthesis and characterisation of polymer **2**

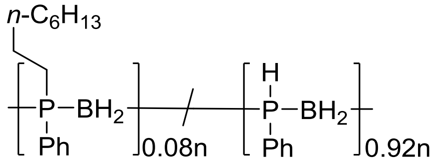

Poly(phenylphosphinoborane) (244 mg, 2 mmol), 1-octene (31.4 µL, 0.2 mmol), DMPAP (51 mg, 0.2 mmol), TEMPO (31 mg, 0.2 mmol), and THF (5 mL) were added to a 14 mL vial equipped with a magnetic stirrer. The resultant solution was irradiated ca. 3 cm away from a mercury lamp for 2 h. Volatiles were then removed under vacuum and the resultant solid was purified by precipitation from DCM into pentane at -78 °C (3 × 25 mL). The polymer was then dried under vacuum at 40 °C for 48 h yielding **2** as an orange solid (conversion: 8%; yield: 159 mg, 61%). **Spectroscopic data**: ^{1}H NMR (400 MHz, $CDCl_3$) δ 7.50 – 6.88 (br m, Ar*H*), 4.28 (br d, *J* = 360 Hz), 1.83 – 0.68 (br m, C*H*$_2$, C*H*$_3$, B*H*$_2$); ^{11}B NMR (128 MHz, $CDCl_3$) δ -34.5 (br s); ^{31}P NMR (162 MHz, $CDCl_3$) δ -23.6 (s, 8%, PhR*P*BH_2), -48.7 (d, *J* = 358 Hz, 92%, PhH*P*BH_2); GPC (2 mg/mL) M_n = 130,000 Da, PDI = 1.9; $T_{5\%}$ = 160 °C; ceramic yield = 51%; T_g = 30 °C.

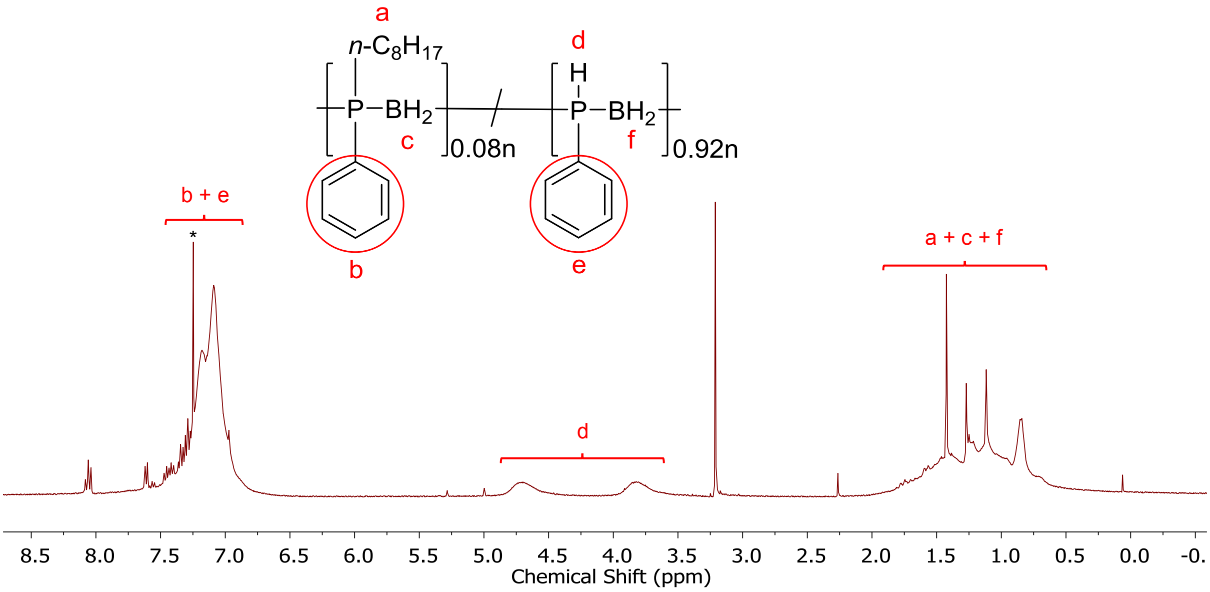

Figure S8. ^{1}H NMR spectrum (400 MHz, $CDCl_3$) of **2**. Deuterated chloroform residual signal denoted by *.

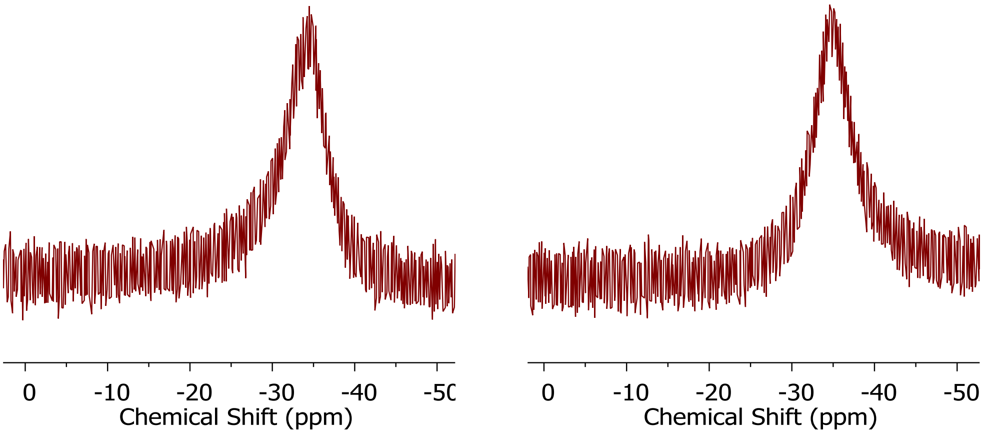

Figure S9. ^{11}B (left) and $^{11}B\{H\}$ (right) NMR spectra (128 MHz, $CDCl_3$) of **2**.

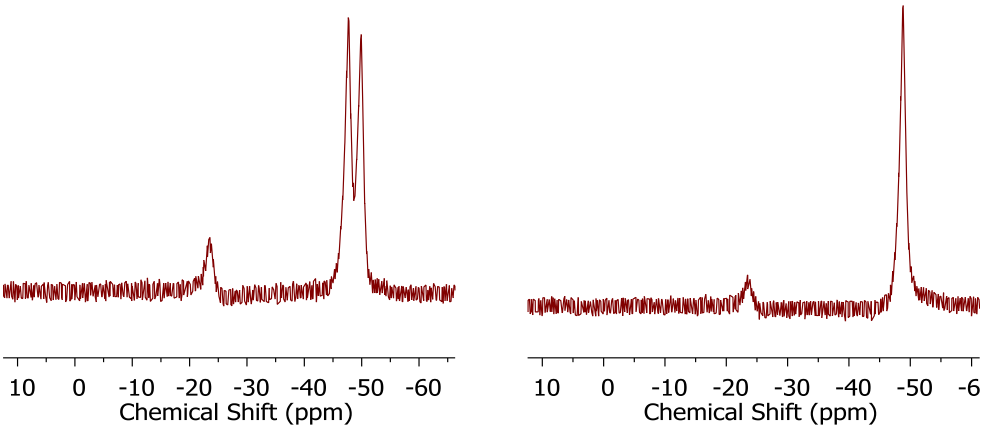

Figure S10. ^{31}P (left) and $^{31}P\{H\}$ (right) NMR spectra (162 MHz, $CDCl_3$) of **2**.

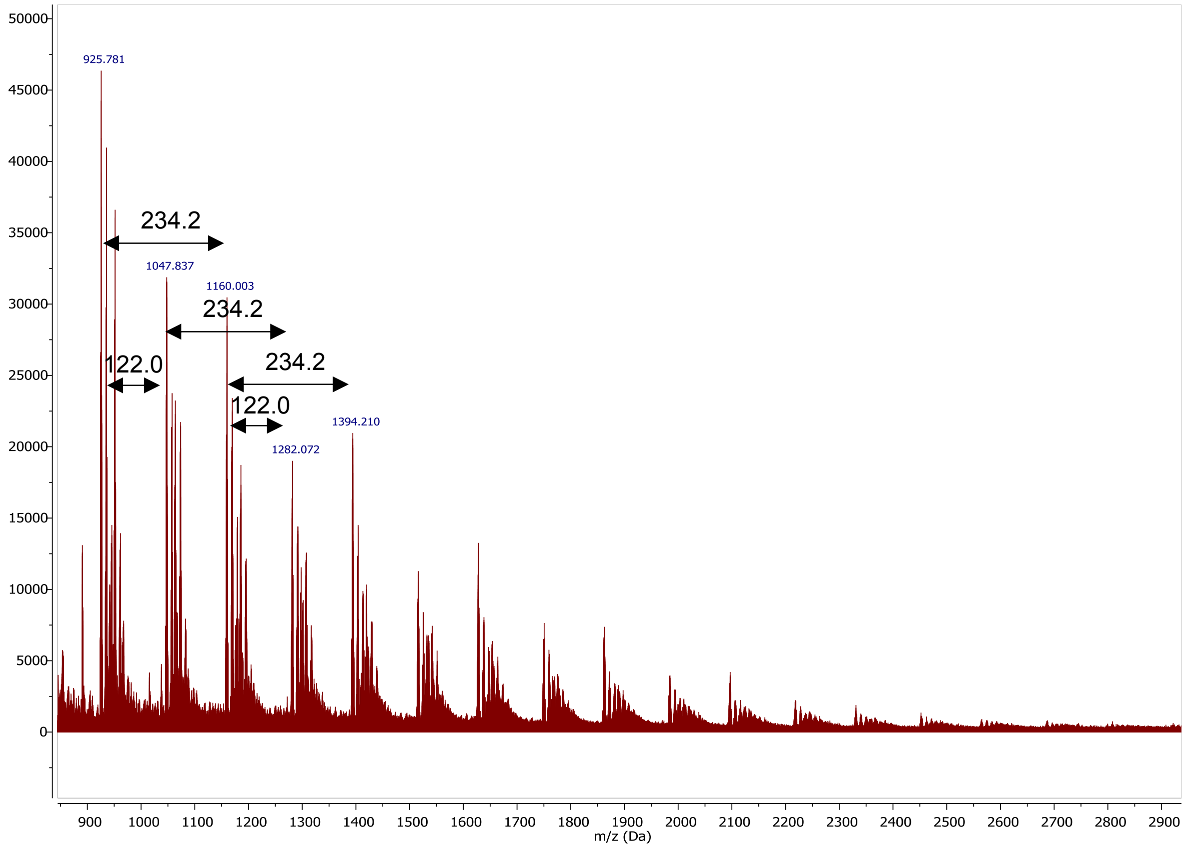

Figure S11. ESI-MS (+) of **2** in DCM.

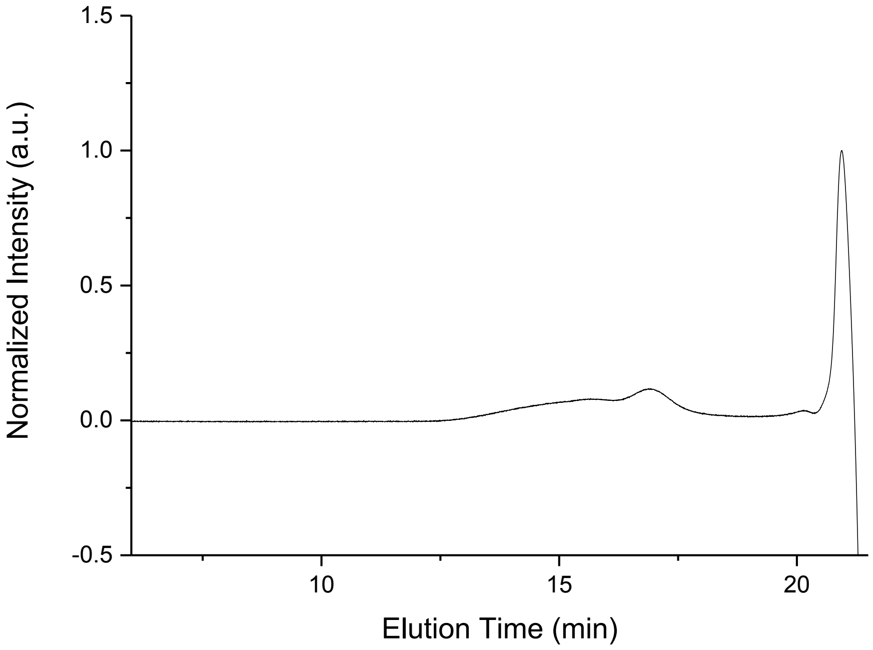

Figure S12. GPC chromatogram of **2** (2 mg/mL in THF, 0.1 w/w % *n*-Bu_4NBr in the THF eluent).

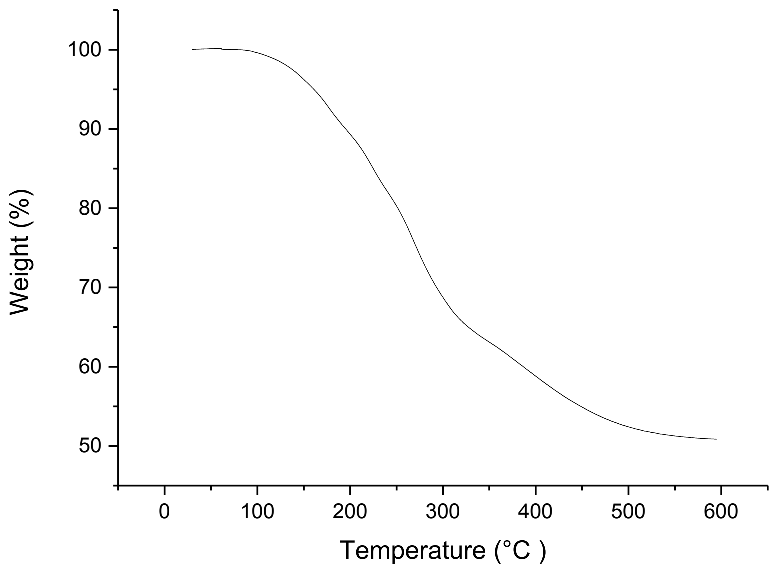

Figure S13. TGA thermogram of **2** (heating rate: 10 °C/min).

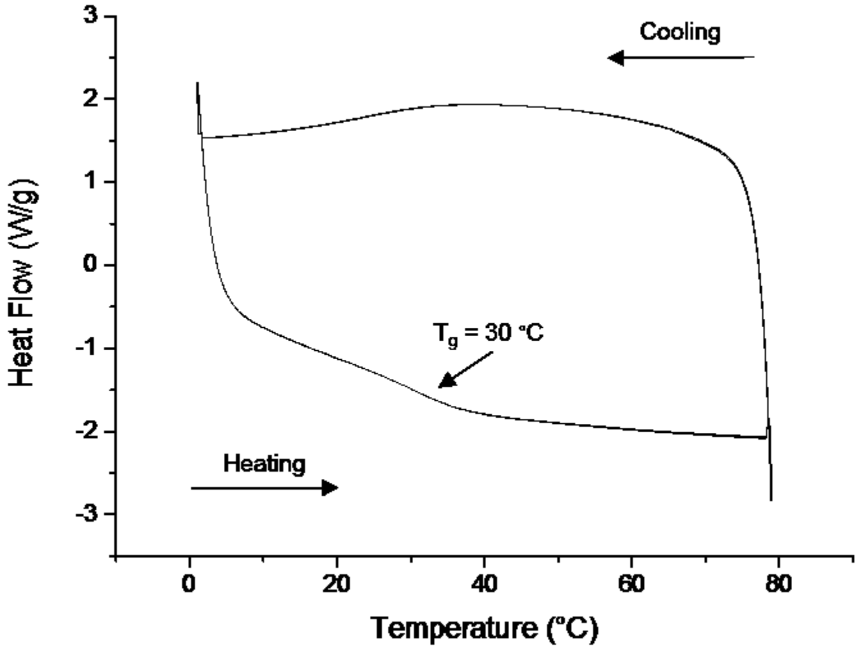

Figure S14. DSC thermogram of **2**, first cycle excluded (heating rate: 10 °C/min).

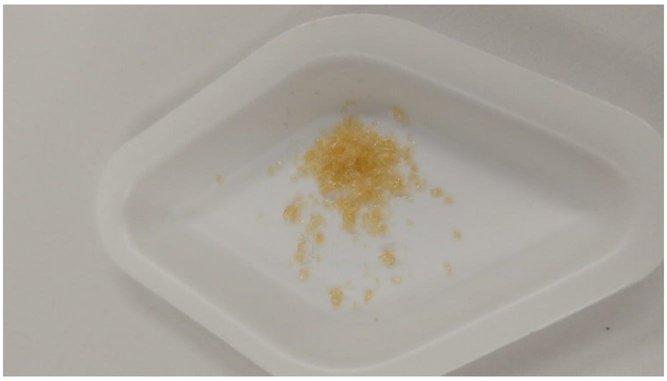

Figure S15. Photograph of isolated **2**.

4.3. Synthesis and characterisation of polymer **3**

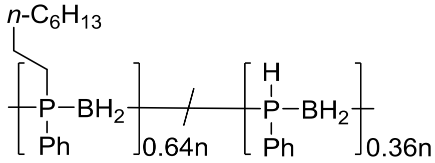

Poly(phenylphosphinoborane) (**1**) (244 mg, 2 mmol), 1-octene (157 µL, 1.2 mmol), DMPAP (51 mg, 0.2 mmol), TEMPO (31 mg, 0.2 mmol), and THF (5 mL) were added to a 14 mL vial equipped with a magnetic stirrer. The resultant solution was irradiated ca. 3 cm away from a mercury lamp for 2 h. Volatiles were then removed under vacuum and the resultant solid was purified by precipitation from THF into H_2O/isopropanol (1:1 v/v) at -20 °C (3 x 25 mL). The polymer was then dried under vacuum at 40 °C for 48 h yielding **3** as an orange solid (conversion: 64%; yield: 293 mg, 76%). **Spectroscopic data**: ^{1}H NMR (400 MHz, $CDCl_3$) δ 7.51 – 6.80 (br m, Ar*H*), 4.12 (br d, *J* = 355 Hz, PhP*H*), 1.75 – 0.58 (br m, CH_2, CH_3, BH_2); ^{11}B NMR (128 MHz, $CDCl_3$) δ -32.7 (br s); ^{31}P NMR (162 MHz, $CDCl_3$) δ -24.0 (s, 64%, PhR*P*BH_2), -48.1 (d, *J* = 359 Hz, 36%, PhH*P*BH_2); GPC (2 mg/mL) M_n = 130,000 Da, PDI = 1.5; $T_{5\%}$ = 208 °C; ceramic yield = 27%; T_g = 15 °C.

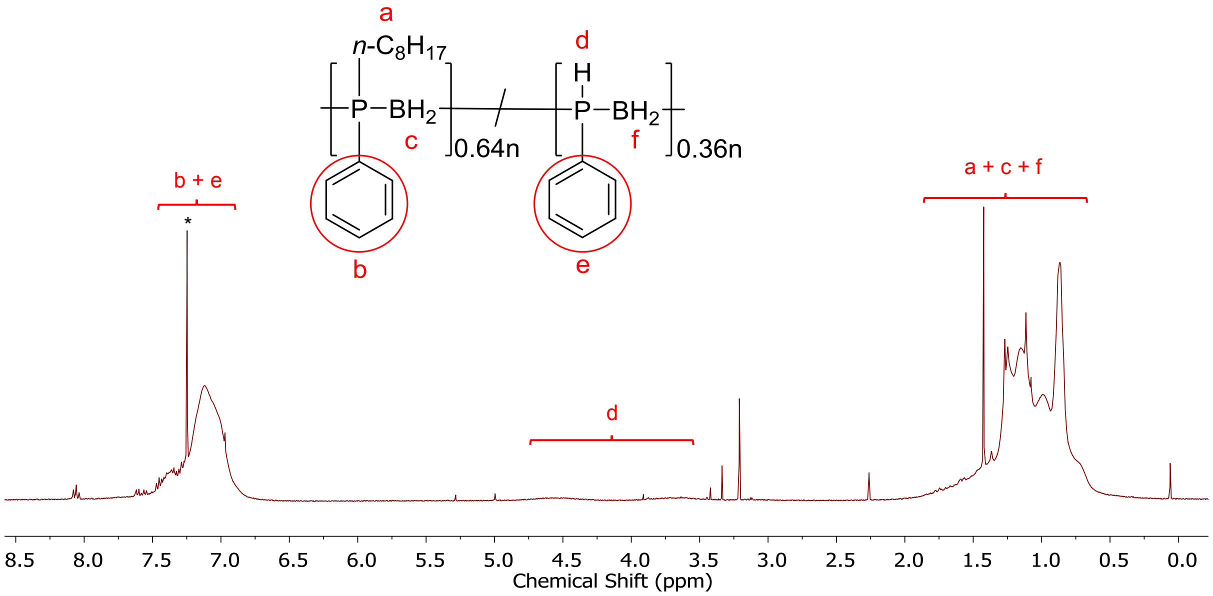

Figure S16. ^{1}H NMR spectrum (400 MHz, $CDCl_3$) of **3**. Deuterated chloroform residual signal denoted by *

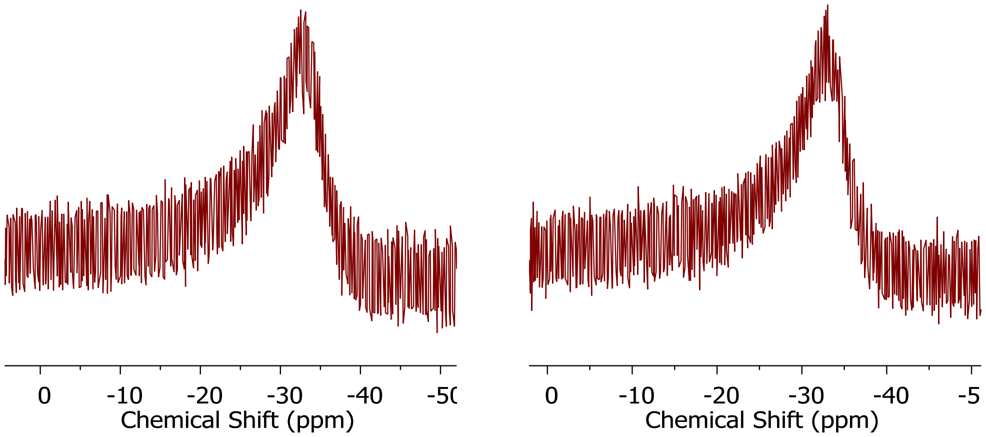

Figure S17. ^{11}B (left) and ^{11}B{H} (right) NMR spectra (128 MHz, $CDCl_3$) of **3**.

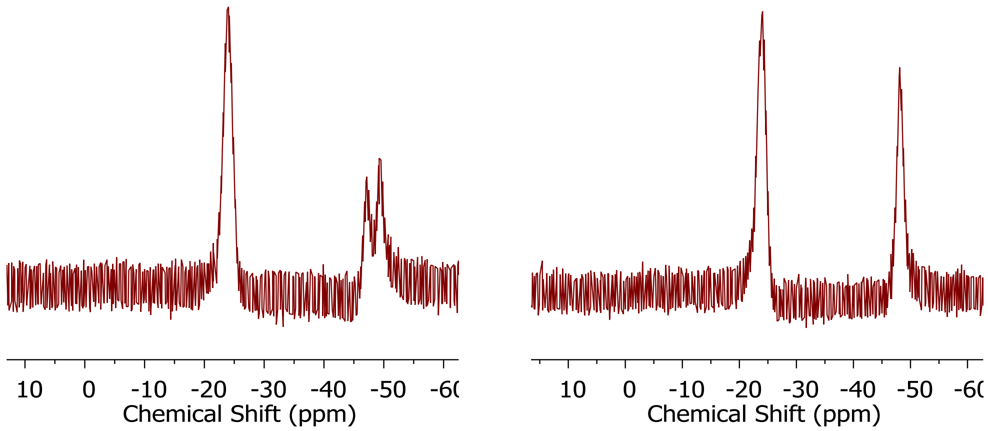

Figure S18. ^{31}P (left) and $^{31}P\{H\}$ (right) NMR spectra (162 MHz, $CDCl_3$) of **3**.

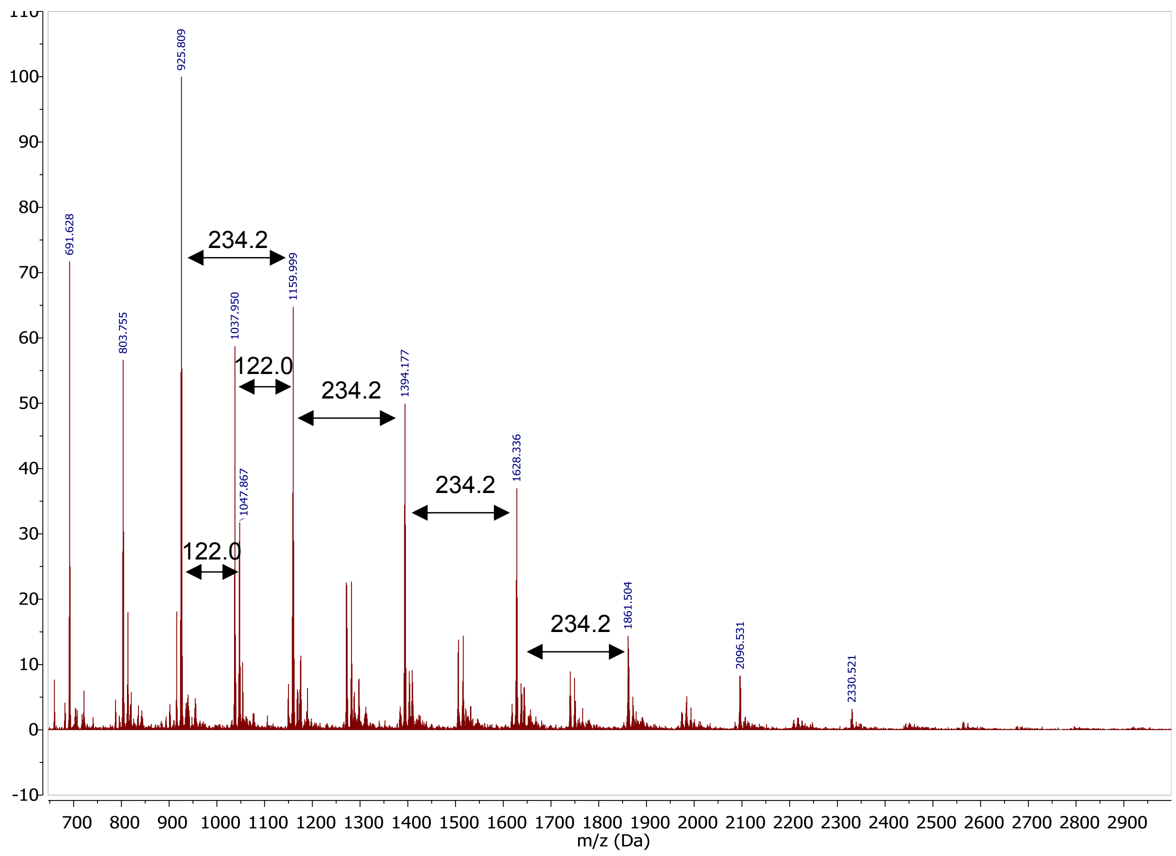

Figure S19. ESI-MS (+) spectrum of **3** in DCM.

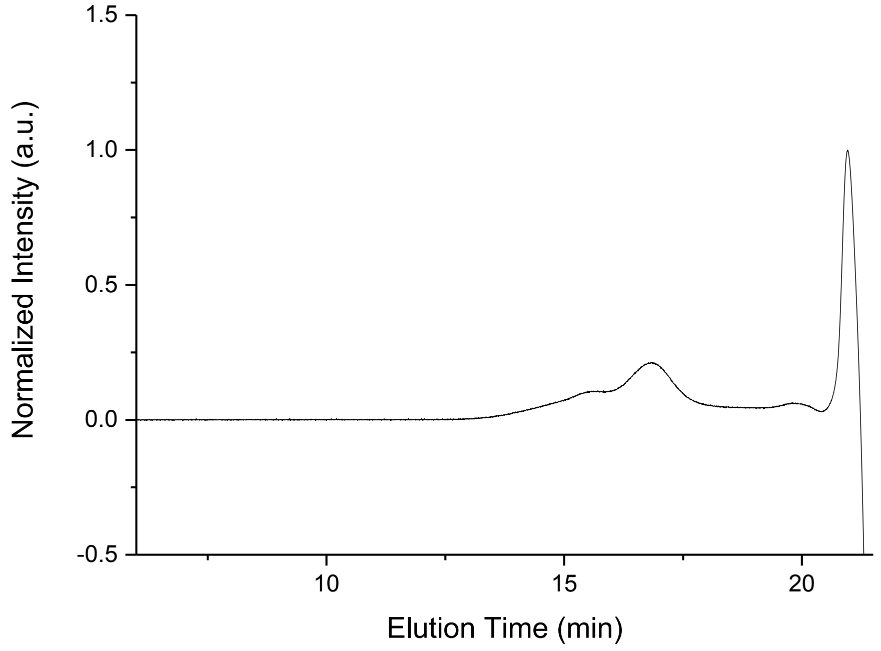

Figure S20. GPC chromatogram of **3** (2 mg/mL in THF, 0.1 w/w % *n*-Bu_4NBr in the THF eluent).

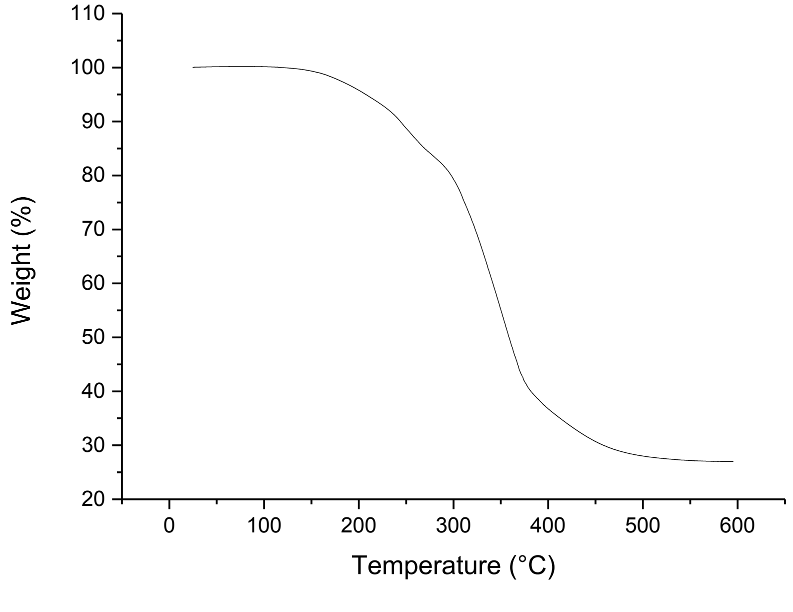

Figure S21. TGA thermogram of **3** (heating rate: 10 °C/min).

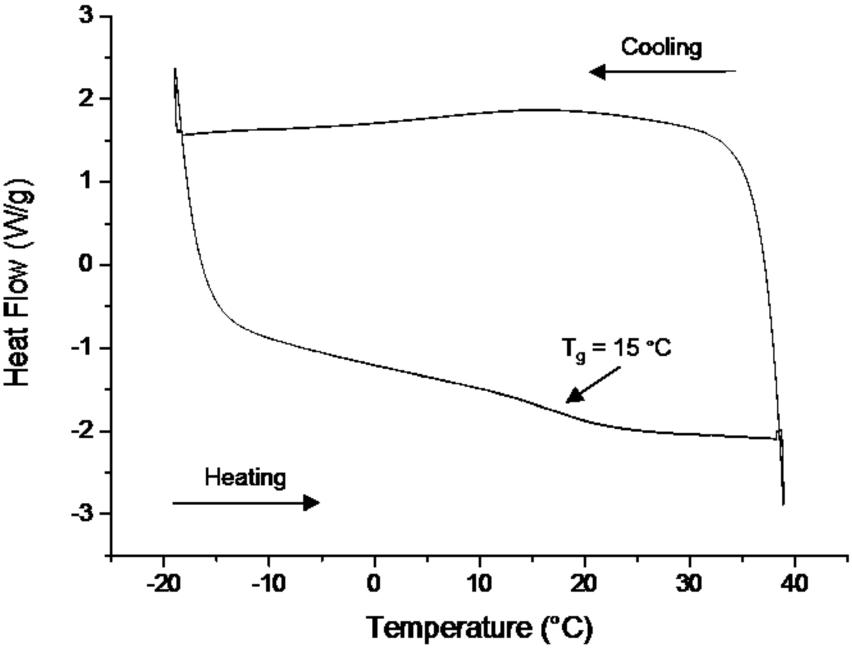

Figure S22. DSC thermogram of **3**, first cycle excluded (heating rate: 10 °C/min).

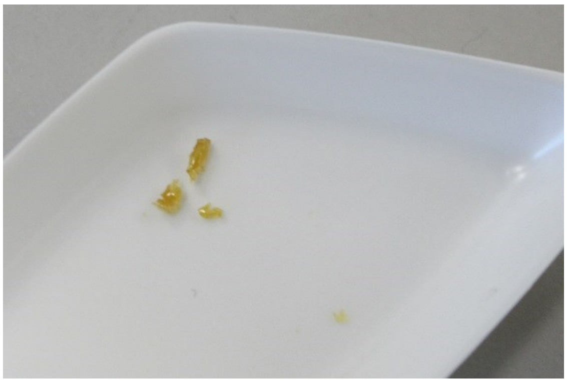

Figure S23. Photograph of isolated **3**.

4.4. Synthesis and characterisation of polymer **4**

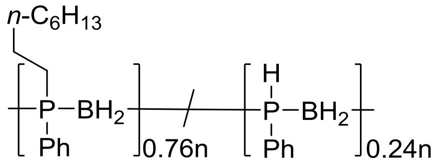

Poly(phenylphosphinoborane) (244 mg, 2 mmol), 1-octene (159 μL, 2 mmol), DMPAP (51 mg, 0.2 mmol), TEMPO (31 mg, 0.2 mmol), and THF (5 mL) were added to a 14 mL vial equipped with a magnetic stirrer. The resultant solution was irradiated ca. 3 cm away from a mercury lamp for 2 h. Volatiles were then removed under vacuum and the resultant solid was purified

by precipitation from THF into H_2O/isopropanol (1:1 v/v) at -20 °C (3 x 25 mL). The polymer was then dried under vacuum at 40 °C for 48 h yielding **4** as a gummy orange solid (conversion: 76%; yield: 305 mg, 72 %). **Spectroscopic data**: ^{1}H NMR (400 MHz, $CDCl_3$) δ 7.64 – 6.89 (br m, Ar*H*), 4.10 (br d, J = 343 Hz, PhP*H*), 2.02 – 0.59 (br m, CH_2, CH_3, BH_2); ^{11}B NMR (128 MHz, $CDCl_3$) δ -32.5 (br s); ^{31}P NMR (162 MHz, $CDCl_3$) δ -24.0 (s, 76%, PhR*P*BH_2), -48.2 (d, *J* = 356.0 Hz, 24%, PhH*P*BH_2); GPC (2 mg/mL) M_n = 81,000 Da, PDI = 1.2 $T_{5\%}$ = 197 °C; ceramic yield = 20%; T_g = 9 °C.

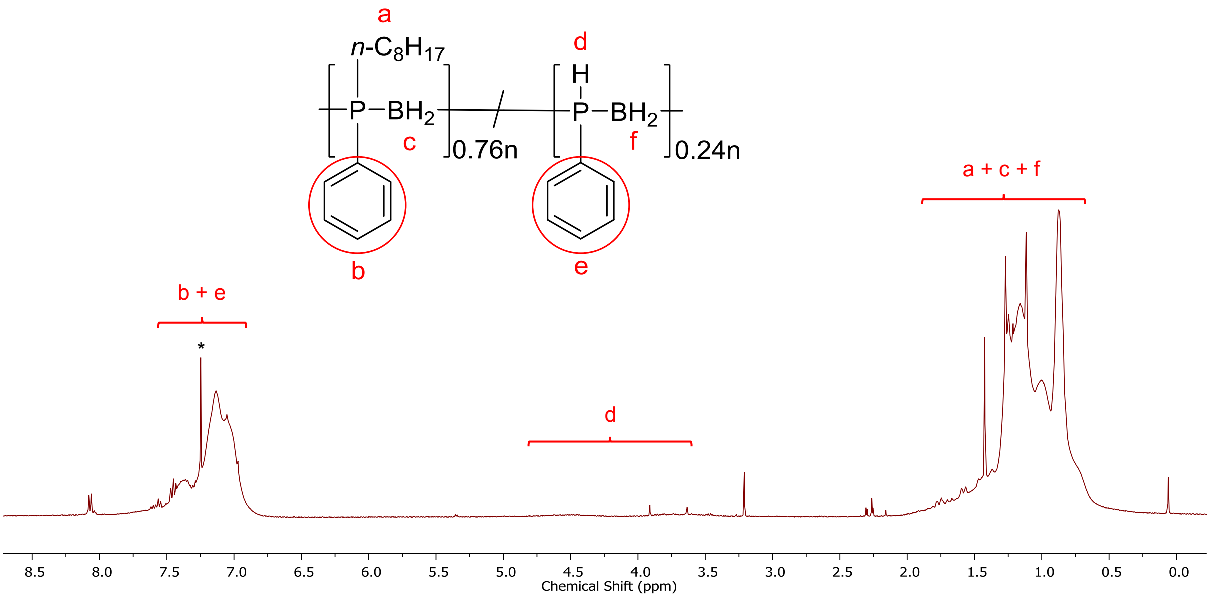

Figure S24. ^{1}H NMR spectrum (400 MHz, $CDCl_3$) of **4**. Deuterated chloroform residual signal denoted by *.

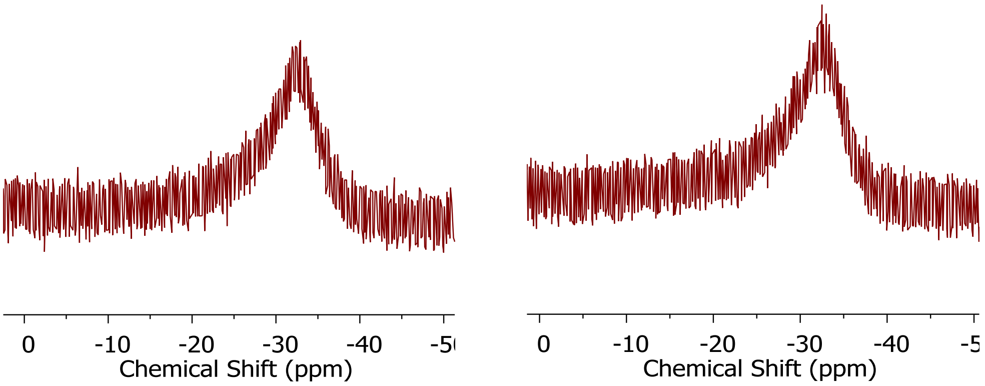

Figure S25. ^{11}B (left) and $^{11}B\{H\}$ (right) NMR spectra (128 MHz, $CDCl_3$) of **4**.

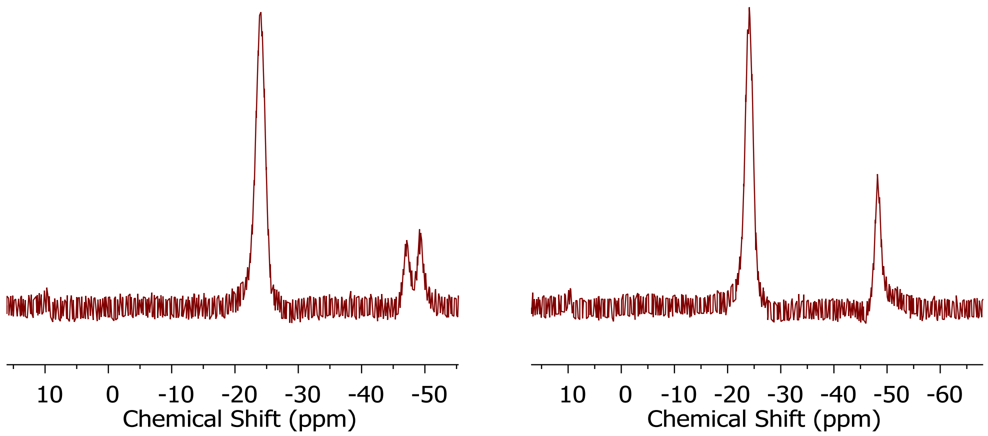

Figure S26. ^{31}P (left) and $^{31}P\{H\}$ (right) NMR spectra (162 MHz, $CDCl_3$) of **4**.

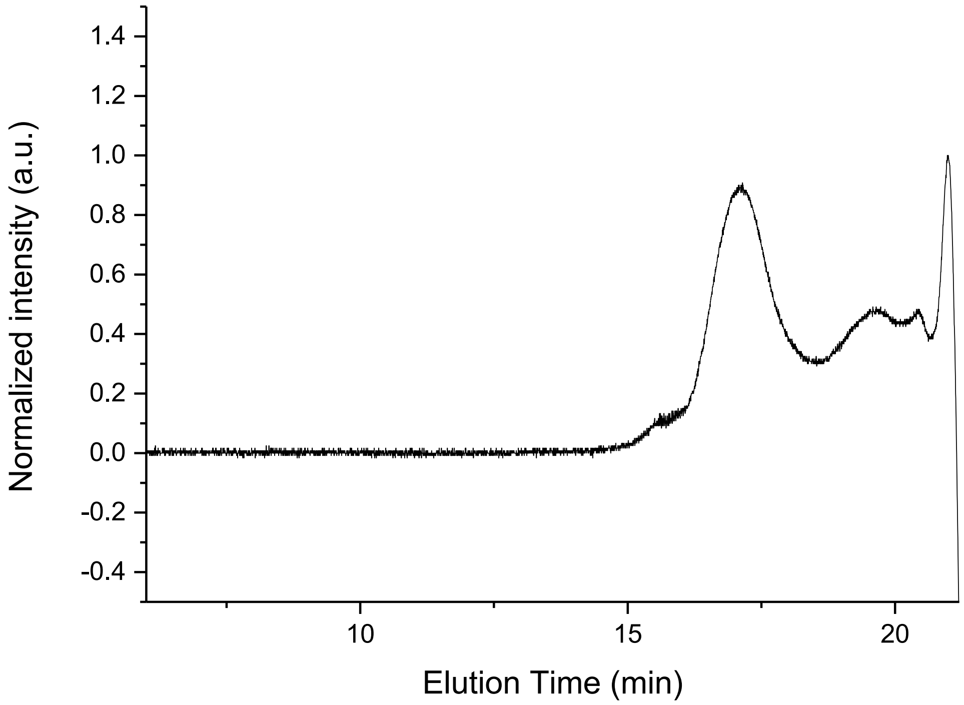

Figure S27. GPC chromatogram of **4** (2 mg/mL in THF, 0.1 w/w % *n*-Bu_4NBr in the THF eluent).

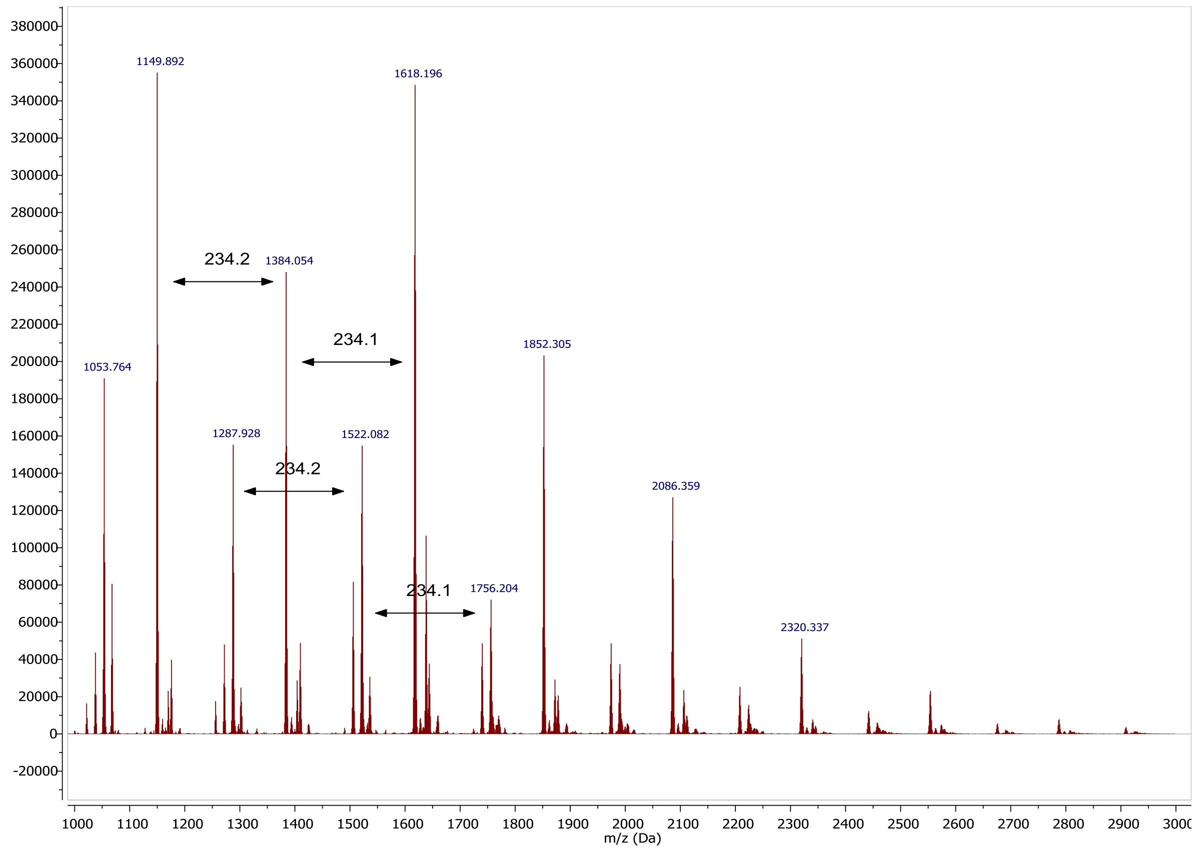

Figure S28. ESI-MS (+) spectrum of **4** in DCM.

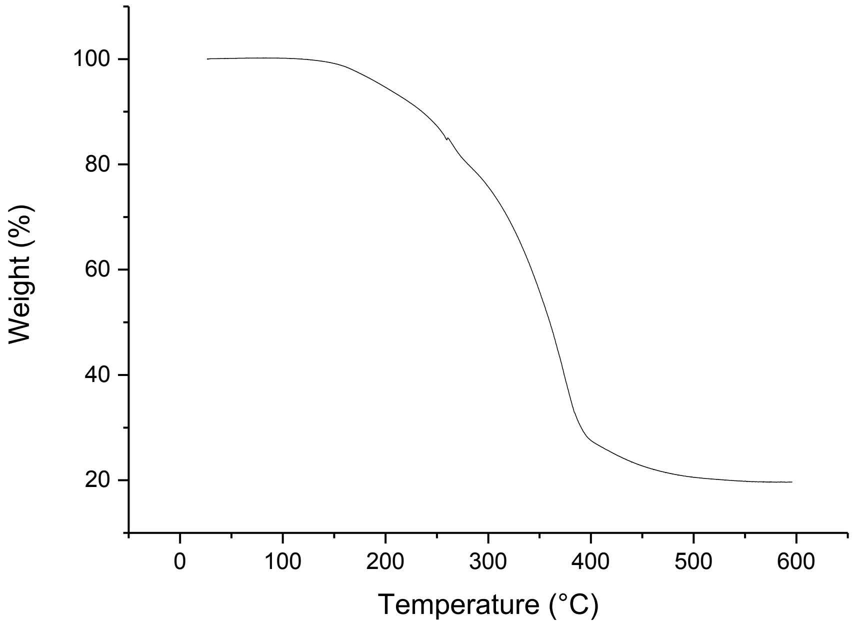

Figure S29. TGA thermogram of **4** (heating rate: 10 °C/min).

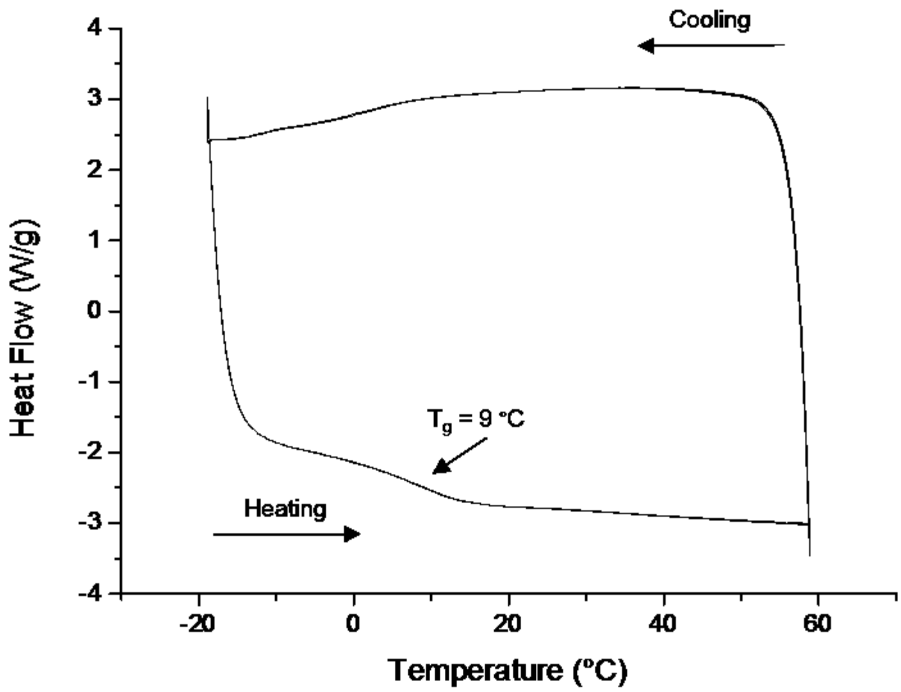

Figure S30. DSC thermogram of **4**, first cycle excluded (heating rate: 10 °C/min).

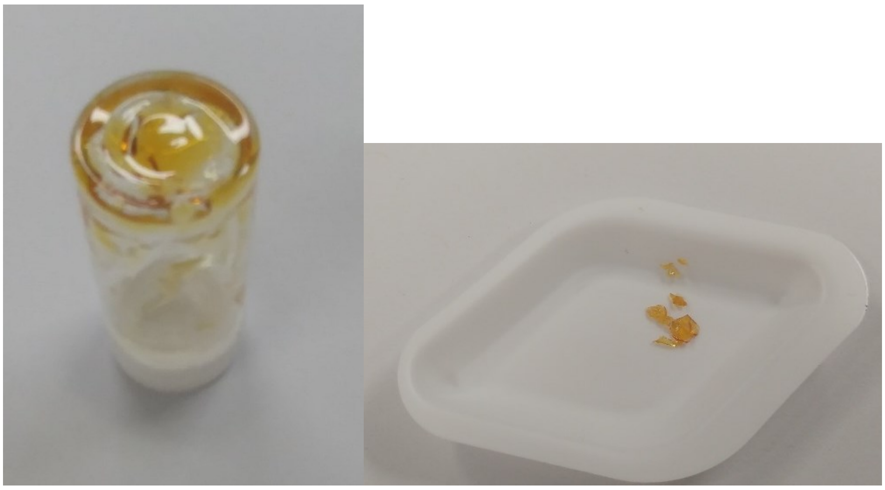

Figure S31. Photographs of isolated **4**.

<u>4.5. Synthesis and characterisation of polymer **5**</u>

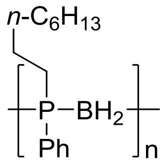

Poly(phenylphosphinoborane) (244 mg, 2 mmol), 1-octene (159 µL, 4 mmol), DMPAP (51 mg, 0.2 mmol), TEMPO (31 mg, 0.2 mmol), and THF (5 mL) were added to a 14 mL vial equipped with a magnetic stirrer. The resultant solution was irradiated ca. 3 cm away from a mercury lamp for 24 h. Volatiles were then removed under vacuum and the resultant solid was purified by precipitation from THF into H_2O/isopropanol (1:1 v/v) at -20 °C (3 x 25 mL). The polymer was then dried under vacuum at 40 °C for 48 h yielding **4** as a gummy orange solid (conversion: 100%; yield: 297 mg, 63 %). **Spectroscopic data**: ^{1}H NMR (400 MHz, $CDCl_3$) δ 7.64 – 6.89 (br m, Ar*H*), 2.02 – 0.59 (br m, C*H*$_2$, C*H*$_3$, B*H*$_2$); ^{11}B NMR (128 MHz, $CDCl_3$) δ -32.5 (br s); ^{31}P NMR (162 MHz, $CDCl_3$) δ -24.0 (s, 76%, PhR*P*BH$_2$); GPC (2 mg/mL) M_n = 112,000 Da, PDI = 1.1; $T_{5\%}$ = 216 °C; ceramic yield = 6%; T_g = 4 °C.

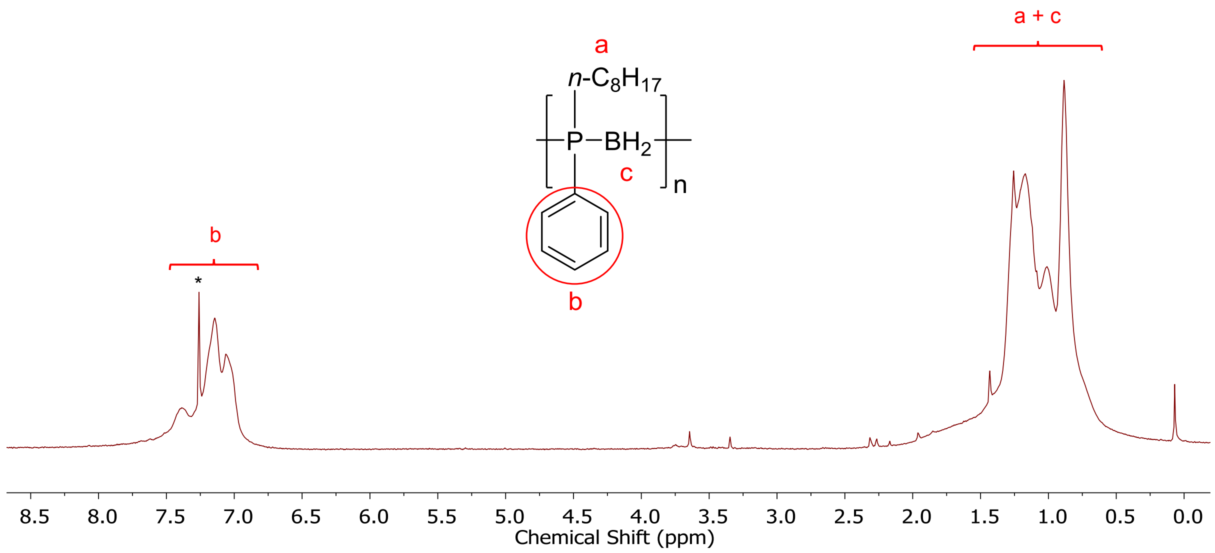

Figure S32. ^{1}H NMR spectrum (400 MHz, $CDCl_3$) of **5**. Deuterated chloroform residual signal denoted by *.

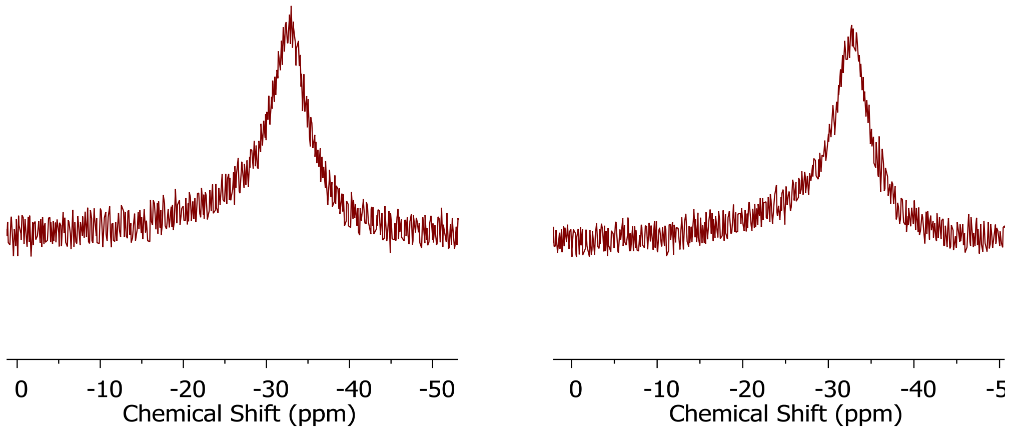

Figure S33. ^{11}B (left) and $^{11}B\{H\}$ (right) NMR spectra (128 MHz, $CDCl_3$) of **5**.

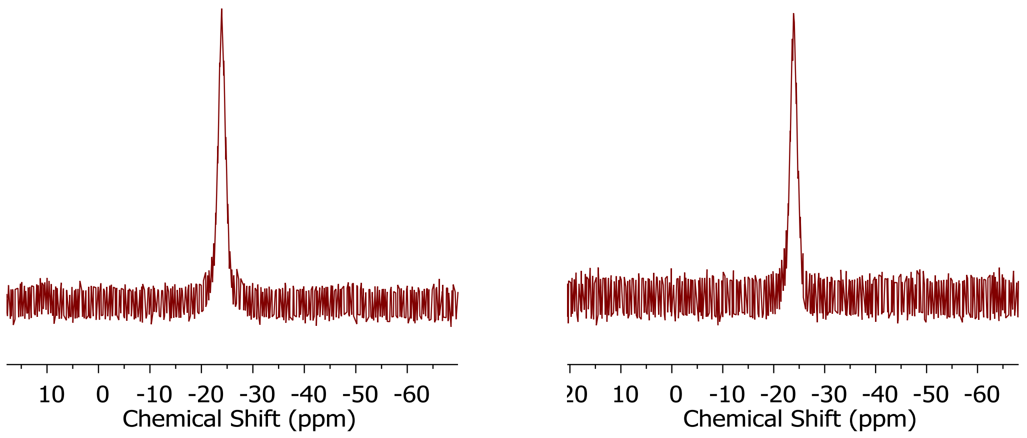

Figure S34. ^{31}P (left) and $^{31}P\{H\}$ (right) NMR spectra (162 MHz, $CDCl_3$) of **5**.

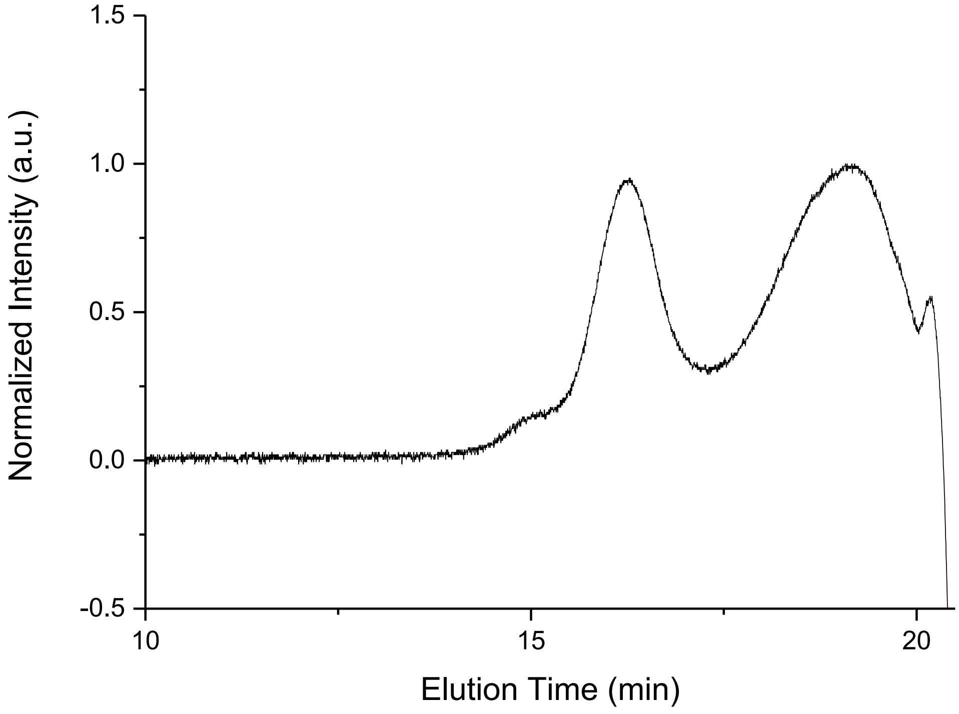

Figure S35. GPC chromatogram of **5** (2 mg/mL in THF, 0.1 w/w % *n*-Bu_4NBr in the THF eluent).

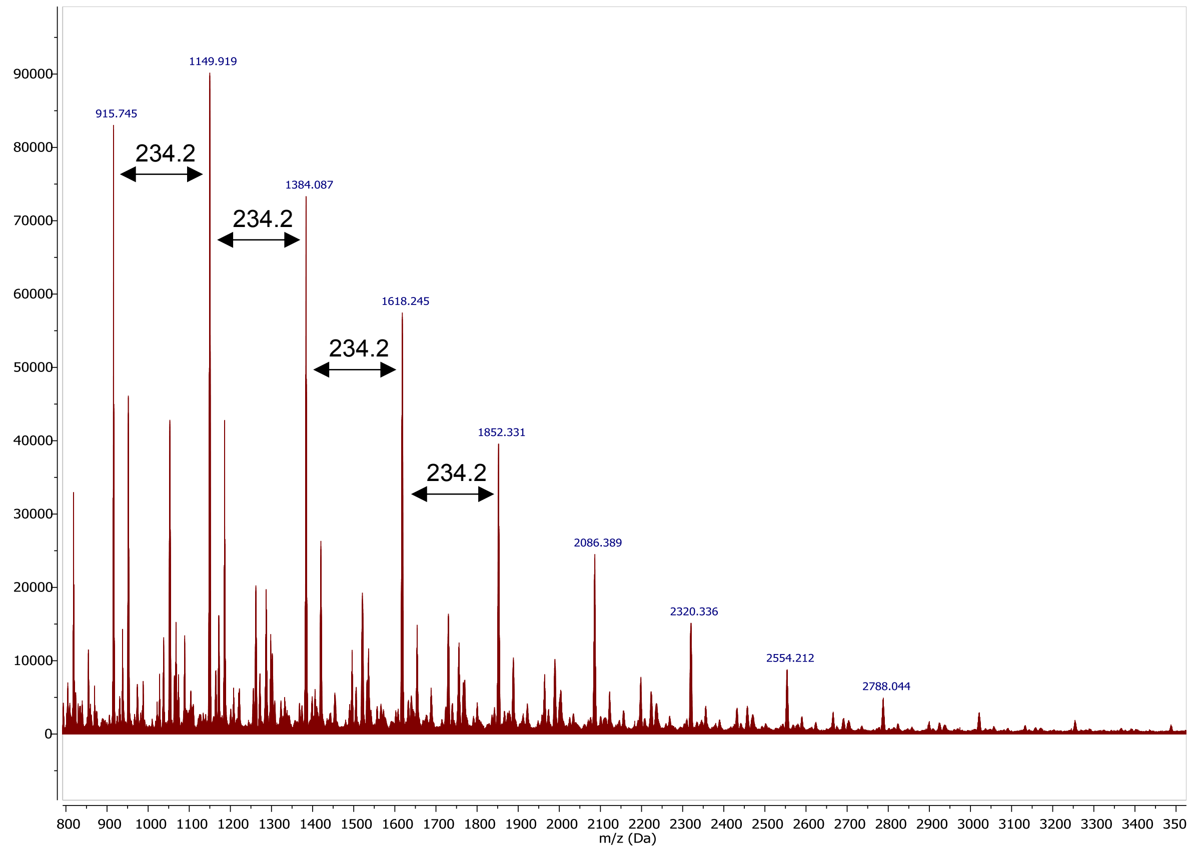

Figure S36. ESI-MS (+) spectrum of **5** in DCM.

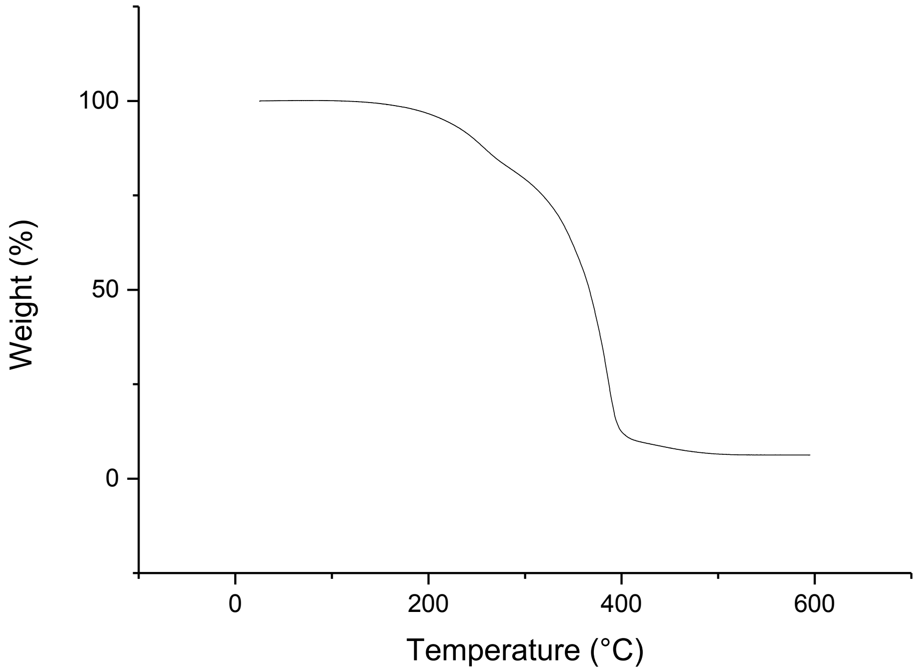

Figure S37. TGA thermogram of **5** (heating rate: 10 °C/min).

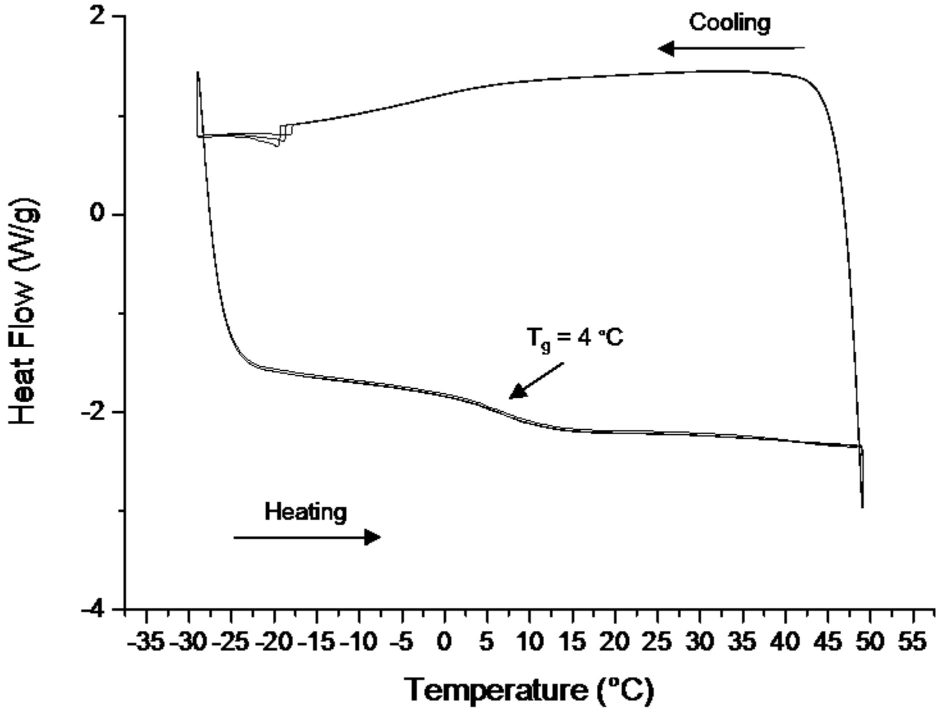

Figure S38. DSC thermogram of **5**, first cycle excluded (heating rate: 10 °C/min).

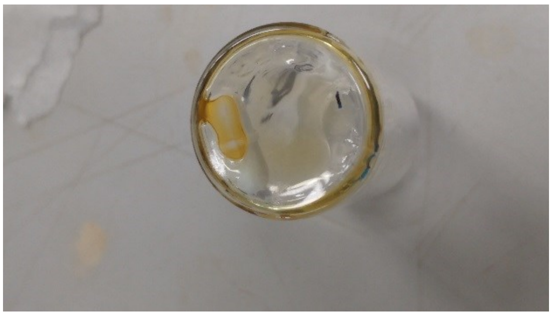

Figure S39. Photograph of isolated **5**.

4.6. Synthesis and characterisation of polymer **6**

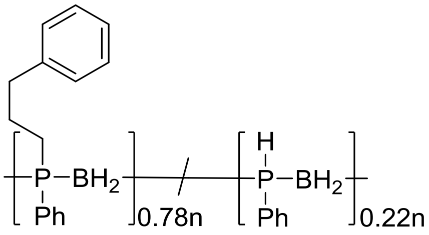

Poly(phenylphosphinoborane) (244 mg, 2 mmol), allylbenzene (265 µL, 2 mmol), DMPAP (51 mg, 0.2 mmol), TEMPO (31 mg, 0.2 mmol), and THF (5 mL) were added to a 14 mL vial

equipped with a magnetic stirrer. The resultant solution was irradiated ca. 3 cm away from a mercury lamp for 2 h. Volatiles were then removed under vacuum and the resultant solid was purified by precipitation from DCM into pentane at -78 °C (3 x 25 mL). The polymer was then dried under vacuum at 40 °C for 48 h yielding **6** as an orange solid (conversion: 78%; yield: 249 mg, 57%). **Spectroscopic data:** ^{1}H NMR (400 MHz, $CDCl_3$) δ 7.48 – 6.80 (m, Ar*H*), 4.10 (br d, *J* = 349 Hz, PhP*H*), 2.10 (br s, PhC*H*$_2$), 1.79 – 0.80 (br m, PC*H*$_2$, PCH_2C*H*$_2$, and B*H*$_2$); ^{11}B NMR (128 MHz, $CDCl_3$) δ -33.4 (br s); ^{31}P NMR (162 MHz, $CDCl_3$) δ -23.9 (s, 78%, PhR*P*BH_2), -48.5 (d, *J* = 354 Hz, 22%, PhH*P*BH_2); M_n = 104,000 Da, PDI = 1.3; $T_{5\%}$ = 194 °C; ceramic yield = 19%; T_g = 50 °C.

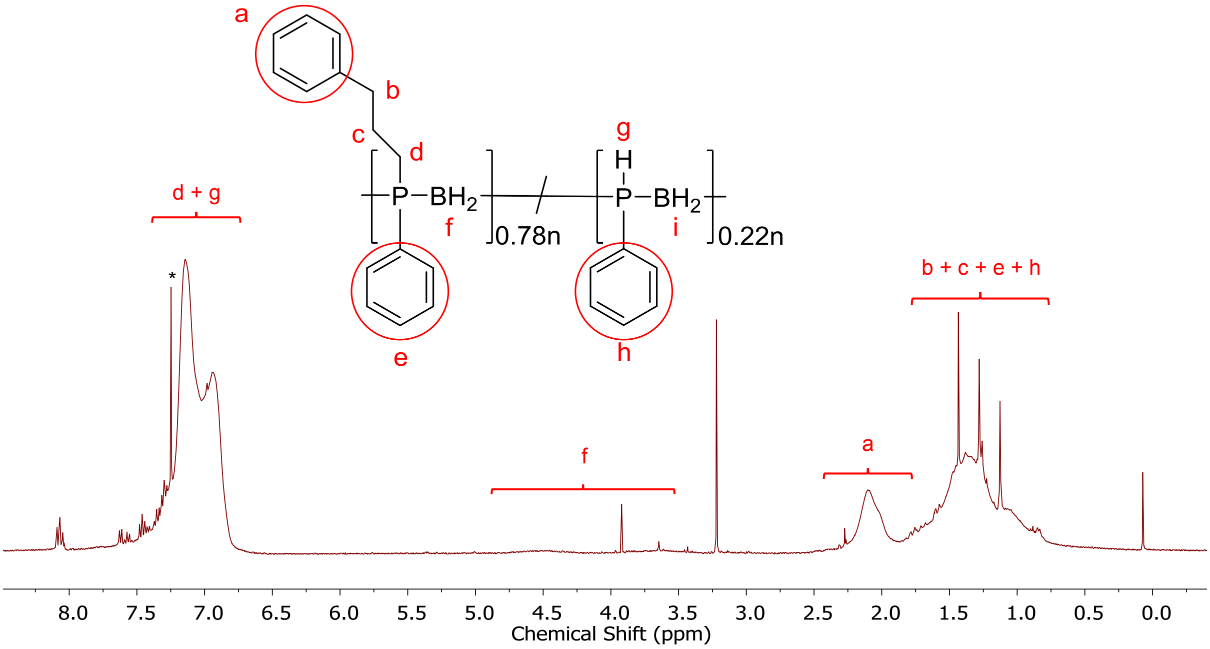

Figure S40. ^{1}H NMR spectrum (400 MHz, $CDCl_3$) of **6**. Deuterated chloroform residual signal denoted by *.

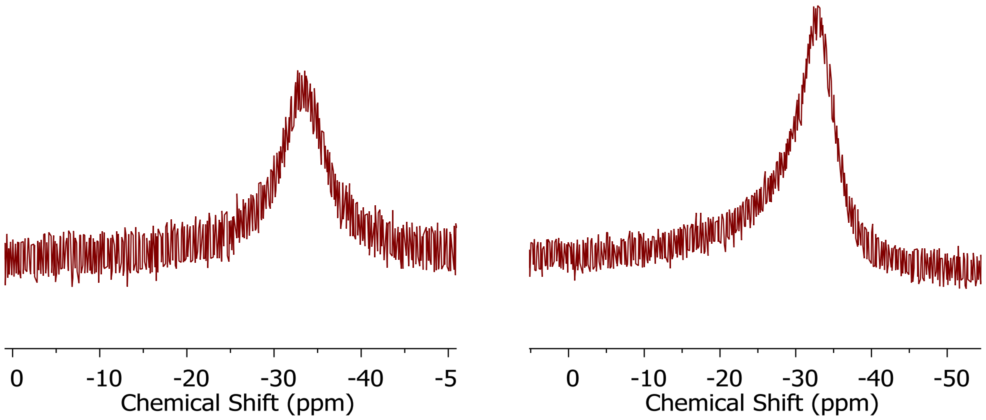

Figure S41. ^{11}B (left) and ^{11}B{H} (right) NMR spectra (128 MHz, $CDCl_3$) of **6**.

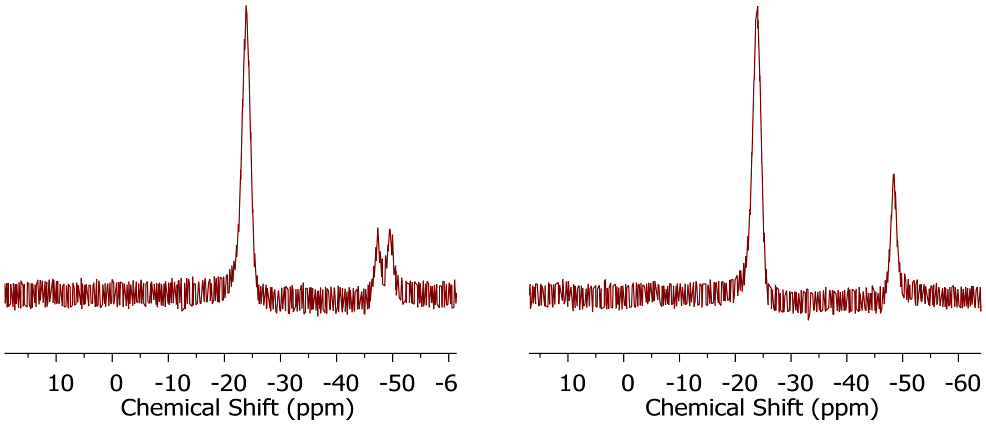

Figure S42. ^{31}P NMR (left) and ^{31}P{H} (162 MHz, $CDCl_3$) of **6**.

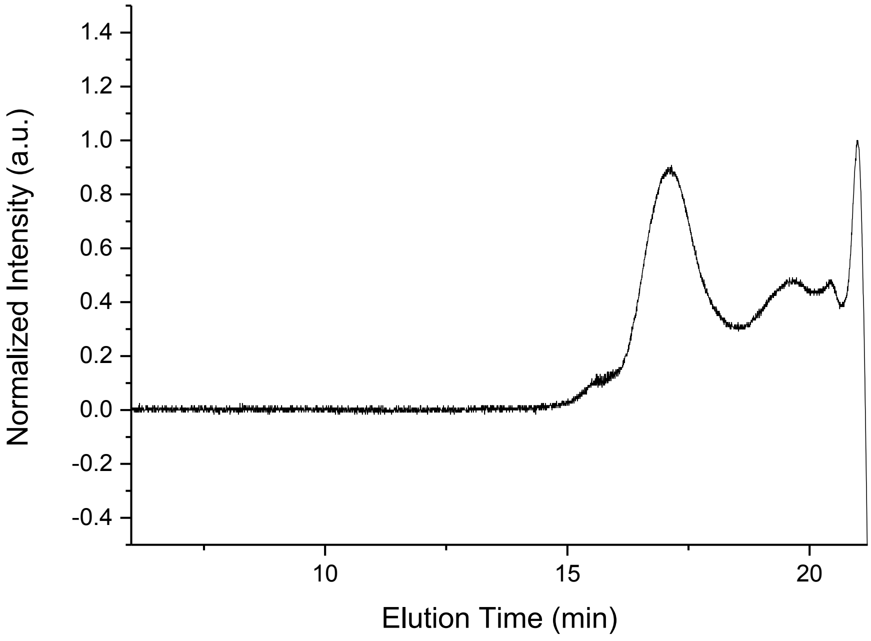

Figure S43. GPC chromatogram of **6** (2 mg/mL in THF, 0.1 w/w % *n*-Bu_4NBr in the THF eluent).

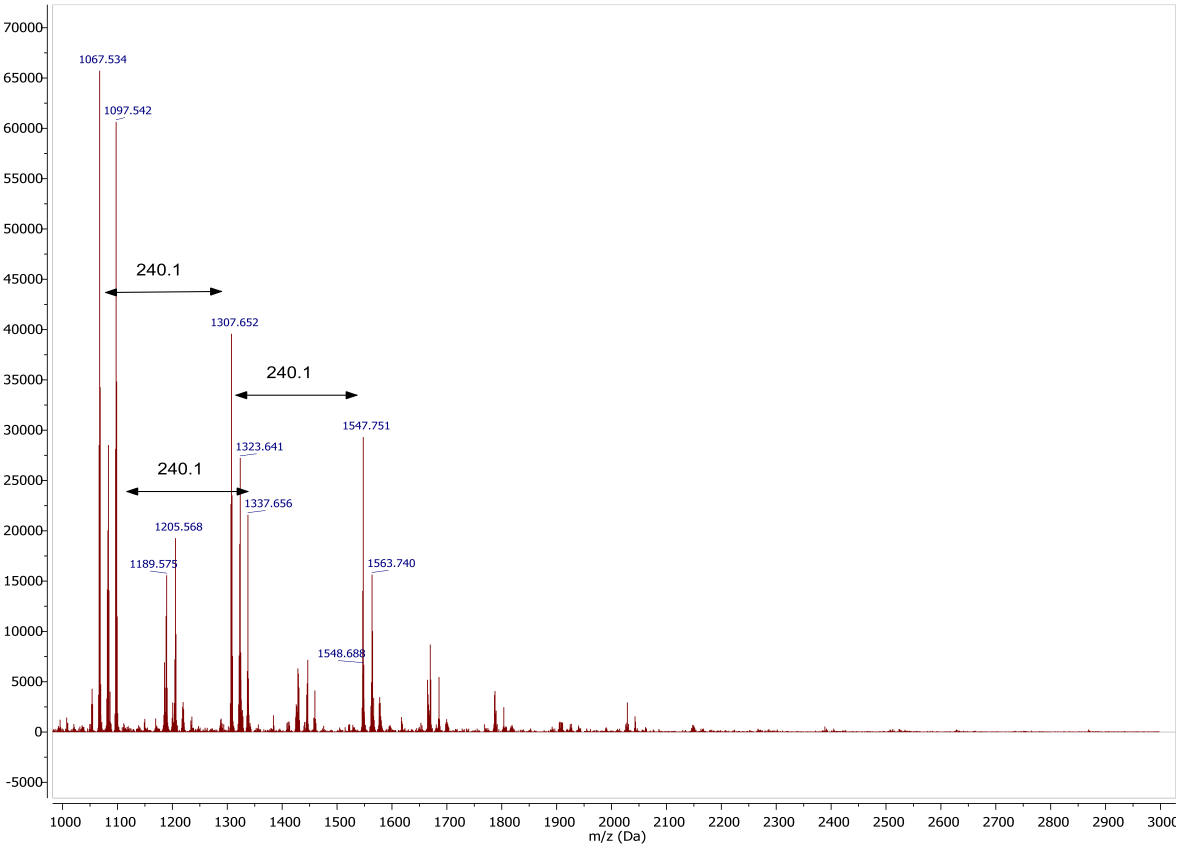

Figure S44. ESI-MS (+) spectrum of **6** in DCM.

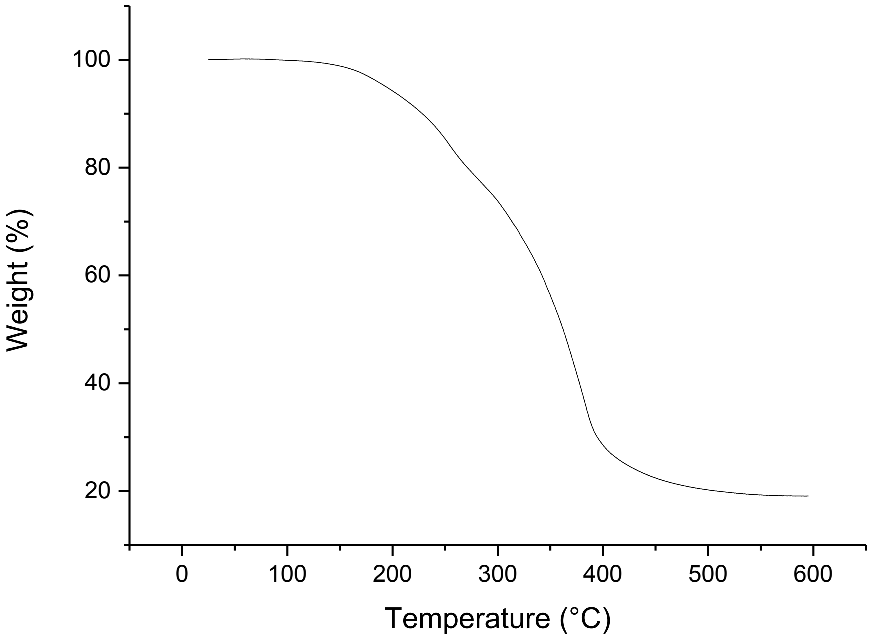

Figure S45. TGA thermogram of **6** (heating rate: 10 °C/min).

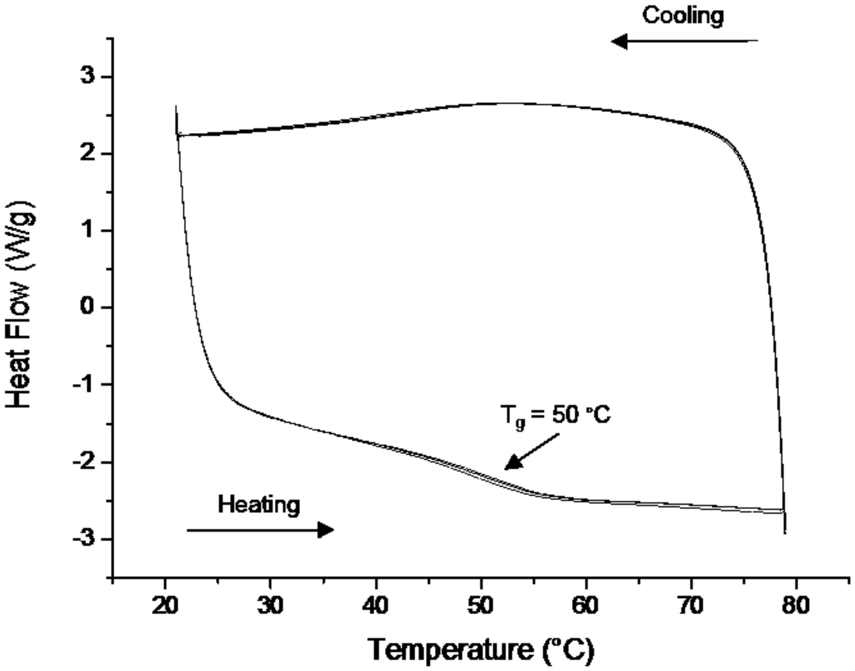

Figure S46. DSC thermogram of **6**, first cycle excluded (heating rate: 10 °C/min).

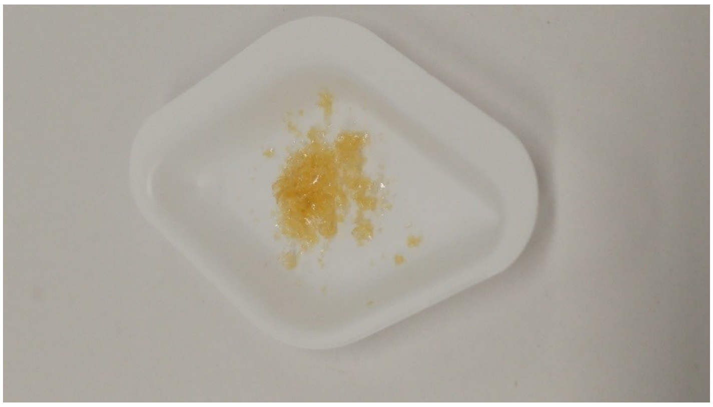

Figure S47. Photograph of isolated **6**.

4.7. Synthesis and characterisation of polymer **7**

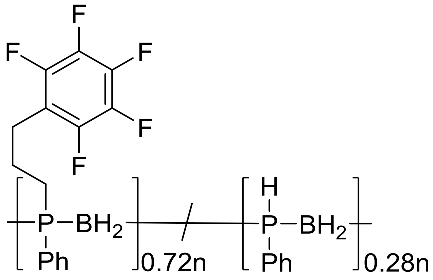

Poly(phenylphosphinoborane) (244 mg, 2 mmol), allyl pentafluorobenzene (307 µL, 2 mmol), DMPAP (51 mg, 0.2 mmol), TEMPO (31 mg, 0.2 mmol), and THF (5 mL) were added to a 14 mL vial equipped with a magnetic stirrer. The resultant solution was irradiated ca. 3 cm away from a mercury lamp for 2 h. Volatiles were then removed under vacuum and the resultant solid was purified by precipitation from DCM into pentane at -78 °C (3 x 25 mL). The polymer was then dried under vacuum at 40 °C for 48 h yielding **7** as an orange solid (conversion: 72%; yield: 204 mg, 37%). **Spectroscopic data**: ^{1}H NMR (400 MHz, $CDCl_3$) δ 7.52 – 6.80 (br m, Ar*H*), 4.09 (br d, *J* = 344 Hz, PhP*H*), 2.24 (br s, $C_6F_5CH_2$), 1.68 – 0.92 (br m, C*H*$_2$, C*H*$_3$, B*H*$_2$); ^{11}B NMR (128 MHz, $CDCl_3$) δ -34.3 (br s); ^{31}P NMR (162 MHz, $CDCl_3$) δ -24.0 (s, 72%, PhR*P*BH_2), -48.6 (d, *J* = 368 Hz, 28%, PhH*P*BH_2); ^{19}F NMR (283 MHz, $CDCl_3$) δ -143.63 (s), -157.72 (s), -162.83 (s); GPC (2 mg/mL) M_n = 130,000 Da, PDI = 1.5; $T_{5\%}$ = 209 °C; ceramic yield = 34%; T_g = 67 °C.

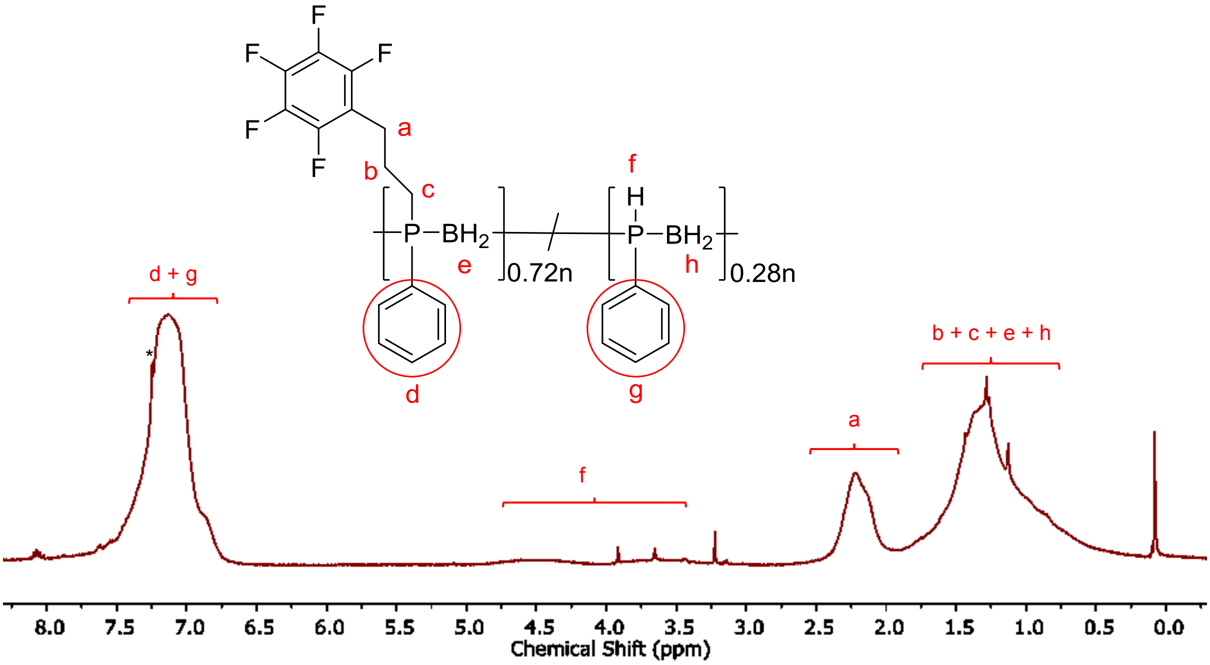

Figure S48. ^{1}H NMR spectrum (400 MHz, $CDCl_3$) of **7**. Deuterated chloroform residual signal denoted by *.

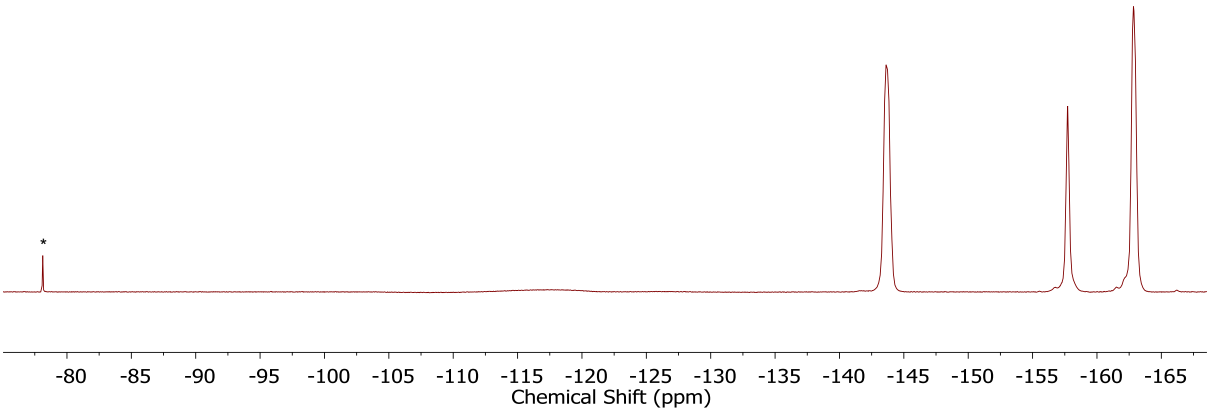

Figure S49. ^{19}F NMR (283 MHz, $CDCl_3$) of **7**. Signal arising from -OTf group originating from $CpFe(CO)_2OTf$ used in the polymerisation of PhH_2PBH_3 denoted by *.

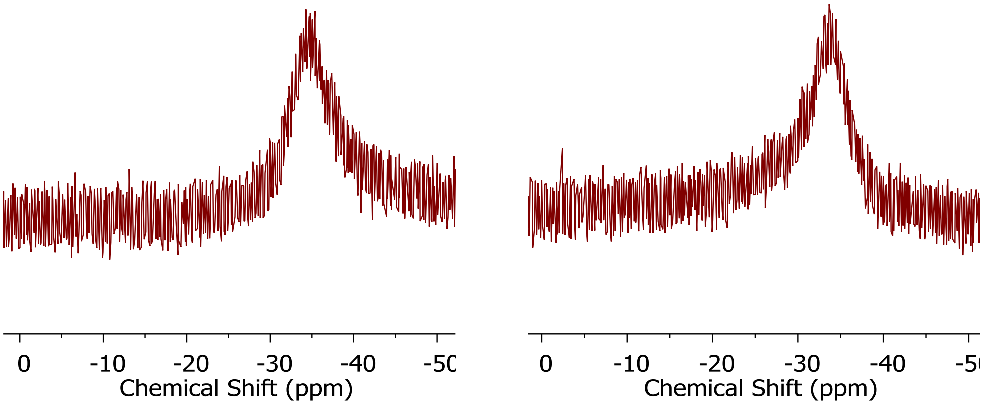

Figure S50. ^{11}B (left) and $^{11}B\{H\}$ (right) NMR spectra (128 MHz, $CDCl_3$) of **7**.

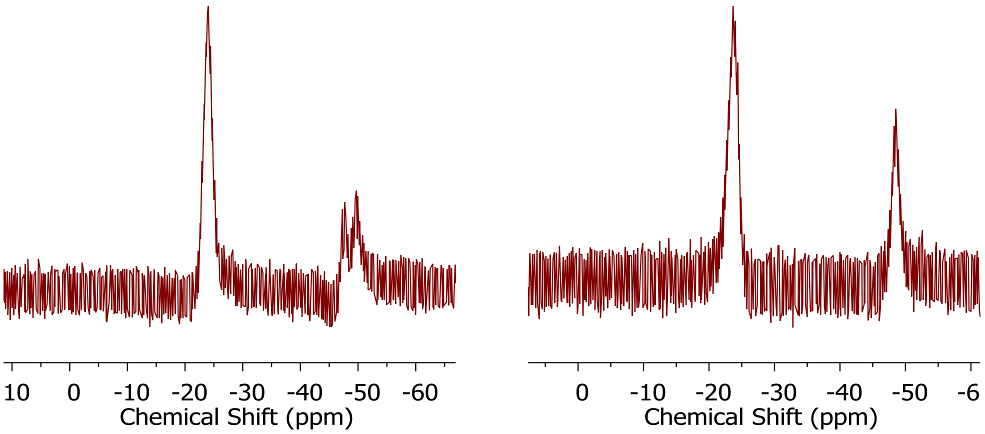

Figure S51. ^{31}P (left) and $^{31}P\{H\}$ (right) NMR spectra (162 MHz, $CDCl_3$) of **7**.

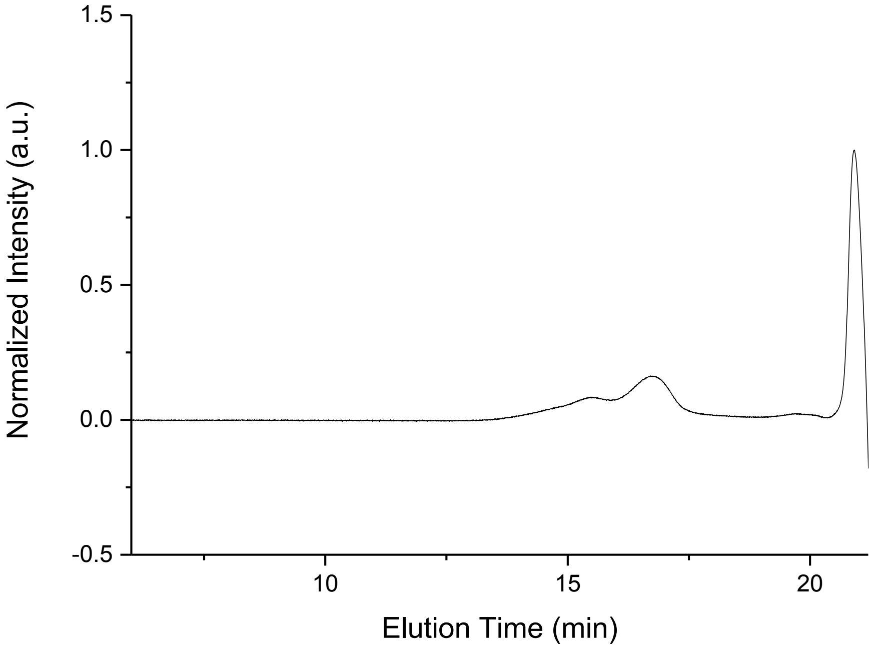

Figure S52. GPC chromatogram of **7** (2 mg/mL in THF, 0.1 w/w % *n*-Bu_4NBr in the THF eluent).

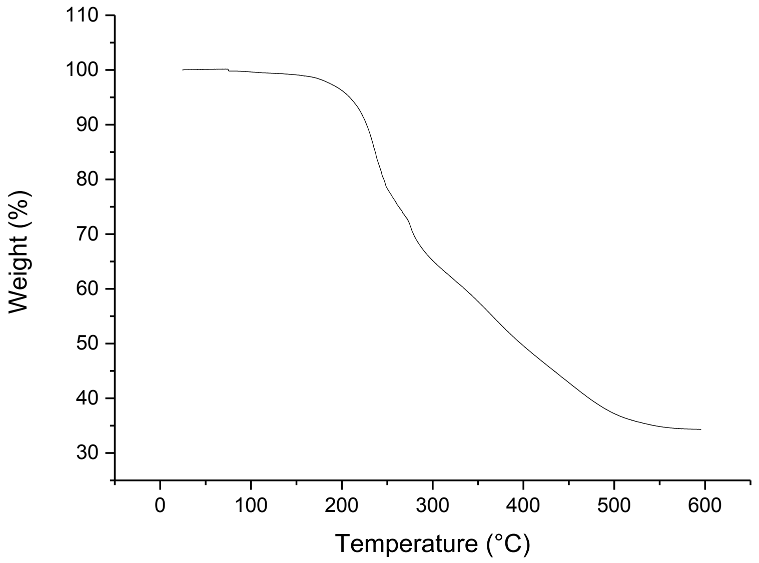

Figure S53. TGA thermogram of **7** (heating rate: 10 °C/min).

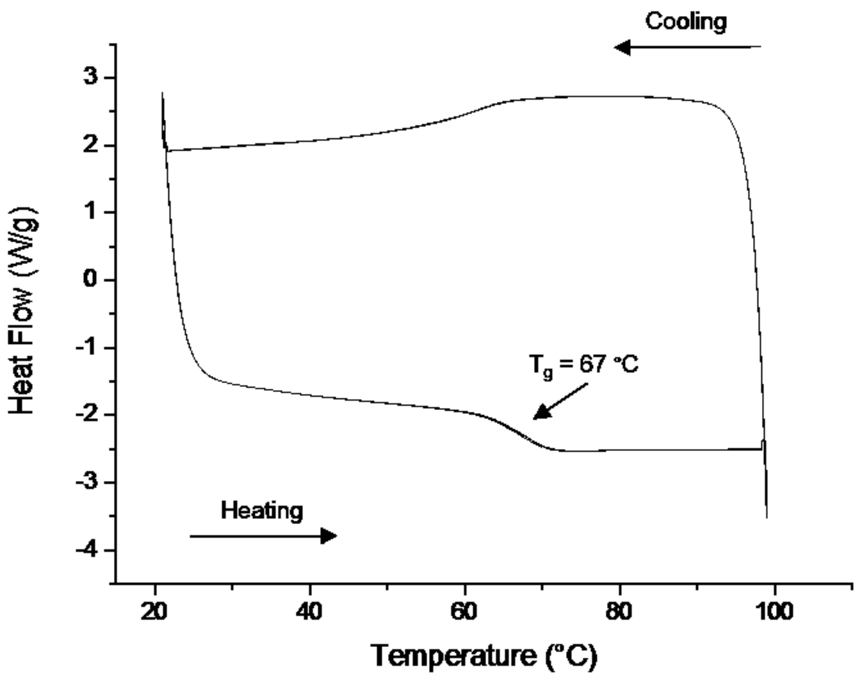

Figure S54. DSC thermogram of **7**, first cycle excluded (heating rate: 10 °C/min).

4.8. Synthesis and characterisation of polymer **8**

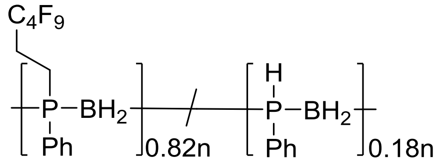

Poly(phenylphosphinoborane) (244 mg, 2 mmol), 1H,1H,2H-perfluoro-1-hexene (339 mg, 2 mmol), DMPAP (51 mg, 0.2 mmol), TEMPO (31 mg, 0.2 mmol), and THF (5 mL) were added to a 14 mL vial equipped with a magnetic stirrer. The resultant solution was irradiated ca. 3 cm away from a mercury lamp for 2 h. Volatiles were then removed under vacuum and the resultant solid was purified by precipitation from THF into H_2O/isopropanol (1:1 v/v) at -20 °C (3 x 25 mL). The polymer was then dried under vacuum at 40 °C for 48 h yielding **8** as an orange solid (conversion: 82%; yield: 479 mg, 74%). **Spectroscopic data**: ^{1}H NMR (400 MHz, $CDCl_3$) δ 7.54 – 6.80 (br m, Ar*H*), 4.16 (br d, *J* = 357 Hz, PhP*H*) 2.28 – 0.94 (br m, C*H*$_2$, B*H*$_2$); ^{11}B NMR (128 MHz, $CDCl_3$) δ -34.1 (br s); ^{31}P NMR (162 MHz, $CDCl_3$) δ -23.7 (s, 82%, PhR*P*BH$_2$), -49.9 (d, *J* = 357 Hz, 18%, PhH*P*BH$_2$); ^{19}F NMR (377 MHz, $CDCl_3$) δ -81.31, -115.17, -124.30, -126.30; GPC (2 mg/mL) M_n = 92,000 Da, PDI = 1.4; $T_{5\%}$ = 173 °C; ceramic yield = 8%; T_g = 43 °C.

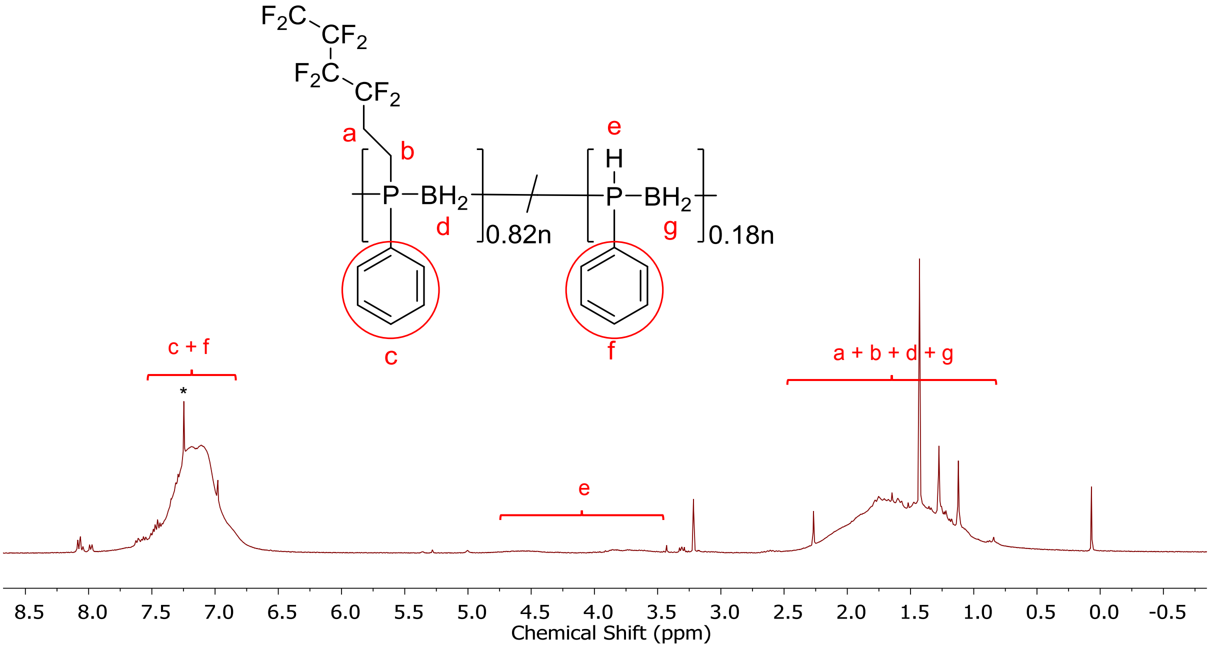

Figure S55. ^{1}H NMR spectrum (400 MHz, $CDCl_3$) of **8**. Deuterated chloroform residual signal denoted by *.

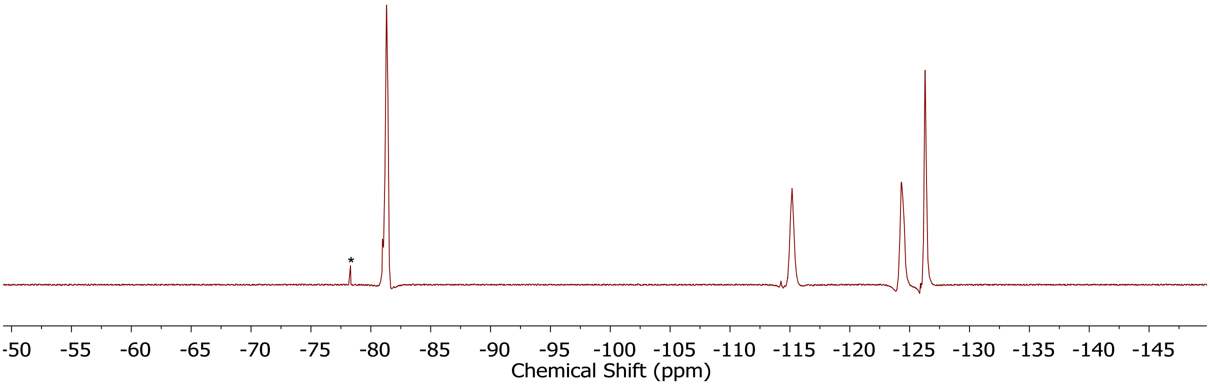

Figure S56. ^{19}F NMR (377 MHz, $CDCl_3$) of **8**. Signal arising from -OTf group originating from $CpFe(CO)_2OTf$ used in the polymerisation of PhH_2PBH_3 denoted by *.

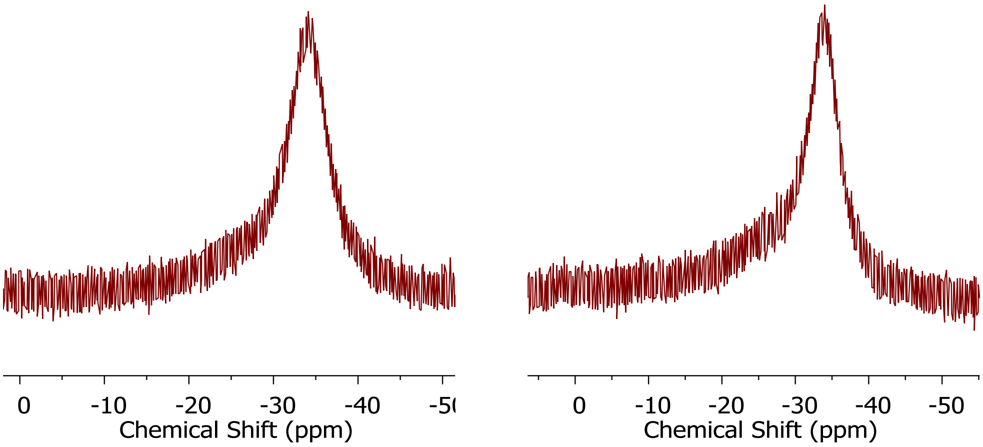

Figure S57. ^{11}B (left) and ^{11}B{H} NMR spectra (128 MHz, $CDCl_3$) of **8**.

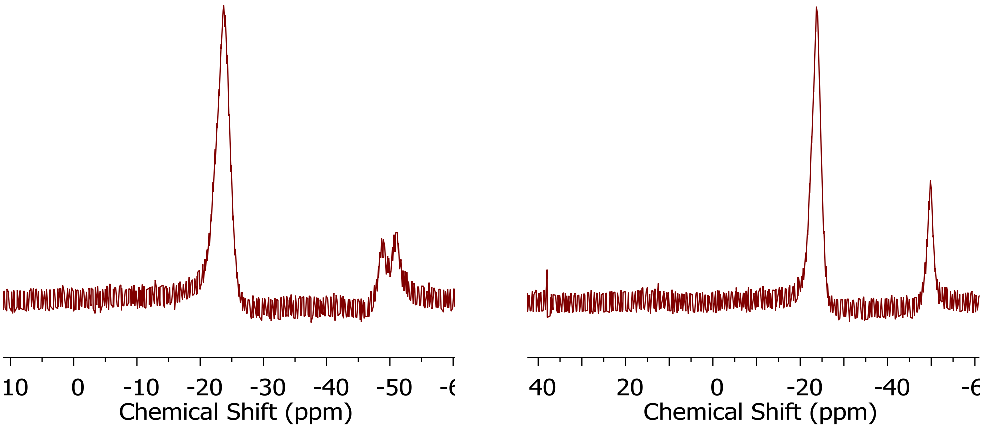

Figure S58. ^{31}P (left) and ^{31}P{H} (right) NMR spectra (162 MHz, $CDCl_3$) of **8**.

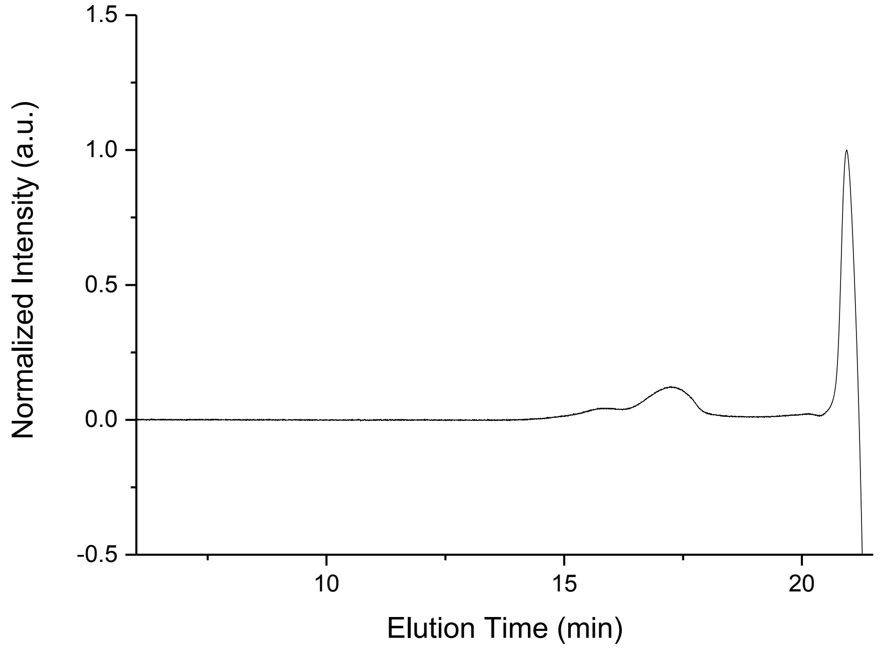

Figure S59. GPC chromatogram of **8** (2 mg/mL in THF, 0.1 w/w % *n*-Bu_4NBr in the THF eluent).

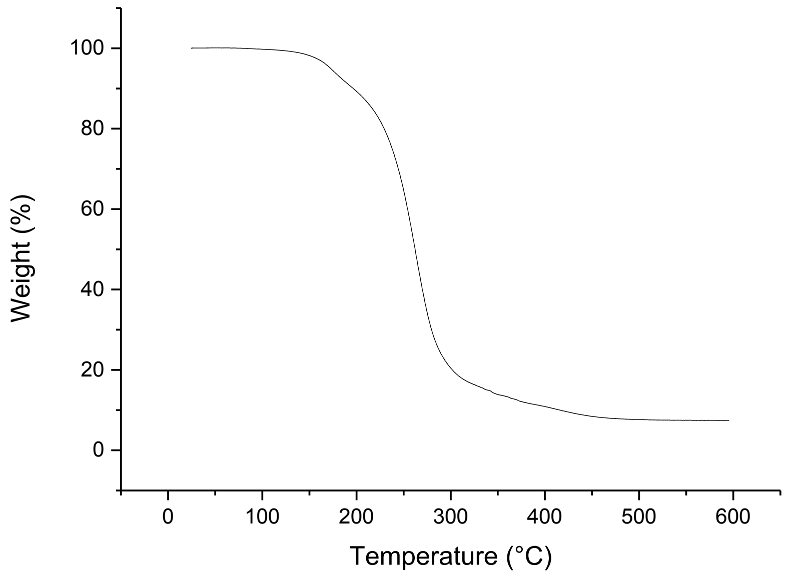

Figure S60. TGA thermogram of **8** (heating rate: 10 °C/min).

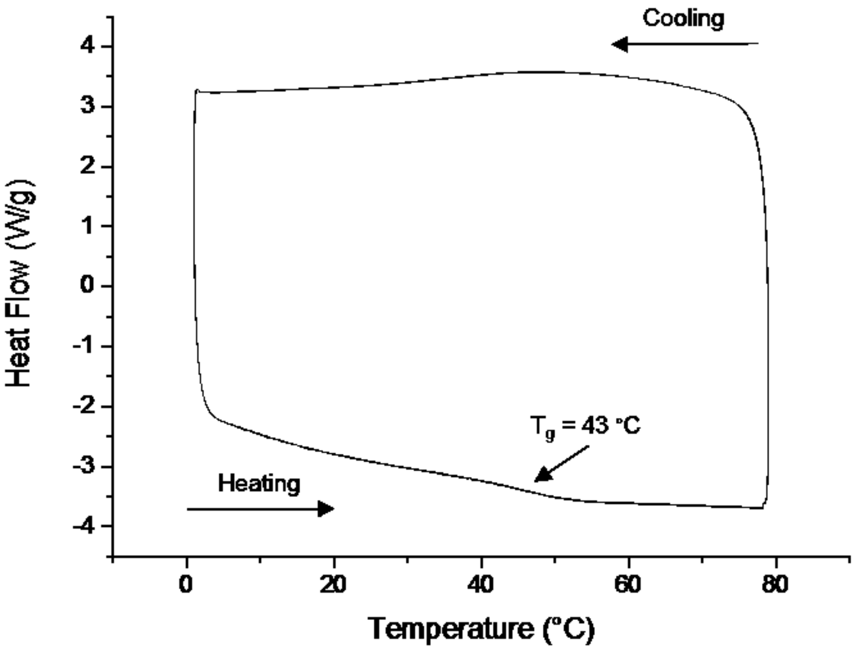

Figure S61. DSC thermogram of **8**, first cycle excluded (heating rate: 10 °C/min).

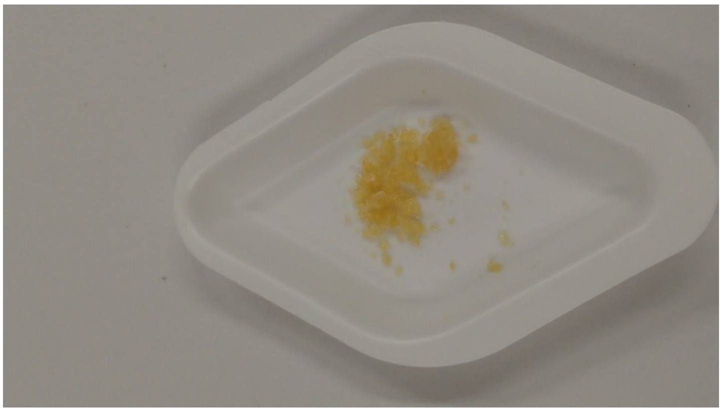

Figure S62. Photograph of isolated **8**.

5. Synthesis and characterisation of crosslinked polyphosphinoborane

5.1. Irradiation of $[PhHPBH_2]_n$ with DMPAP (10 mol%) in the absence of alkene.

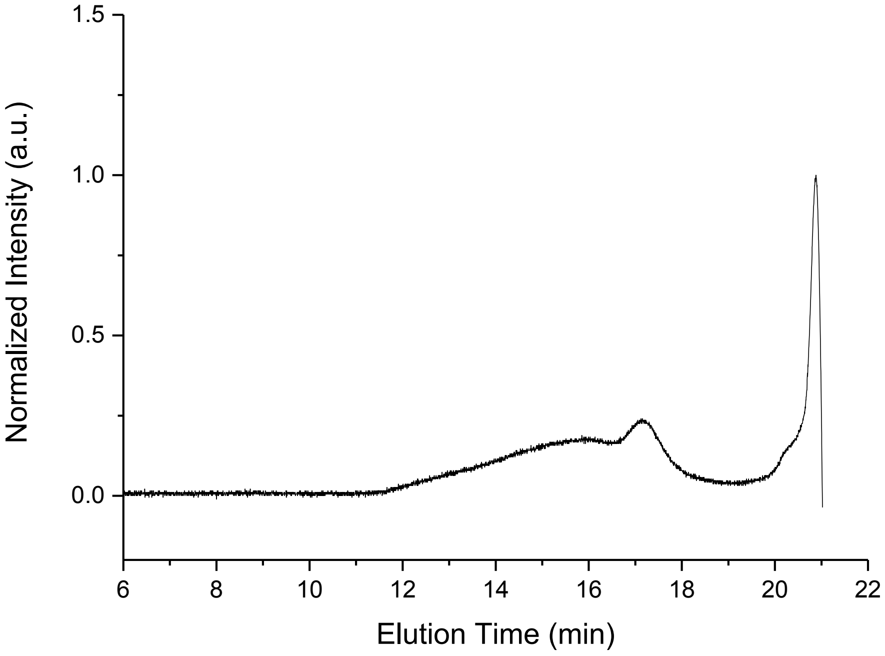

Figure S63. GPC chromatogram in THF (2 mg/mL, 0.1 w/w % *n*-Bu_4NBr in the THF eluent) of material obtained via irradiation of $[PhHPBH_2]_n$ with DMPAP (10 mol%) in the absence of alkene.

5.2. Synthesis and characterisation of crosslinked poly(phenylphosphinoborane)

Polyphenylphosphinoborane (122 mg, 1 mmol), 1,5-hexadiene (17.8 µL, 0.15 mmol), DMPAP (25 mg, 0.1 mmol), TEMPO (15 mg, 0.1 mmol), and THF (1 mL) were added to a 14 mL vial. The resultant solution was irradiated ca. 3 cm away from a mercury lamp for 2 h resulting in the formation of an insoluble gel with exclusion of orange coloured solvent. Excess solvent was decanted away, and volatiles were removed under vacuum yielding an orange solid. This solid was purified by swelling in THF for 6 hours followed by decanting away of excess solvent until no colouration of the solvent was observed (2 x 5 mL). Volatiles were removed under vacuum yielding a brittle orange solid (yield: 89 mg). ^{31}P NMR (202 MHz, THF-d8) δ -23.6 (s, 7%, PhR*P*BH_2), -49.1 (d, J = 345.3 Hz, 93%, $PhHPBH_2$); $T_{5\%}$ = 138 °C; ceramic yield = 53%.

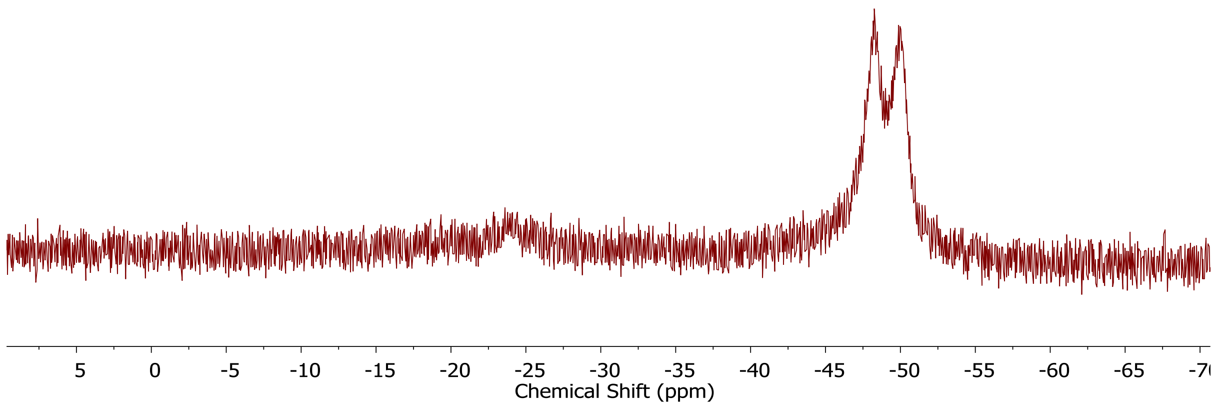

Figure S64. ^{31}P NMR spectrum (202 MHz, THF-d_8) of crosslinked poly(phenylphosphinoborane) swelled in THF-d_8.

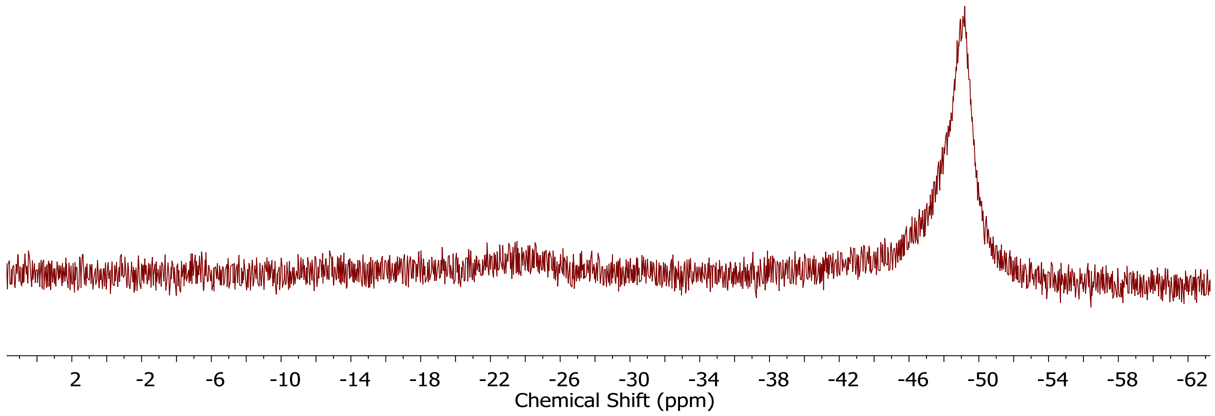

Figure S65. $^{31}P\{H\}$ NMR spectrum (202 MHz, thf-d_8) of crosslinked poly(phenylphosphinoborane) swelled in THF-d_8.

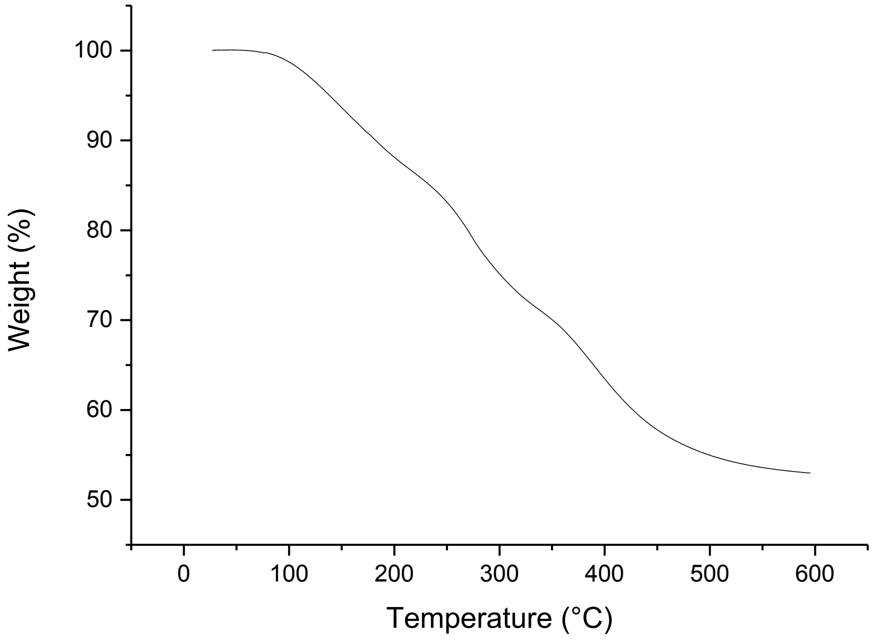

Figure S66. TGA thermogram of crosslinked poly(phenylphosphinoborane) (heating rate: 10 °C/min).

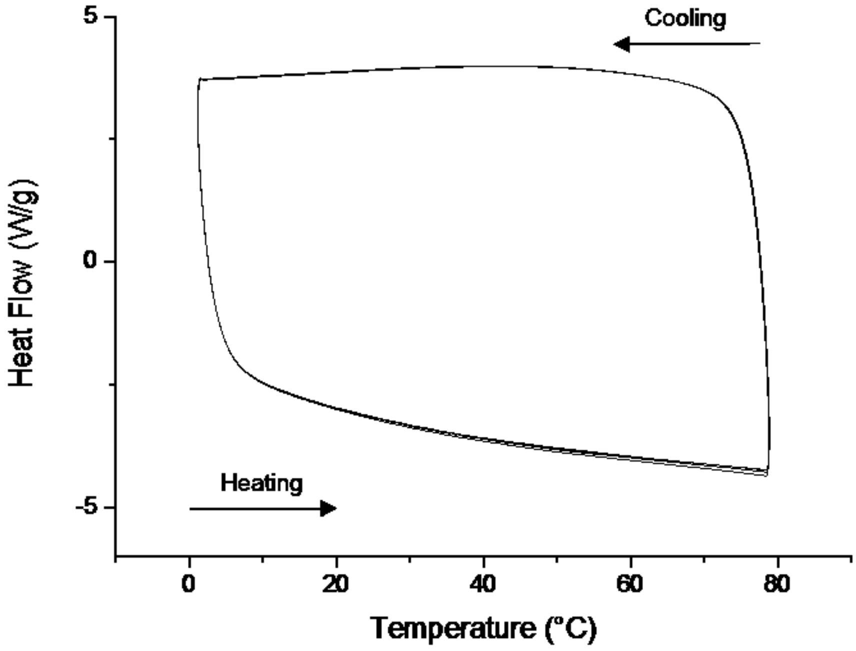

Figure S67. DSC thermogram of crosslinked poly(phenylphosphinoborane), first cycle excluded (heating rate: 10 °C/min).

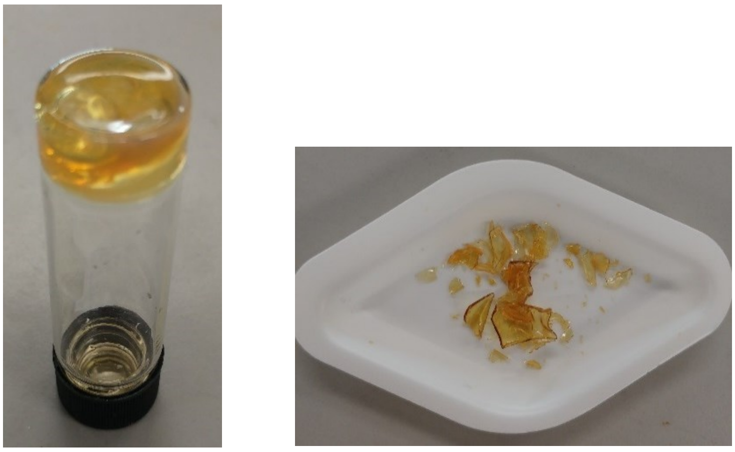

Figure S68. Photograph of crosslinked poly(phenylphosphinoborane) after irradiation (left) and after drying under vacuum for 48 h (right).

5.3. Swellability of crosslinked poly(phenylphosphinoborane)

A sample of dry crosslinked poly(phenylphosphinoborane) was weighed (54 mg) and swelled in THF for 48 h. No coloration of the THF was observed after this time. Excess solvent was decanted away, and surface solvent removed by careful swabbing with a Kimwipe. After this, the sample was reweighed (114 mg) and the swellability in THF calculated (210% mass increase).

6. Synthesis of a water-soluble bottlebrush polymer

6.1. Synthesis and characterisation of bottlebrush polymer **9**

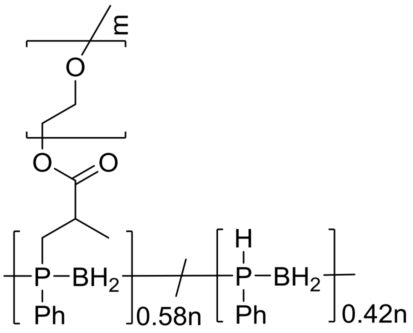

Poly(phenylphosphinoborane) (244 mg, 2 mmol), poly(ethylene glycol) methyl ether methacrylate (average M_n 950 Da, 1.90 g, 2 mmol), DMPAP (51 mg, 0.2 mmol), TEMPO (31 mg, 0.2 mmol), and THF (5 mL) were added to a 14 mL vial equip with a magnetic stirrer. The resultant solution was irradiated ca. 3 cm away from a mercury lamp for 2 h. Volatiles were removed under vacuum and the resultant material was dissolved in water. The yellow solution obtained was transferred into a dialysis tube (MWCO: 12-14,000 g/mol) and dialysis was performed against water for 48 h in order to remove excess poly(ethylene glycol) methyl ether methacrylate. No colouring of the medium outside the dialysis tubing was observed. The yellow solution remaining inside the dialysis tubing was dried under vacuum at 40 °C for 48 h yielding **9** as an orange solid (grafting density: 0.58; yield: 0.93 g, 69%). **Spectroscopic data:** ^{11}B NMR (128 MHz, $CDCl_3$) δ -34.6 (br s); ^{31}P NMR (162 MHz, $CDCl_3$) δ -24.24 (s, PhR*P*BH-$_2$), -48.85 (d, J = 352 Hz, PhH*P*BH_2); GPC (2 mg/mL) M_n = 156,000 Da, PDI = 1.3; T_m (PEG side chains) = 40.0 °C; $T_{5\%}$ = 301 °C; ceramic yield = 2.4%.

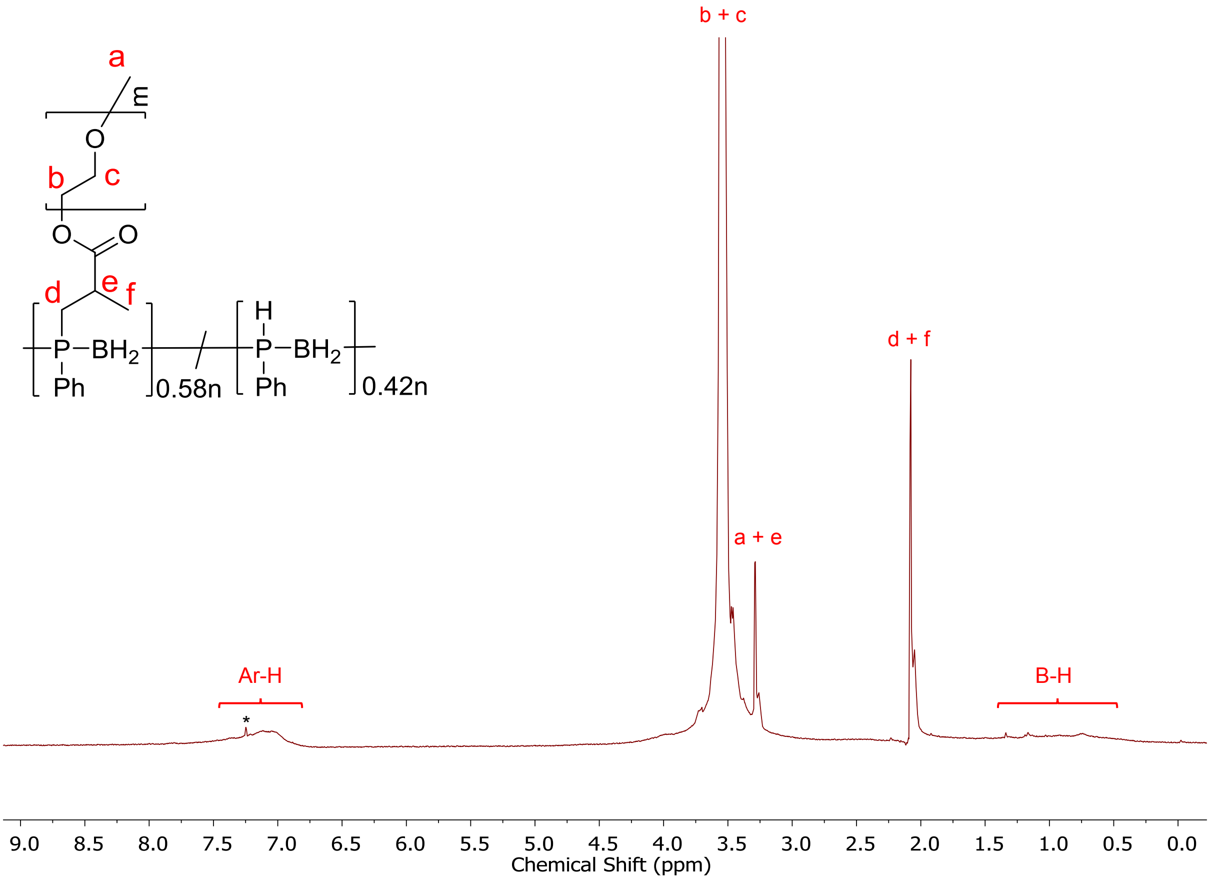

Figure S69. ^{1}H NMR spectrum (400 MHz, $CDCl_3$) of **9**. Deuterated chloroform residual signal denoted by *.

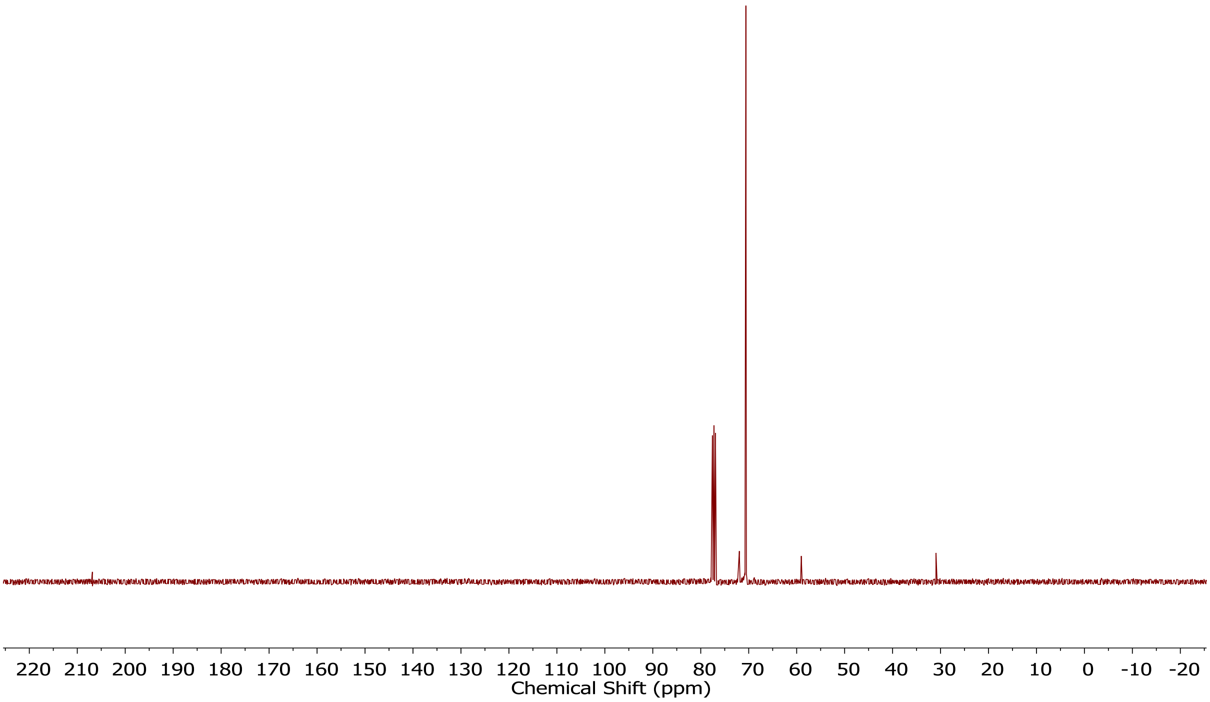

Figure S70. ^{13}C NMR spectrum (126 MHz, $CDCl_3$) of **9**.

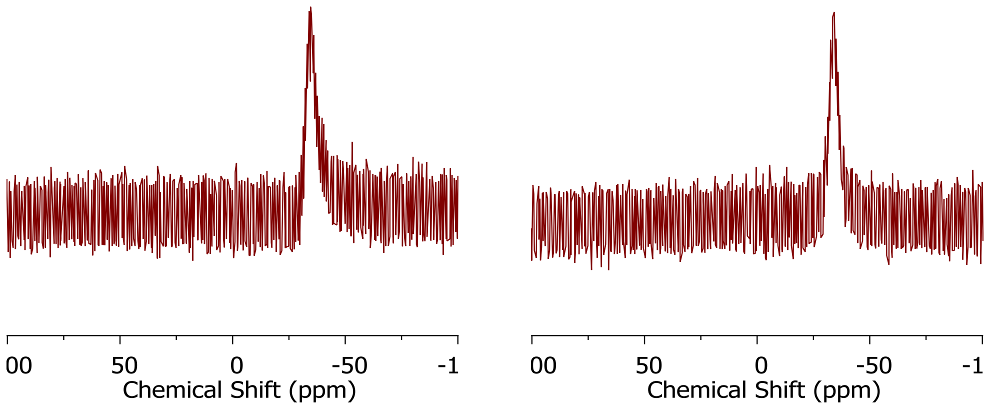

Figure S71. ^{11}B (left) and $^{11}B\{H\}$ NMR spectra (128 MHz, $CDCl_3$) of **9**.

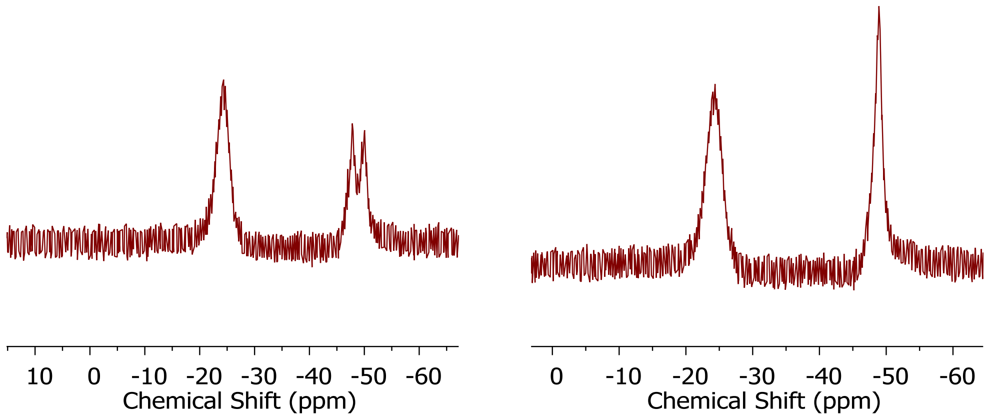

Figure S72. ^{31}P (left) and $^{31}P\{H\}$ (right) NMR spectra (162 MHz, $CDCl_3$) of **9**.

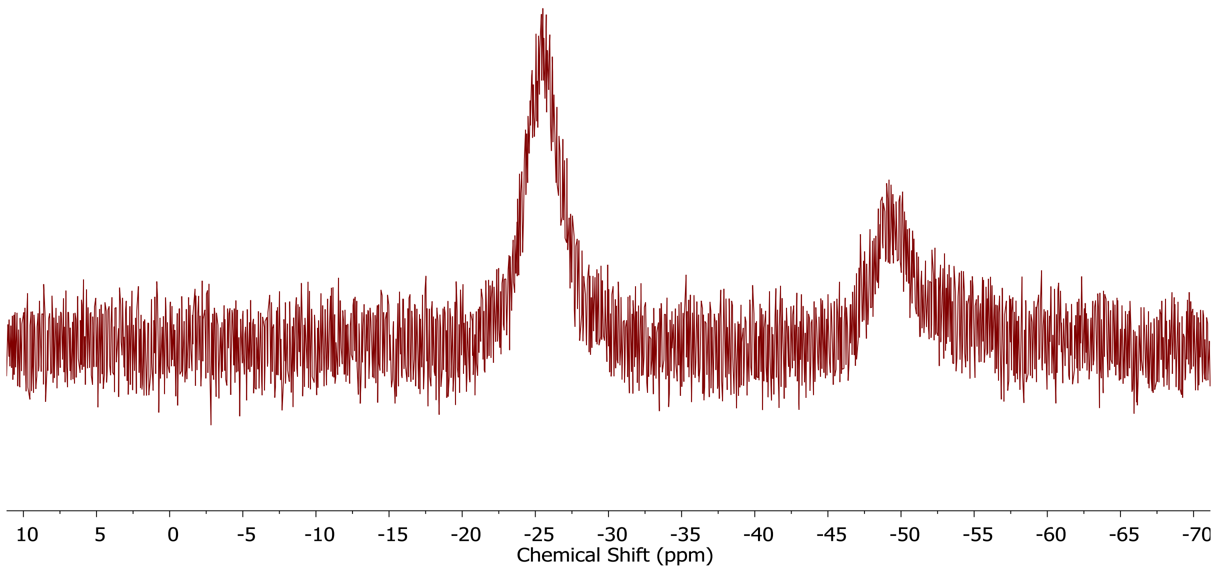

Figure S73. ^{31}P NMR spectrum (203 MHz, D_2O) of **9**. Peak broadening in D_2O prevents the observation of P-H coupling.

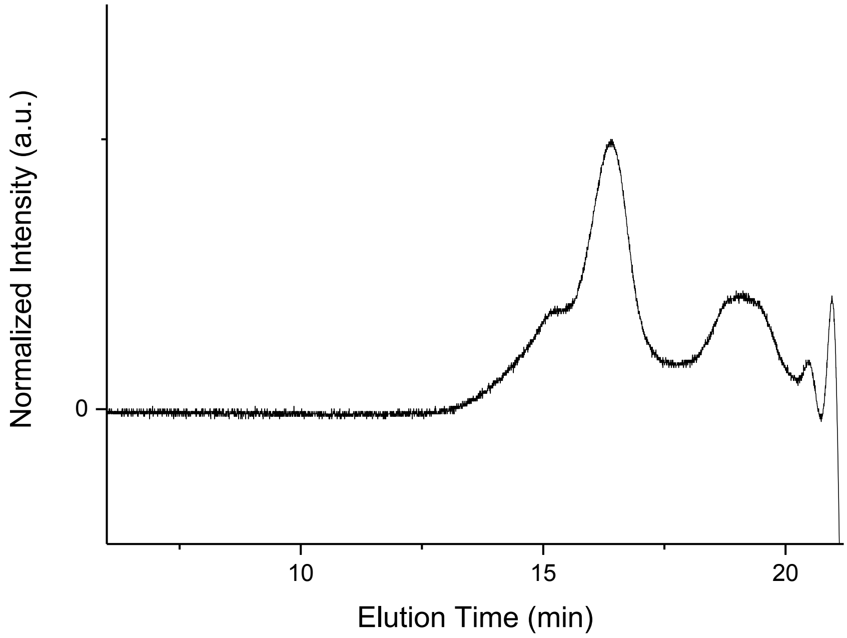

Figure S74. GPC chromatogram of **9** in THF (2 mg/mL, 0.1 w/w % *n*-Bu_4NBr in the THF eluent).

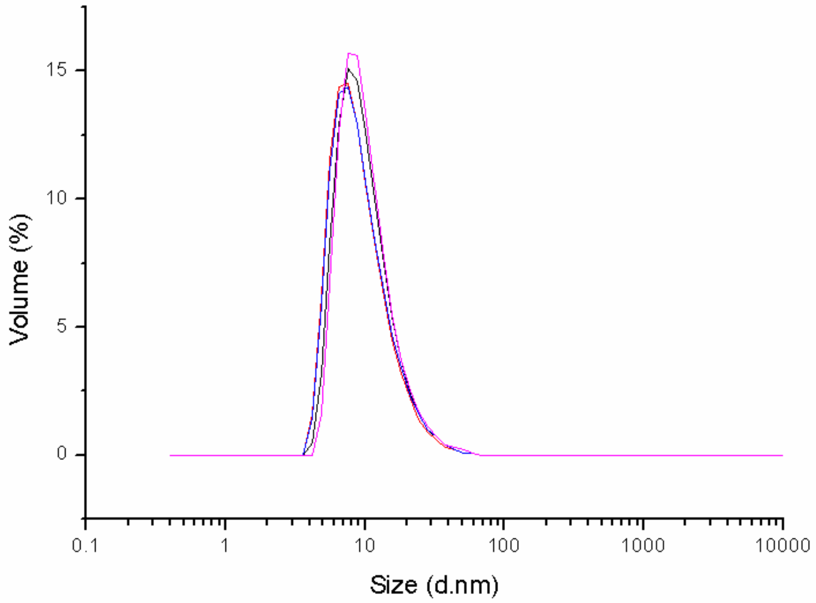

Figure S75. DLS size distribution by volume of **9** in THF (1 mg/mL). Multiple measurements in different colours show that the measured diameter is stable.

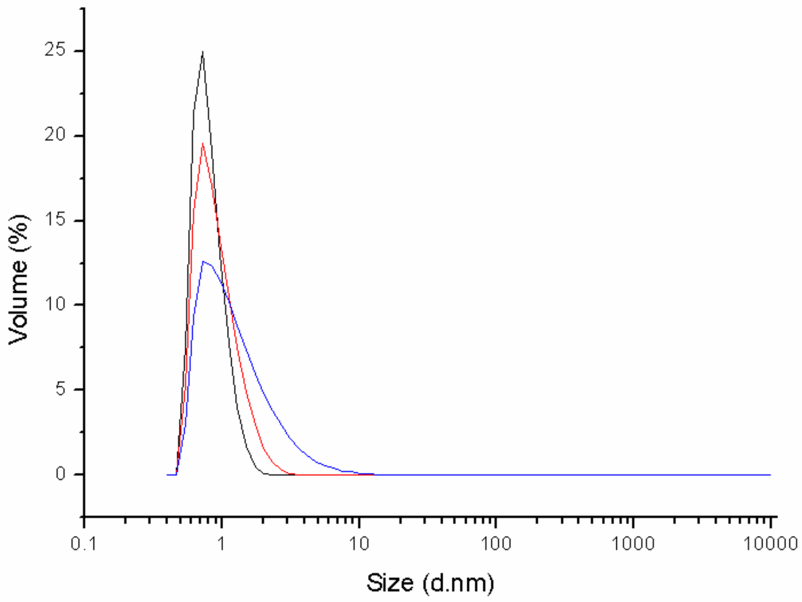

Figure S76. DLS size distribution by volume of poly(ethylene glycol) methyl ether methacrylate (average M_n 950) in THF (1 mg/mL). Multiple measurements in different colours show that the measured diameter is stable.

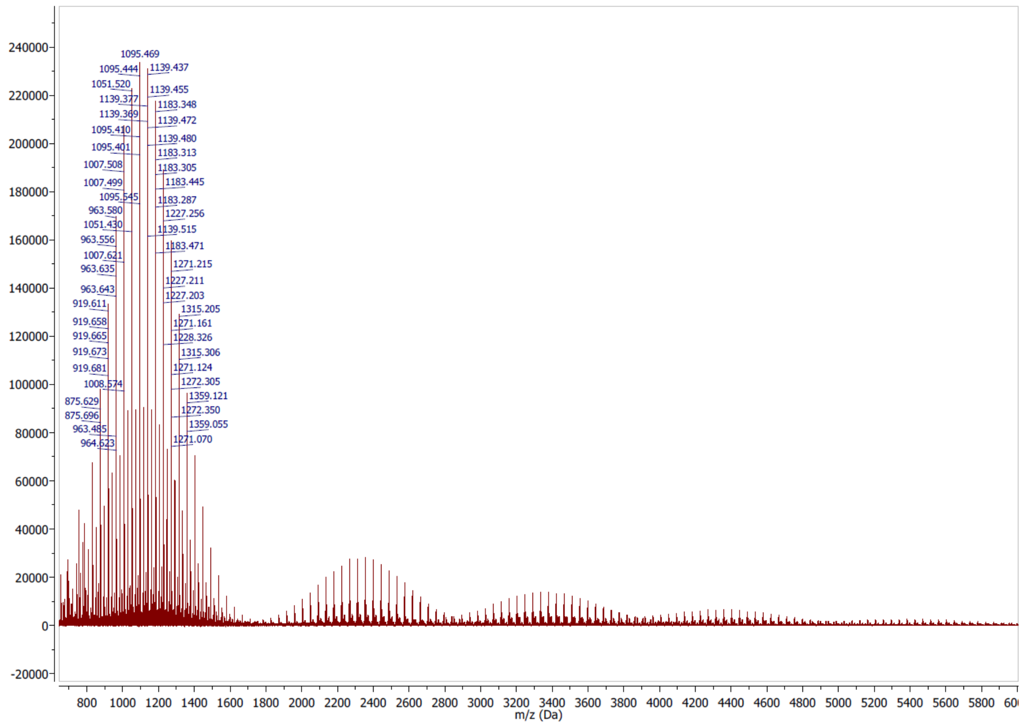

Figure S77. MALDI-MS spectrum of **9**. A complex spectrum is observed due to the presence of both a polymeric backbone and side chains. Each envelope arises from the distribution of molar mass of the polyethylene glycol side chains (molar mass of repeat unit = 44.1 g/mol). Multiple envelopes are observed due to ionisation of the poly(phosphinoborane) main chain units ($PhRPBH_2$, R = $CH_2CH(CH_3)C(O)O[CH_2CH_2O]_mCH_3$, molar mass of repeat unit = 1072 g/mol).

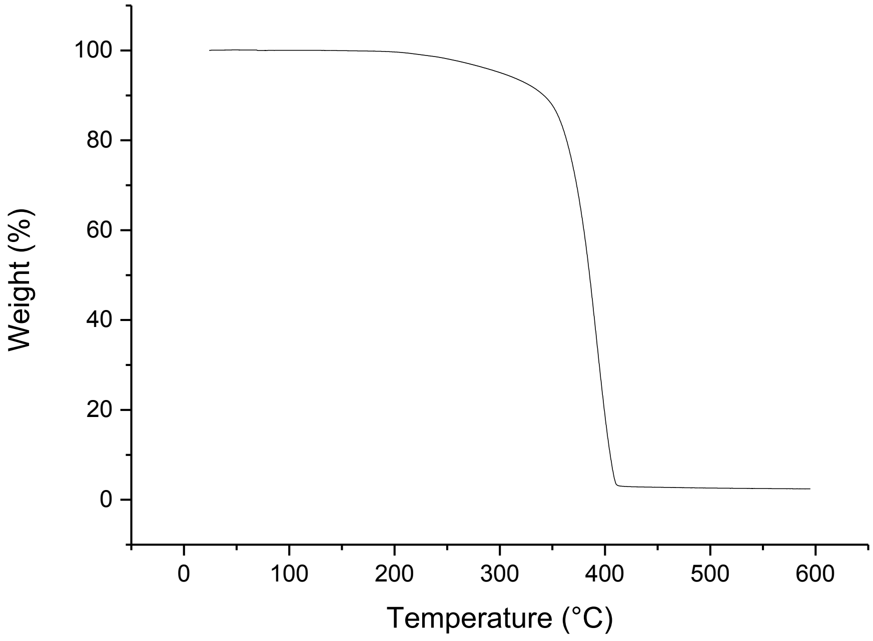

Figure S78. TGA thermogram of **9** (heating rate: 10 °C/min).

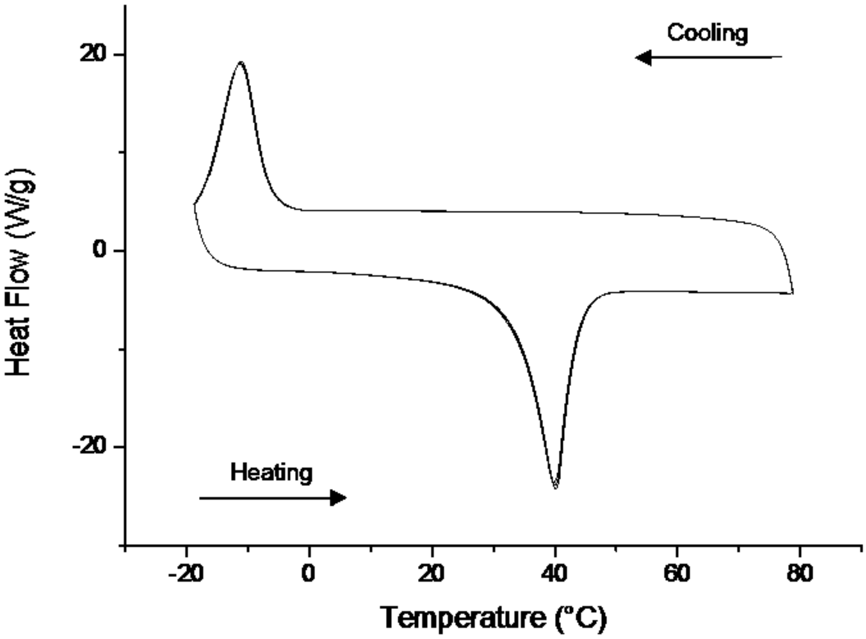

Figure S79. DSC thermogram of **9**, first cycle excluded (heating rate: 10 °C/min).

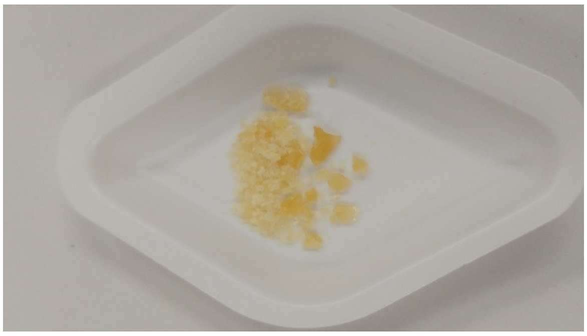

Figure S80. Photograph of isolated **9**.

7. References

1. K. Bourumeau, A.-C. Gaumont and J.-M. Denis, *J. Organomet. Chem.*, 1997, **529**, 205-213.
2. D. J. Liston, Y. J. Lee, W. R. Scheidt and C. A. Reed, *J. Am. Chem. Soc.*, 1989, **111**, 6643-6648.
3. A. Schäfer, T. Jurca, J. Turner, J. R. Vance, K. Lee, V. A. Du, M. F. Haddow, G. R. Whittell and I. Manners, *Angew. Chem. Int. Ed.*, 2015, **54**, 4836-4841.
